# Unveiling the molecular arsenal: genome sequencing and *in silico* secretome analysis of *Fusarium verticillioides* provide insights into maize root rot pathogenesis

**DOI:** 10.3389/fpls.2025.1672761

**Published:** 2025-09-30

**Authors:** José Alberto Narváez-Zapata, Yadira Alaníz-Cuevas, J. Alicia Chávez-Medina, Mariela Guadalupe Espinoza-Mancillas, Ignacio E. Maldonado-Mendoza, Rupesh Kumar Singh, Francisco Roberto Quiroz-Figueroa

**Affiliations:** ^1^ Instituto Politécnico Nacional, Centro de Biotecnología Genómica, Reynosa, Mexico; ^2^ Instituto Politécnico Nacional, Centro Interdisciplinario de Investigación para el Desarrollo Integral Regional (CIIDIR)—Unidad Sinaloa, Guasave, Mexico; ^3^ Centre for the Research and Technology of Agroenvironmental and Biological Sciences, CITAB, InovAgro, Universidade de Trás-os-Montes e Alto Douro, UTAD, Quinta de Prados, Vila Real, Portugal

**Keywords:** *Fusariosis*, genomic, lytic enzymes, omic, NGS, Zea mays, SEM

## Abstract

**Introduction:**

*Fusarium verticillioides* (*Fv*) is a major phytopathogen responsible for maize root rot, affecting crop productivity globally. A probable infection mechanism has been suggested in *Fusarium*, involving the disruption, and partial degradation of the plant cell wall by the colonizing fungal hyphae.

**Methods:**

In this study, the highly virulent *Fv* DA42 strain was subjected to whole-genome sequencing, *in silico* secretome analysis and SEM structural analysis to elucidate its pathogenic mechanism.

**Results:**

The assembled genome comprised 175 contigs (=200 pb) totaling 42.27 Mb, with an N50 of 1.24 Mb and GC content of 48.51%. A total of 14,198 protein-coding genes were predicted, of which 997 (7.03%) correspond to classical secreted proteins. The predicted secretome includes 262 carbohydrate-active enzymes (CAZymes), 62 proteases, 400 effectors, 481 virulence factors and 288 uncharacterized proteins. Functional annotation revealed enrichment in enzymatic activities such as pectinesterases, feruloyl esterases, and glucosidases, highlighting their role in host cell wall degradation. Chromosomal distribution showed secretome genes concentrated on chromosomes 4 and 8, with the highest density (49.2 genes/Mb) on chromosome 10. Scanning electron microscopy confirmed degradation of maize root hairs and epidermis seven days post-infection, this degradation may have occurred days prior to the observation. STRING analysis identified key proteins like FVEG_10795 (pectinesterase) and FVEG_09361 (feruloyl esterase) as central to coordinated enzymatic attacks.

**Discussion:**

This integrative analysis offers crucial insights into *Fv* pathogenicity and provides a molecular basis for targeted antifungal strategies and resistance breeding in maize.

## 1 Introduction

Maize root rot is an important fungal disease, which is a constant menace to maize agriculture worldwide. The main fungal phytopathogen associated with root rot are *Fusarium* spp. However, the *Fusarium* incidence statistics in maize rots is limited and it varies by geographic location, climate (humidity and temperature) during the agriculture cycle, agricultural practices, and the maize genetic background (Czarnecka et al., 2022). Additionally, visual identification of root infection is not easy. Reports show prevalence of this phytopathogen in corn field from diverse agriculture regions around the world, for example, Serbia, Croatia (Hajnal et al., 2023), Portugal (Simoes et al., 2023), Poland (Czembor et al., 2015), China (Xia et al., 2022), Germany (Pfordt et al., 2020), and Mexico (Velarde Félix et al., 2018).

*Fusarium* species also cause other types of rot and blight, affecting seedling health, stalk resistance, and grain quality (Baldwin et al., 2014; Gai et al., 2018; Okello et al., 2019). *Fusarium* maize rots are caused by several species (Leyva-Madrigal et al., 2015; Okello et al., 2019), with the most predominant being *F. graminearum* Schwabe and *F. verticillioides* Sacc. Nirenberg (Hussein et al., 2003; Görtz et al., 2008; Atanasova-Penichon et al., 2014; Leyva-Madrigal et al., 2015; Qiu et al., 2015; de Sousa et al., 2022).

*Fusarium verticillioides* (*Fv*) like many fungal pathogens, possesses a remarkable ability to infect maize plants at every developmental stage, from seed and seedling through the productive stage (Roman et al., 2020). The infectious potential of *Fusarium* is evident in its ability to infiltrate not only in damaged roots but also in stalks, and even developing grains through the stigmas and silk channels (Duncan and Howard, 2010; Gai et al., 2018; Thompson and Raizada, 2018), suggesting that this phytopathogen could utilize diverse infection strategies (Beccari et al., 2022). In soil, the root cell wall is the first barrier that soilborne pathogens, such as *Fv*, must overcome to infect plants. The mechanism of infection in tomato and pea roots by *F. oxysporum* occurs mainly through cell-to-cell spaces, lateral root emergence, or cell damage caused by rocks or insects. The hyphae enter by disrupting, and causing partial degradation of the cell wall (Bishop and Cooper, 1983). These hyphae form appressorium-like infection structures or penetration pegs. These structure have also been visualized in wheat coleoptiles (Qiu et al., 2019) and floral organs (Boenisch and Schafer, 2011) colonized by *F. graminearum* and *F. culmorum* (Kang and Buchenauer, 2002), as well as by other fungal necrotrophic phytopathogens, such as *Botrytis cinerea* (Dinh et al., 2011) and *Rhizoctonia solani* (Chethana et al., 2021). Using SEM and red or green fluorescent protein tags, *F. verticillioides* has been shown colonizing maize by penetration of the lateral root breakage zones, and root epidermis through appressorium-like structures (Murillo et al., 1999; Oren et al., 2003; Wu et al., 2011). During the first days of *Fusarium* root rot infection, symptoms start as small pale-yellow root discolorations that progress to dark brown color and eventually form necrotic spots (Wu et al., 2011; Quiroz-Figueroa et al., 2023). Simultaneously, root hairs collapse, providing a visual cue that suggests events associated with cell wall degradation in the epidermal cells caused possibly by *Fv* secreted proteins (Quiroz-Figueroa et al., 2023). This intricate process of invasion and colonization may shed light on the dynamic interplay between *Fv* and maize, highlighting the importance of understanding the physiological and molecular aspects of this bipartite interaction.

The plant cell wall is a complex structure primarily composed of polysaccharides such as cellulose, hemicellulose, pectin, and lignin as well as proteins and phenolic compounds, such as ferulic acid. These components provide structural support by crosslinking different polysaccharides (Alberts et al., 2002). Fungal phytopathogens, such as *Fv*, secrete an arsenal of lytic enzymes and effector proteins, collectively known as the protein secretome. These proteins act synergistically to degrade cell wall components, which is crucial for nutrient acquisition, fungal colonization, and dispersion within plant tissues (McCotter et al., 2016; Bradshaw et al., 2021). The secretome consists of proteases, lipases, protein effectors, and carbohydrate-active enzymes (CAZymes), such as cellulases, xylanases, and pectinases (Bradshaw et al., 2021). Understanding the mechanisms that *Fv* and other phytopathogens use to degrade the cell wall and overcome host defenses will provide novel targets for disease control.

Whole-genome sequencing (WGS) has revolutionized fungal pathogen research, enabling advancements in disease diagnostics and the determination of evolutionary relationships between species (Navale et al., 2022). Additionally, WGS offers a comprehensive view of potential genetic factors. Next-generation sequencing (NGS) technologies facilitate the identification of numerous genes, including those involved in pathogenesis and secretome gene products. *In silico* analysis of the secretome, which predicts secreted gene products from gene sequences, allows for the identification of key lytic enzymes and protein effectors involved in plant cell wall degradation and pathogenic mechanisms. Six *Fv* genome assemblies have been deposited at NCBI, ranging from contigs to chromosome level, with genome sizes ranging from 41.84 to 44.65 Mb and containing between 15,053 to 20,574 protein-coding genes (Ma et al., 2010; Navale et al., 2022). Recently, a report was published on the gapless genome assembly of *Fv* strain 7600 using PacBio HiFi technology. The resulting final genome assembly is 41.994 Mb and contains 15,230 protein-coding genes (Yao et al., 2023). This genome assembly represents an improvement over the first version, which had a size of 41.79 Mb and contained 14,335 protein-coding genes, along with other genome statistics (Ma et al., 2010).

The *Fv* secretome could plays an important role in plant pathogenesis (Quiroz-Figueroa et al., 2023) and human Fusariosis (Georgiadou et al., 2014), as well as in biotechnological potential uses. Despite the importance of this fungal species, to our knowledge, there are only a few reports on the *Fv* secretome. One of these reports shows the potential for commercial use of *Fv* secretome as a complement to commercial cellulases cocktail in the saccharification (lignocellulose hydrolysis) of wheat straw, 166 *Fv* secreted proteins were identified by proteomic analysis, of which 57 are involved in the degradation of lignocellulosic material, these include CAZymes that degrade cellulose, xylans, pectins, lignins; and other carbohydrate-modifying enzymes (Ravalason et al., 2012). Another report involved the *in silico* comparative analysis of the *Fv* BIONCL4 strain, which identified 2,058 proteins related to the secretome. Of these, 676 corresponded to the classical secretion pathway, while 569 were identified as pathogenesis-related proteins (Navale et al., 2022).

Roots act as the first line of defense against soil pathogens such as *F. verticillioides.* The pathogen has evolved mechanisms to degrade the root cell wall, allowing its entry and subsequent colonization of the plant. This occurs mainly through the secretion of hydrolytic enzymes and effectors that turn off plant immune system (Jones and Dangl, 2006) and break down structural components (Pinto et al., 2025) such as proteins, cellulose, hemicellulose, lignin and pectin, facilitating its dispersion inside the plant tissues and the establishment of infection. In this study, we used SEM, WGS and *in silico* secretome analysis to investigate the molecular arsenal of *Fv* and its significant role in maize root rot. Our aim was to identify potentially key secreted proteins involved in pathogenesis and cell wall degradation. This study could provide the basis for the development of more effective control strategies for the *Fv*-maize interaction and contribute to the protection of this important crop, as well as other crops affected by *Fusarium* species.

## 2 Materials and methods

### 2.1 Seed infection

To evaluate the response to *F. verticillioides* (*Fv*) infection, a maize inbred line highly susceptible (IL09) to root rot was used and previously characterized by Román (2017) and Roman et al. (2020). The virulent *Fv* DA42 strain, described by Leyva-Madrigal et al. (2015) was used for infection assays. Seed preparation followed protocols previously described by Warham et al. (1996) and Román (2017), with minor modifications. Seeds were superficially disinfected by sonication for 5 minutes in sterile distilled water with Tween 20 (5 drops/100 mL) using an ultrasonic bath (2.8 L, Fisher Scientific). This was followed by immersion in 1.5% sodium hypochlorite at 52°C for 20 minutes in a thermostatic water bath (FE-377, Felisa), and then rinsed thrice with sterile distilled water under aseptic conditions in a biological safety cabinet (Herasafe KS, Thermo Scientific).

The *Fv* strain was cultured on Spezieller Nährstoffarmer agar (SNA) medium (Leslie and Summerell, 2007) supplemented with a 1 cm² filter paper and incubated at 25 ± 2°C for 14 days. Conidia were harvested by adding 5 mL of sterile 0.8% NaCl solution to the culture, followed by gentle agitation. A working conidial suspension was prepared at 1 × 10^6^ conidia/mL, quantified using a Neubauer counting chamber (cat. No. 3110, Hausser Scientific, USA) and a light microscope (B-383-M11, Optika, Italy). Disinfected seeds were immersed for 5 minutes in the conidial suspension, while control seeds were treated with sterile water. Ten seeds were placed every 2 cm along sterile, moistened Kraft paper sheets (40 × 20 cm), which were rolled and placed in plastic bags. Seed germination was carried out at 25°C under a 16:8 h light:dark photoperiod for 7 days. Moisture was maintained by daily irrigation with 15 mL of sterile water. Daily visual observations and photographs were taken using a stereo microscope (M205FA, Leica, Germany).

### 2.2 Microscopy analyses

Primary root tissues with and without necrosis symptoms were harvested for scanning electronic microscopy (SEM) studies. These samples were treated as previously described by Olivares-Garcia et al. (2020) and observed using an FEI-Quanta250 FEG microscope (Czech Republic) operated at an acceleration voltage of 5 kV.

### 2.3 DNA isolation and sequencing

The *Fv* DA42 strain culture (Leyva-Madrigal et al., 2015) was grown in Petri dishes containing potato dextrose broth (Cat. 7041, MCD Lab) and incubated at 25 ± 2°C. After seven days of incubation, the mycelium was harvested by scraping it with a spatula. Approximately 100 mg of mycelium was used for genomic DNA (gDNA) extraction using the CTAB protocol (Zhang et al., 1998). The quality of gDNA was evaluated on a 0.8% agarose gel stained with GelRed (cat. 41003, Biotum) and visualized using a Gel Doc XR+ (BioRad). Genomic DNA was quantified with a Nanodrop spectrophotometer (Nanodrop 8000, Thermo Fisher) and stored at -20°C. The DNA samples were prepared according to the NGS library preparation workflow and sequenced using the Illumina platform by Macrogen (whole genome *de novo* sequencing, paired-end reads with a read length of 151).

### 2.4 Assembly of raw data and gene and secretome prediction

Raw reads quality was evaluated using FastQC (Andrews, 2023) and trimmed to remove low quality bases using Trimmomatic (Bolger et al., 2014) with default parameters for paired-end data. These trimmed raw reads were filtered by performing a BLASTn search against the mitochondrial genome of the teleomorph of *Fv* (*Gibberella moniliformis*) (NCBI RefSeq: NC_016687.1). High quality reads were then assembled *de novo* using the Shovill tool (Seemann, 2017) with assemblers, where the Velvet assembler gave the best metrics. The quality of the assembled genome was verified by QUAST (Gurevich et al., 2013), which provides key metrics such as genome size, N50, L50, the number of contigs, and the largest contig. As a second filter to remove potential mitochondrial contaminants, the contigs were BLASTed against a NR mitochondrial database (https://ftp.ncbi.nlm.nih.gov/genomes/refseq/mitochondrion), and those showing ≥90% identity was manually removed. Gene (nt) and protein (aa) predictions were performed using AUGUSTUS through gene models (Stanke and Waack, 2003), which were built from the *Fv* 7600 strain (GCA_000149555.1) using Train Augustus. These programs were implemented on the Galaxy USA platform (Galaxy, 2024). The predicted proteins were functionally annotated by eggNOG-mapper databases (Huerta-Cepas et al., 2019). RefSeq protein annotation was supported by local blast (rblast Version 0-99.4) using the GCF_000149555.1_ASM14955v1_protein.faa database with the argument “max_target_seqs 1” in Rstudio version 4.3.3. The UniProt database (UniProt, 2025), considering *Fv* 7600 (Taxon ID 334819) was used to support the FVEG gene name of the selected protein dataset.

Subcellular localization was predicted for this dataset using Wolf PSORT (Horton et al., 2007). Signal peptides were predicted using DeepSig (Savojardo et al., 2018). These programs were implemented on the Galaxy Europe platform (Galaxy, 2024). Transmembrane proteins were predicted using Phobius by forcing the predictor to choose between two submodels (Kall et al., 2007). Proteins lacking glycosylphosphatidylinositol (GPI) were predicted using the PredGPI web tool (Pierleoni et al., 2008). Carbohydrate-active enzyme (CAZymes) were predicted in the DBCAN3 web server (Zheng et al., 2023). Effector proteins were determined using EffectorP-fungi 3.0 (Sperschneider and Dodds, 2022), while virulence factors were predicted using the pathogen–host interactions database PHI-base (Urban et al., 2022) with NCBI BLAST+ blastp (Cock et al., 2015) on the Galaxy platform. Phylogenetic analysis of the filtered proteins was conducted in MEGA11 (Tamura et al., 2021) using the UPGMA method.

### 2.5 Genome representation

Marker positions of the filtered protein dataset were obtained from the GFF file of the *Fv* 7600 strain (GCA_000149555.1) and then imported into R version 4.3.3. The qtl and LinkageMapView packages (Ouellette et al., 2018) were loaded for linkage map analysis and visualization. The maximum position (maxpos) across chromosomes 1 to 11 was calculated, and an axis-tick vector (at.axis) from 0 to maxpos in 1 cM increments was generated, along with a label vector (axlab) at 20 cM intervals. Marker density was estimated using lmvdencolor with an RColorBrewer color palette. Finally, all chromosomes were plotted with marker labels (dupnbr=TRUE) and a density gradient (denmap=TRUE) using lmv.linkage.plot with “Chromosome %” as the unit.

### 2.6 GO and KEGG analysis

The online DAVID software (Sherman et al., 2022) was used to obtain the gene ontology (Biological processes) of the filtered proteins. GO terms and biological processes were summarized and consolidated to build a specific functional database. This database was used with the GGPLOT2 package to build a GO bar plot indicating the most abundant GO terms and its functions. GGPLOT2 in Rstudio version 4.3.3. was used to build a bar plot showing the number of proteins identified in each pathway.

### 2.7 String network analysis

Based on their reported function, a subset of the selected amino acid sequences with assigned gene names was mapped in the STRINGdb (https://string-db.org/) using the rba_string_map_ids function from rbioapi package, specifying the specie ID: 334819 (*Fv*) in Rstudio version 4.3.3. A selection of the STRING IDs corresponding to “high identity sequences to the PHI-base” was used to retrieve the interaction network for each protein using the rba_string_interaction_partners function. The top ten protein interactions were selected based on their scores for each chosen protein, and a subset of proteins was visualized using the rba_string_network_image function.

### 2.8 Phylogenetic analysis of *Fusarium* genomes

*Fusarium verticillioides* genomes (GCA013759275.1, GCA020882315.1, GCA003316975.1, GCA017309895.1, GCA_033110985.1, and GCA000149555.1) were retrieved from GenBank and analyzed alongside the *Fv* DA42 genome using whole-genome phylogeny with the ParSNP tool (Kille et al., 2024), with the GCA000149555.1 genome as reference. The resulting Newick file was then visualized using the iTOLL web server (Letunic and Bork, 2024).

### 2.9 Model of bipartite interaction

A basic version of the figure was initially generated using an AI-assisted tool (ChatGPT, OpenAI), and it was adapted and refined to accurately represent the data and findings.

## 3 Results

### 3.1 *Fusarium verticillioides* degrades epidermis and root hairs

**Figure 1:**
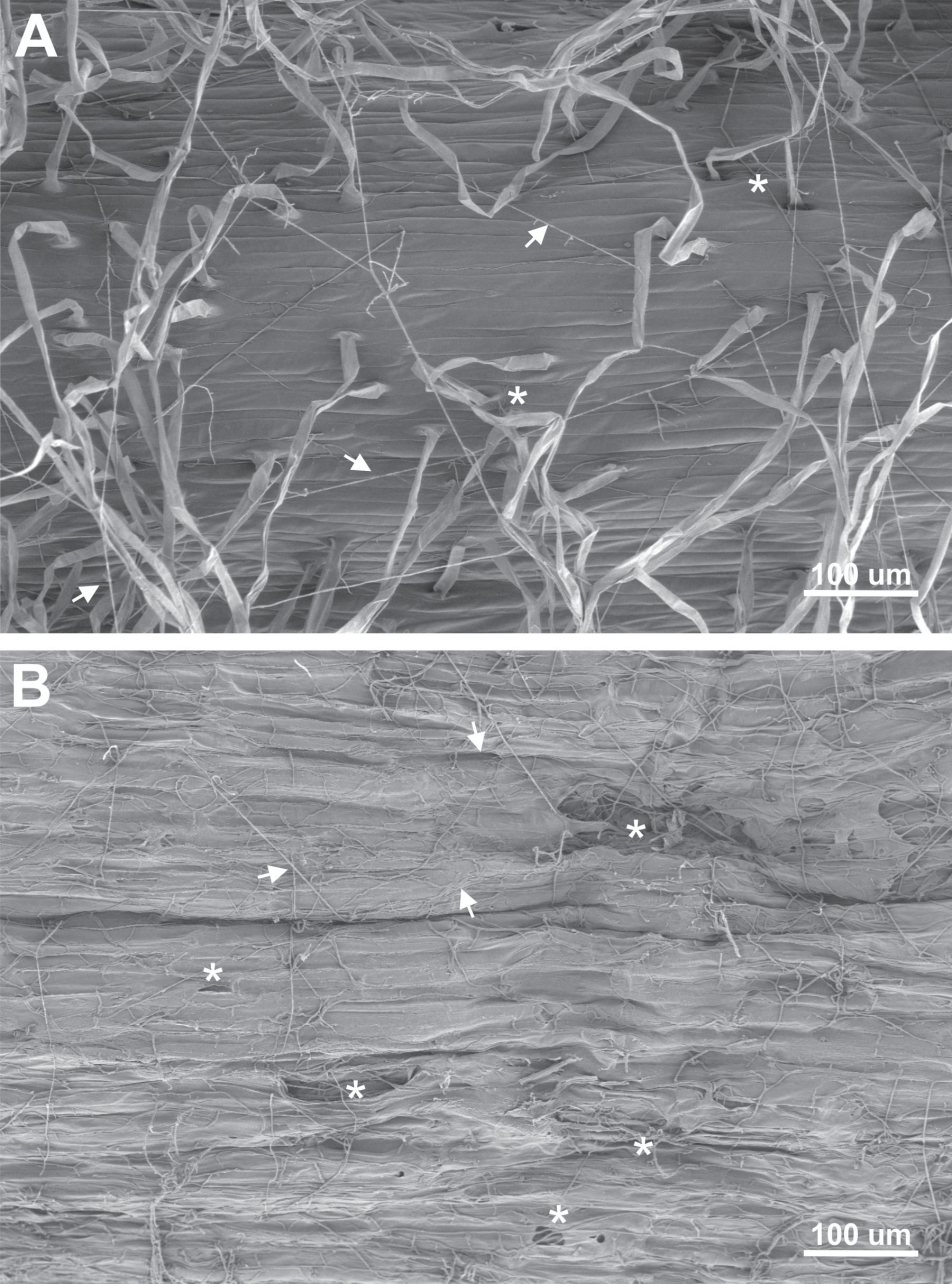
Appearance of the maize primary root epidermis seven days after seed infection by *Fusarium verticillioides* DA42 strain. Two regions with contrasting appearances were analyzed by SEM, **(A)** a region without visible necrosis, and **(B)** a necrotic region. Root hairs with collapsed bases or holes are marked with asterisks (*), and fungal growth on the epidermis surface is indicated by arrowheads.

To determine whether the mechanism of root infection involves secretome-mediated degradation of the epidermis and root hair cell walls, seeds were infected with *Fv* DA42 strain. The primary roots, including apparently healthy tissue (without visible necrosis) and necrotic tissue from 7-day-old seedlings, were observed by SEM (**Figure 1**). In the root zone without visible necrosis, *F. verticillioides* hyphae grew over the surface of the epidermis and among the root hairs. Root hairs were abundant, and many of them in the basal zone was collapsed and sunken, suggesting a loss of cell wall firmness (**Figure 1A**). In contrast, in the necrotic root tissues, there were no roots hairs, and the epidermal surface exhibited extensive degradation, even showing holes (**Figure 1B**). *Fv* hyphae grew profusely over the damaged tissue. The extensive degradation of the root epidermis and root hairs, along with the loss of cell wall integrity, is consistent with the action of cell wall-degrading enzymes present in the fungal secretome, supporting the hypothesis that secretome-mediated degradation is a key mechanism in the *Fv* root infection process.

### 3.2 Fusarium verticillioides genome assembly

Previous evidence indicates that *Fv* secretes enzymes that play a crucial role during the root infection process. Therefore, it is essential to characterize the complete repertoire of *Fv* secreted proteins (the secretome). The *Fv* genome was assembled *de novo* from paired-end Illumina reads. The final assembly comprises 175 contigs, with the largest contig measuring 2.911 Mb, yielding a total assembly size of 42.27 Mb, an N50 of 1.244 Mb, and an N90 of 0.431 Mb. Predicted gene lengths ranged from 201 bp to 22,734 bp, with a mean gene length of 1,492 bp, and an overall GC content of 48.51%. Mapping to the reference genome was performed. Raw paired-end reads were aligned to the *F. verticillioides* reference gap-less genome (GCA_027571605.1) using Bowtie2 (v2.5.3). Mapping quality was evaluated with Qualimap BamQC. Of 37,630,594 total reads, 36,178,219 (96.14%) mapped to the reference, leaving 452,375 reads (3.86%) unmapped. Properly paired reads accounted for 36,178,219 (96.14%), while singleton mappings represented 307,027 reads (0.82%). No duplicates were flagged in the BAM file, but an estimated 13,628,671 reads were marked as duplicates, yielding a duplication rate of 63.22%. The mean read length was 117 bp, and 1.8% of read pairs overlapped. The mean mapping quality score was 40.21. Coverage analysis showed an average depth of 100.93× (SD = 55.68), with per‐chromosome mean coverages ranging from 95.68× (chr11: CM000588) to 102.92× (chr3: CM000580), and a GC content of 48.83%. Insert sizes were broadly distributed (median = 486 bp; 1st–3rd quartiles = 419–567 bp), and the overall error rate (mismatches plus indels) was 0.53%, comprising 16,648,075 mismatches, 770,398 insertions (2.02% of reads), and 352,021 deletions (0.8% of reads). This assembly quality provides a robust foundation for downstream annotation and functional analyses.

### 3.3 Secretome prediction

**Figure 2:**
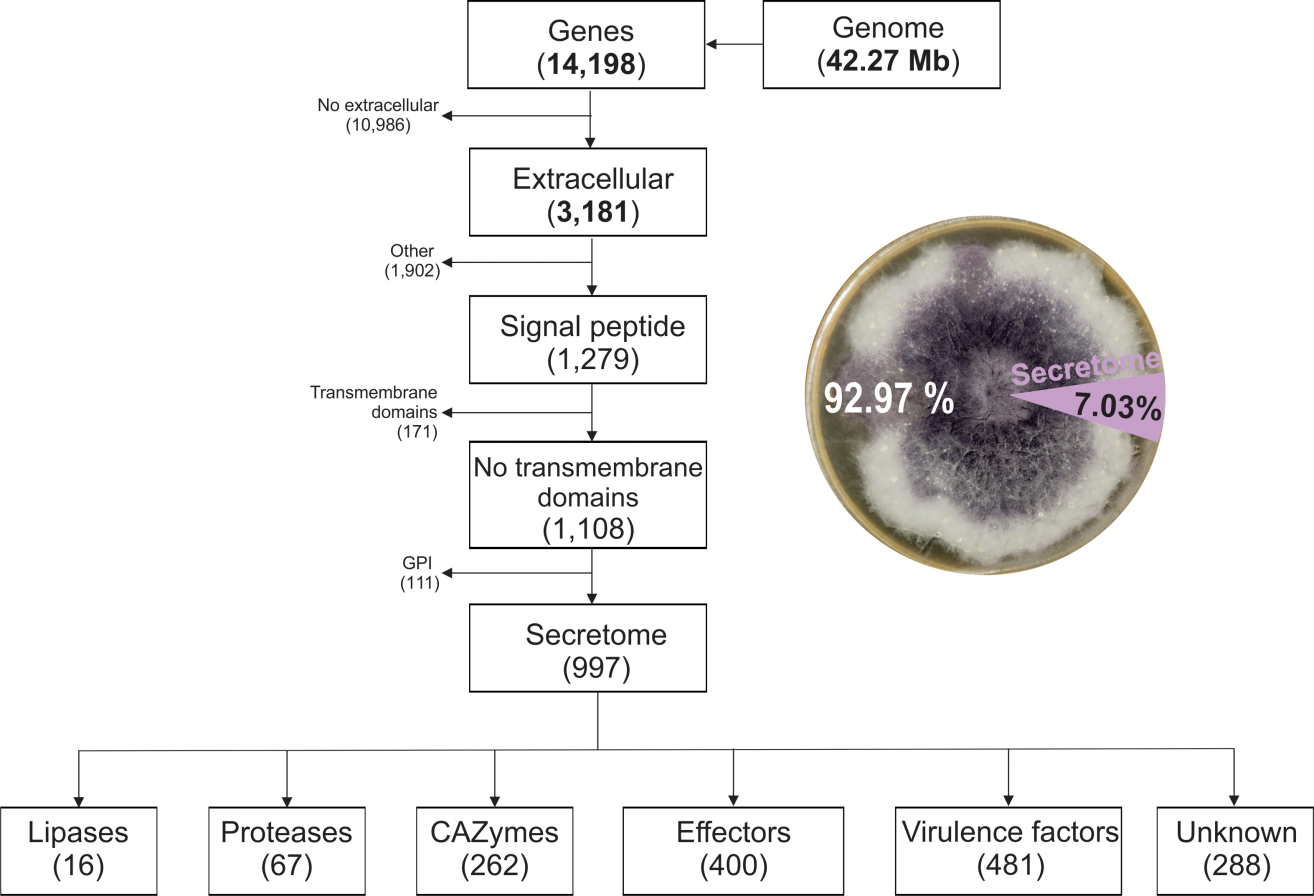
Pipeline for predicting the putative secreted proteins (secretome) of *Fusarium verticillioides* DA42 strain and the distribution of the secretome protein groups.

To identify secreted proteins potentially involved in root cell wall degradation and pathogenesis an *in silico* prediction of the secretome was performed (**Figure 2**). A total of 14,198 protein-coding genes were predicted from the *F. verticillioides* genome. The secretome genes and proteins functionally annotated in the eggNOG-mapper database identified 3,181 extracellular proteins; 1,279 proteins with signal peptide; 1,108 without transmembrane domains, and 111 proteins with glycosylphosphatidylinositol (GPI) anchors.

Out of the 14,198 proteins, 997 were predicted as classical secreted proteins, representing 7.03% of the protein-coding genes (**Supplementary Table 1**). The secretome of *Fv* revealed 16 lipases (1.6%), 62 proteases (6.7%), and 288 unknow or uncharacterized proteins (28.88%). The analyzed subset includes 262 CAZymes, 400 effectors, and 481 virulence factors. The use of diverse databases for functional prediction resulted in redundant function or annotation. In general, 26% of the secreted proteins corresponded to unclassified metabolism, with three major groups related to carbohydrate and glycan metabolism (31%), 11% to amino acid metabolism, and 4% related to lipid metabolism, as identified by BlastKoala (Kanehisa et al., 2016). Smaller proportions were associated with genetic information processing (4%), protein families related to metabolism (6%), and other minor categories (<1%). This distribution highlights the abundance of carbohydrate and amino acid metabolism related-proteins in the secretome, suggesting their relevance in host-pathogen interactions and cell wall degradation mechanism mediated by the secretome.

### 3.4 Genomic distribution and functional annotation of the *Fusarium verticillioides* secretome

**Figure 3:**
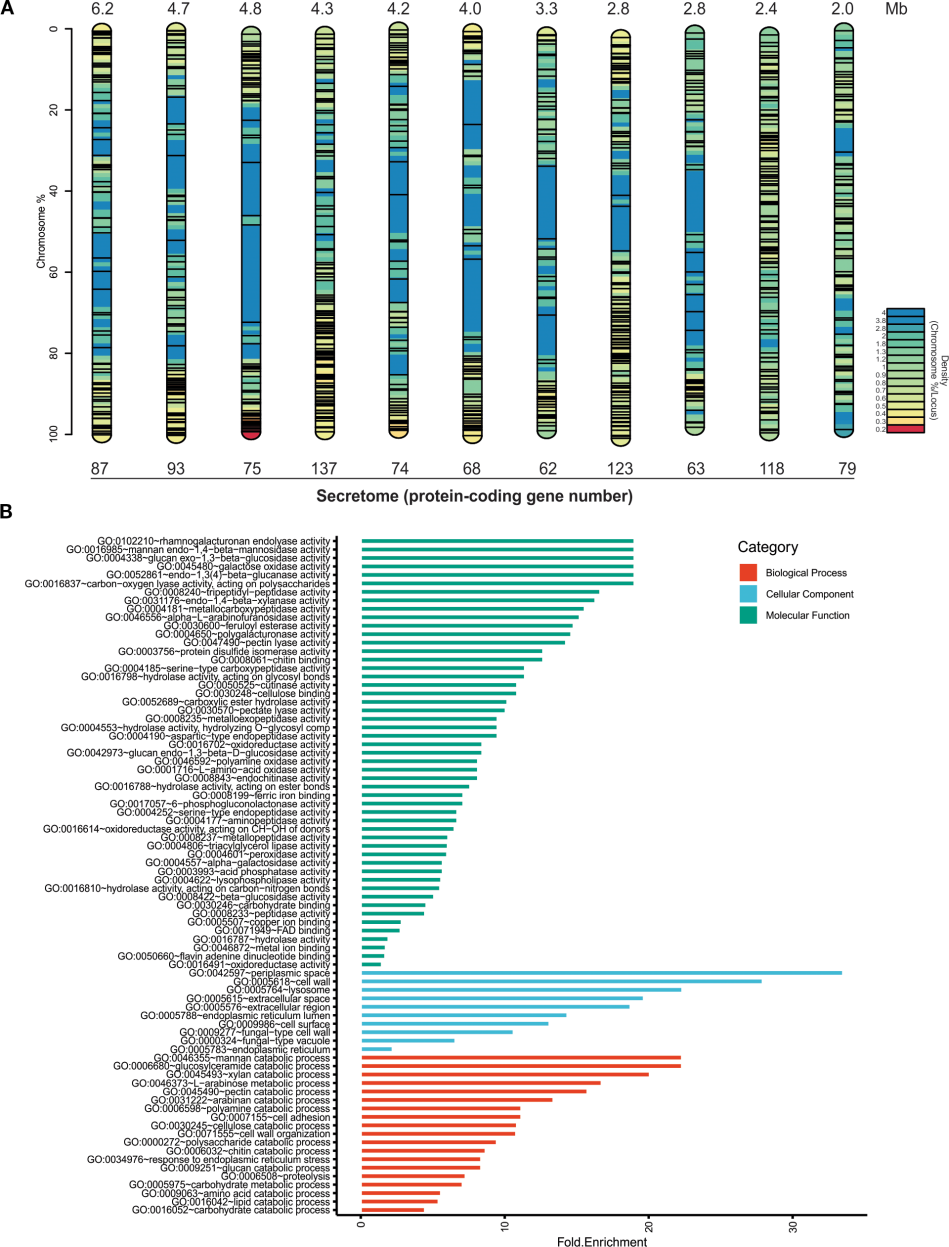
*In silico* analysis of the putative secreted proteins (secretome) of *Fusarium verticillioides* DA42 strain. **(A)** Chromosomal distribution of the secretome (not to scale). The color map represents gene density, and the black lines indicate the localization of genes encoding secreted proteins. **(B)** Gene Ontology (GO) analysis of the filtered secretome. The bar plot shows the counts of the GO terms detected in the analysis for all examined sequences. Green bars represent molecular functions, blue bars represent cellular functions, and red bars represent biological processes.

The chromosomal distribution of secretome genes varied widely across chromosomes (**Figure 3**), with the highest number found on chromosomes 4 (137 genes) and 8 (123 genes). In contrast, the lowest number of protein-coding genes were observed on chromosomes 7 (62 genes) and 9 (63 genes). However, the secretome gene density was higher on chromosome 10, with 49.2 genes/Mb, while chromosome 1 had the lowest density with 14 genes/Mb. Eleven genes, including FVEG_14091 (ricin B lectin), FVEG_13989 (gluconolactonase), and FVEG_14136 (alpha-glucosidase), were not located on any chromosome, but were instead found in the scaffolds NW_017387866.1, NW_017387870.1, and NW_017387871.1 of the reference genome. Gene ontology (GO) terms for the secretome proteins were identified by functional category (**Figure 3B**). The molecular function annotation term (green bars) for the secretome revealed the presence of enzymatic activities associated with plant cell wall degradation. The analysis of the most significantly enriched terms identified proteins involved in rhamnogalacturonan endolyase activity (GO:0102210), mannan endo-1,4-β-mannosidase activity (GO:0016985), and glucan exo-1,3-β-glucosidase activity (GO:0004338). Additional activities included galactose oxidase (GO:0045480), endo-1,3(4)-β-glucanase (GO:0052861), and carbon-oxygen lyase acting on polysaccharides (GO:0016837). Furthermore, proteolytic activities such as tripeptidyl-peptidase (GO:0008240) and metallocarboxypeptidase (GO:0004181) were also detected, indicating potential roles in protein degradation during host colonization. The presence of endo-1,4-β-xylanase (GO:0031176) and α-L-arabinofuranosidase (GO:0046556), along with other relevant and abundant activities for root cell wall degradation were identified, such as pectin lyase activity (GO:0047490) and feruloyl esterase activity (GO:0030600).

The cellular component prominent GO terms (blue bars) include apoplastic space (GO:0042597), extracellular region (GO:0005615 and GO:0005576), and cell‐wall, which are consistent with the secretory nature of these proteins. The biological processes GO terms (red bars) include catabolic processes to degrade the principal component of cell wall, including polysaccharides (simple and complex) (GO:0000272, GO:0046355, GO:0045493, and GO:0046373), proteins (GO:0009063 and GO:0006508), and lipids (GO:0016042). In general, the extensive diversity and genomic distribution of these genes, along with their functional profiles, suggest that many secreted proteins participate in host interactions and extracellular enzymatic activities characteristic of virulence factors and effectors in pathogens.

### 3.5 Phylogenetic clustering of CAZymes and proteases in the *Fusarium verticillioides* secretome

**Figure 4:**
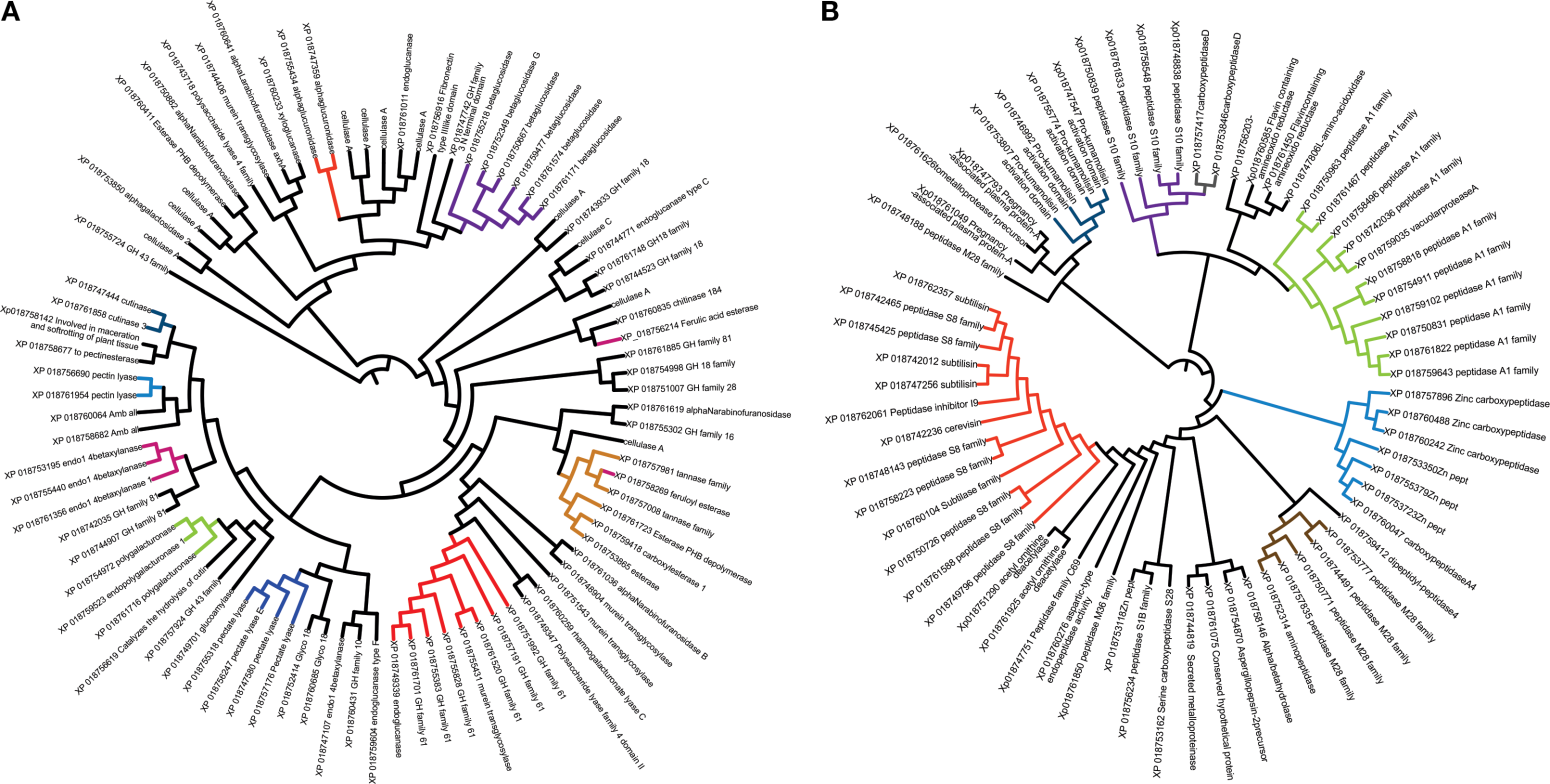
Phylogenetic analysis using the UPGMA method to cluster proteins by their evolutionary similarity of two secreted protein groups of *Fusarium verticillioides* DA42 strain. **(A)** CAZymes and **(B)** proteases. Proteins with close sequences are clustered and shown in the same color.

A phylogenetic analysis was performed based on protein evolutionary similarity within the two more abundant functional groups of the *Fv* DA42 secretome: CAZymes (carbohydrate-active enzymes) and proteases (**Figure 4**). The phylogenetic relationships among various CAZymes involved in the degradation, modification, or biosynthesis of carbohydrates are evident (**Figure 4A**). Branches group proteins with similar sequences, indicating families that are functionally or evolutionarily related. Several well-defined clades (e.g., highlighted in red, orange, and purple) suggest that certain CAZymes share conserved domains or common activities, such as glucosidases, cellulases, chitinases, and glucosyl hydrolases. Additionally, a phylogenetic analysis of secreted proteases, enzymes potentially involved in degrading host proteins and contributing to virulence, reveals clustering by sequence similarity (**Figure 4B**). Colored clusters reflect specific protease families, such as serine proteases or metalloproteases, and the presence of multiple distinct clades underscores the functional diversification among the secreted proteases. Overall, the phylogenetic analysis highlights the structural and functional differences and similarities between the main secreted protein groups in *Fv* DA42 strain. Highly conserved proteins within each group suggest key roles in host interaction, either by degrading plant cell wall components or by interfering with host defenses through proteolytic activity.

### 3.6 Protein-protein interaction of key enzymes and effectors identified through STRING

**Figure 5:**
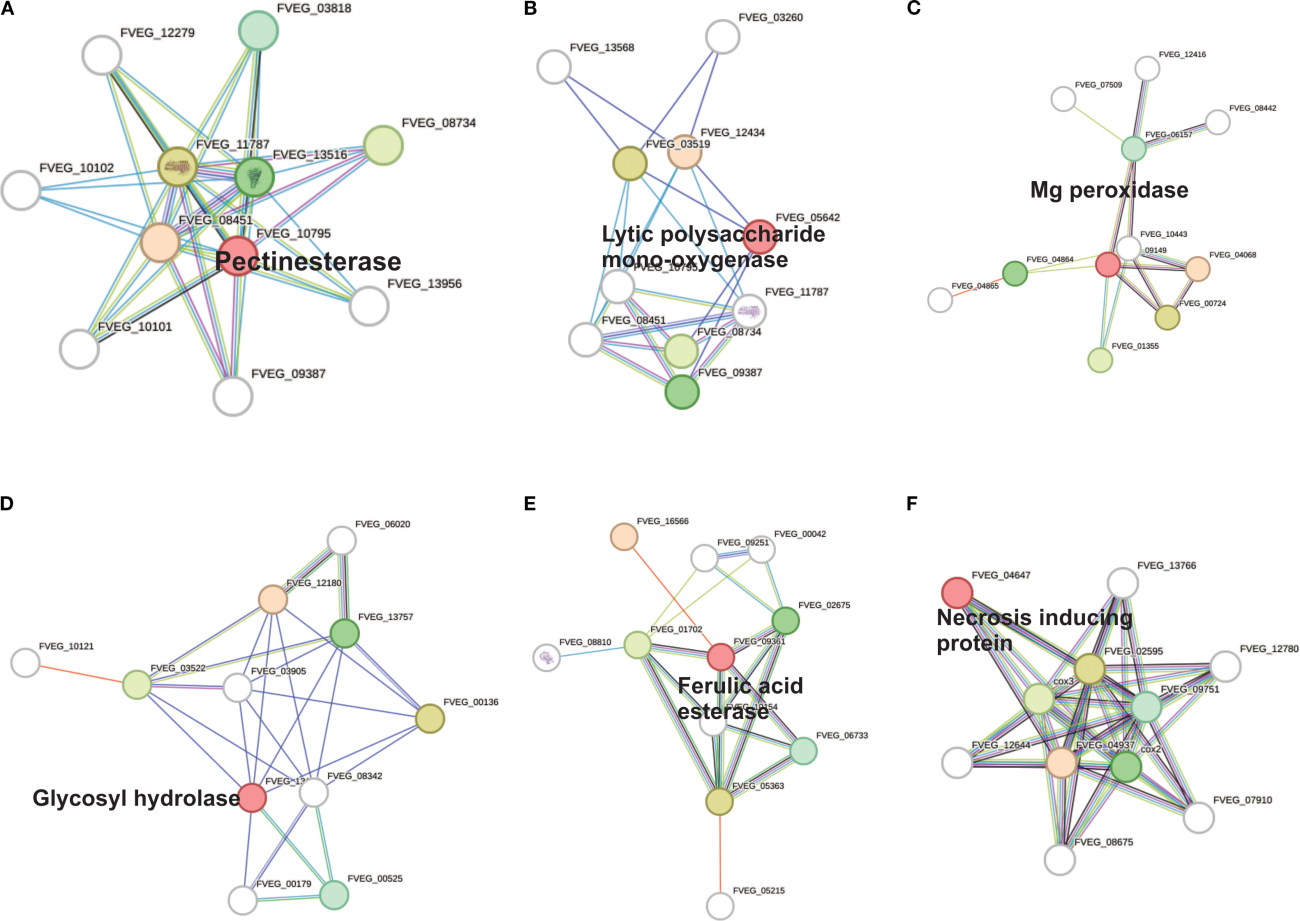
Protein-protein interactions in the effector proteins identified by their high identity (>80%) with the PHI-base database. Confidence scores were extracted from STRINGdb. **(A)** Pectinesterase (FVEG_10795), **(B)** Lytic polysaccharide mono-oxygenase (FVEG_05642), **(C)** Mg peroxidase (FVEG_09149), **(D)** Glycosyl hydrolase (FVEG_13183), **(E)** Feruloyl esterase (FVEG_09361), and **(F)** Necrosis inducing protein (FVEG_04647).

Based on a global STRING analysis of 14 selected genes related to cell wall degradation and infection process and their high identity in the PHI-base database. (**Supplementary Table 2**), several genes (FVEG_03246, FVEG_10795, and FVEG_05849) with high functional connectivity (scores > 0.7) were identified, exhibiting very strong interactions related to cell cycle, plant cell wall degradation, and putative adhesion functions, respectively. We also detected polysaccharide degradation networks involving multiple genes, such as FVEG_10795 (putative pectinesterase), FVEG_09361 (putative ferulic acid esterase), FVEG_05642 (putative chitin binding protein), and FVEG_09702 (putative pectate lyase), suggesting their coordinated role in modifying the plant cell wall, potentially during fungal infection. In parallel, FVEG_09149 (Mg peroxidase) and FVEG_04647 (putative necrosis inducing protein) appear to be involved in cell wall degradation through the depolymerization of lignin and necrosis processes. Based on their high STRING score analysis and relevant literature, a subset of genes was selected for visualization (**Figure 5**), each linked to a specific enzyme or effector: FVEG_09361 (feruloyl esterase, EC 3.1.1.73), FVEG_10795 (pectinesterase, EC 3.1.1.11), FVEG_13183 (cell wall glycosyl hydrolase), FVEG_05642 (chitin-binding type-4 domain protein), FVEG_09149 (peroxidase, EC 1.11.1), and FVEG_04647 (necrosis-inducing protein). This analysis confirms the roles for these proteins in plant cell wall degradation (pectinesterases, polygalacturonases, and esterases), as virulence effectors (necrosis inducers or chitin-binding camouflage proteins), and in oxidative responses (peroxidases). Specifically, FVEG_10795 (pectinesterase) catalyzes the demethylation of pectin, a prerequisite for its breakdown. It interacts most strongly with FVEG_08451 (endo-polygalacturonase; score 0.933). Together, these enzymes likely act sequentially; FVEG_10795 demethylates pectin, and FVEG_08451 cleaves it into monomers. FVEG_09361 (feruloyl esterase) contributes to the breakdown of ferulic acid cross-links in the plant cell wall. Its main partner is FVEG_16566 (score 0.372), an AB hydrolase-1 domain protein that may participate in related hydrolytic pathways. FVEG_13183 (cell wall glycosyl hydrolase) probably targets glucans or other complex polysaccharides. Its principal interactor is FVEG_12180 (score 0.366), an SGNH-type esterase involved in polysaccharide ester modification, suggesting a shared depolymerization pathway. FVEG_05642 (chitin-binding type-4 domain protein) acts as a fungal effector, masking chitin from plant defenses. Its main interactor is FVEG_12434 (pectate lyase; score 0.224); although the score is low, this suggests potential collaborative mechanisms for attacking chitin and pectate. FVEG_09149 (peroxidase) is involved in oxidative processes, likely related to defense or stress adaptations, and interacts primarily with FVEG_04068 (ribonuclease H2 subunit B; score 0.723), indicating a possible co-expression or joint participation in damage-response or oxidative-stress pathways. FVEG_04647 (necrosis-inducing protein) is a putative fungal effector that triggers host cell death. Its main interaction is with FVEG_04937 (cytochrome c oxidase subunit; score 0.490), implying a link to mitochondrial processes that may be tied to necrosis or stress signaling in the host.

### 3.7 Phylogenetic relation of draft genome analysis of *Fusarium verticillioides* DA42 strain

**Figure 6:**
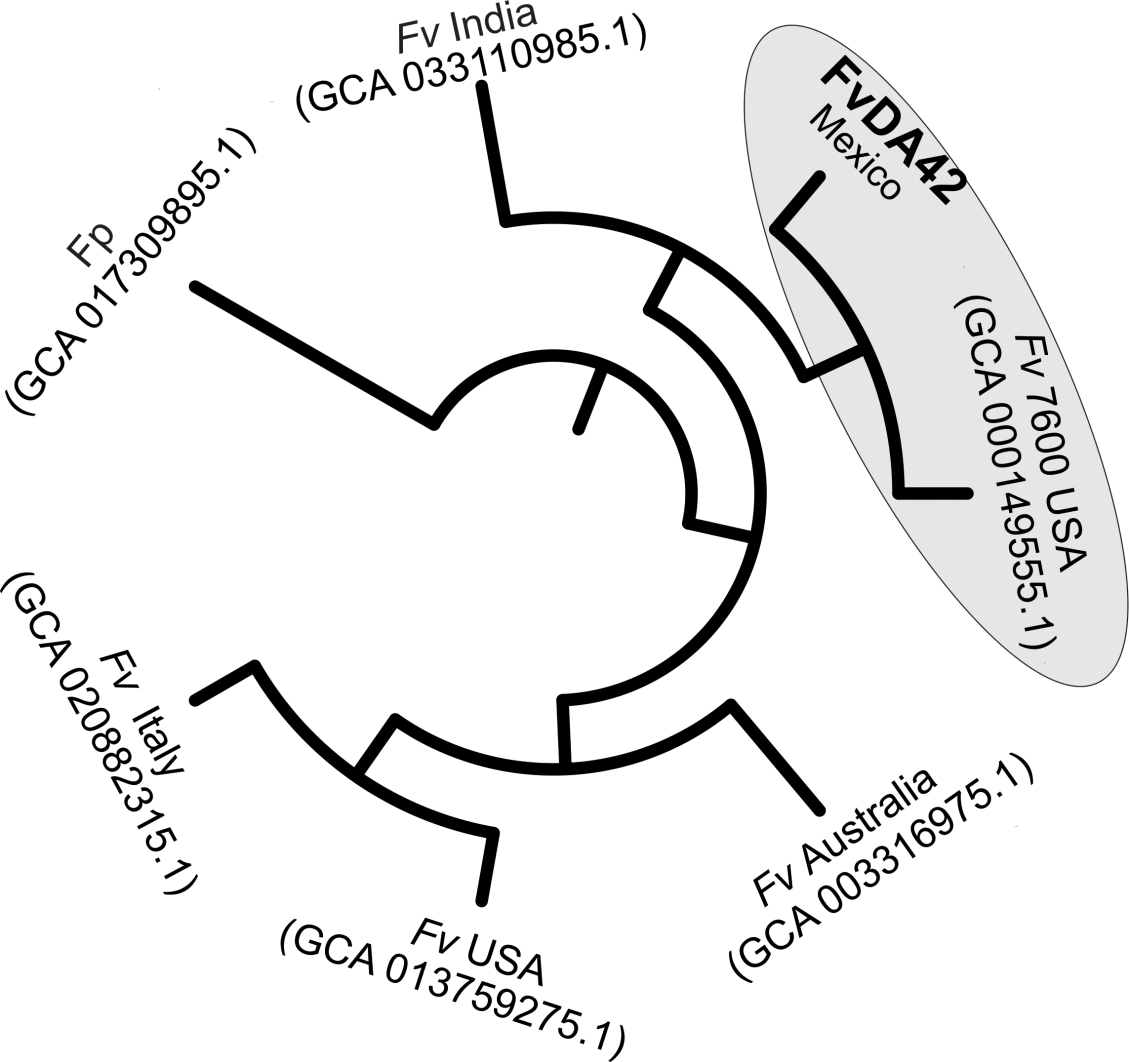
Whole-genome phylogenetic analysis of *Fv* DA42 strain. Fv, *Fusarium verticillioides* and Fp, *Fusarium proliferatum*.

The phylogenetic analysis of the genomes included strains isolated from the United States, Australia, India, and Mexico. The phylogenetic tree (**Figure 6**) showed that the *Fv* DA42 strain genome from Mexico clustered with the *Fv* 7600 reference genome (GCA000149555.1) isolate in USA and an Indian strain (GCA033110985.1), however, *Fv* DA42 strain genome was more closely related to the reference strain than to the Indian strain. In a different tree branch another *Fv* isolate from USA (GCA013759275.1) groups with the isolate from Italy (GCA020882315.1) and both are more distant to the isolate from Australia (GCA003316975.1). The phylogenetic analysis supports a close evolutionary relationship between the DA42 strain and the reference genome 7600 strain, while revealing partial genetic distances among isolates from different geographic regions of origin.

## 4 Discussion

The extended agricultural life cycle of maize (6–8 months) exacerbates the challenges associated with *Fusarium* infection. From the vulnerable seedling stage to the critical phases of vegetative and reproductive growth, the potential for *Fusarium* to disrupt and delay normal development of maize remains a persistent concern for both farmers and researchers (Bryla et al., 2022). The fungus’s ability to penetrate various plant tissues, including roots, stalks, and seeds, underscores the need for a comprehensive understanding of the mechanisms that facilitate its entry and subsequent colonization within the host (Duncan and Howard, 2010; Gai et al., 2018; Thompson and Raizada, 2018; Roman et al., 2020). Wu et al. (2011) and Quiroz-Figueroa et al. (2023) describe the early manifestations of *Fusarium* infection, which include yellow-brown discoloration and the subsequent formation of necrotic spots on the roots. These symptoms not only serve as visible indicators of infection but also suggests a complex interaction between the pathogen and the host plant at the cellular level. The collapse of root hairs further emphasizes the severity of this interaction, suggesting the active degradation of cell walls in the epidermal cells during a necrotrophic phase (**Figure 1**). Although, the SEM observations were made at 7 days post infection and thus correspond to an advanced stage of root necrosis, it is highly likely that the degradative enzymatic machinery and signaling are strongly activated several days earlier (Quiroz-Figueroa et al., 2023). In the current study, the *de novo* high-quality genome assembly of *Fv* DA42 strain allowed for downstream annotation and functional analyses. The DA42 strain showed a closest phylogenetic similarity to the genome reference *Fv* 7600 (GCA_000149555.1) reported by Ma et al. (2010) and the improved gapless genome in Yao et al. (2023), than to the BIONCL4 (GCA_033110985.1) strain (Navale et al., 2022). This indicates a close evolutionary relationship (**Figure 6**) and potentially similar phenotypic behavior. Similar to the findings of Navale et al. (2022), the genome assembly from Italy (GCA020882315.1) showed greater distance from the DA42 strain. The separation of strains from Mexico (*Fv*DA42) and U.S. (*Fv* USA), and as well as the convergence of Mexican (*Fv*DA42), U.S. (*Fv*7600 USA) and Indian (*Fv* India) strains into different clades or shared clades respectively, may reflect historical gene flow associated with the global movement and exchange of commercial maize germplasm, local adaptation and long-term cultivation of genetically distinct maize varieties in each region could have driven the divergence of associated *Fv* populations. Although our coverage analysis was over 100.9X and the mapping to the first version reference genome was 96.14%, these differences could be related either to sequence technology or intra-species variability. Therefore, the genome of *Fv* DA42 strain, which clusters closest to the reference *Fv* 7600 strain, may offer valuable insights into infection mechanisms within the *Fusarium* genus, and represents a promising candidate for comparative future pathogenicity assays.

Despite the importance of the *Fv* secretome, a few studies have been conducted to identify secreted proteins. The results of this study reinforce the key role of the secretome for *Fv* fungal infection and colonization processes in maize roots. According to our characterization, approximately 7% of the *Fv* DA42 proteome corresponds to secreted proteins via classical pathways, primarily consisting of CAZymes, proteases, effectors, and virulence factors, which play important roles in cell wall degradation and overcoming maize defenses. In accordance with the relevance of the integrity of the host cell wall, recent finding on the maize glycosyltransferase (ZmXYXT2) showed that reinforcement of the cell walls restricted *F. verticillioides* infection, whereas thinner cell walls in the zmxyxt2 mutant facilitated the colonization and fumonisin accumulation (Xu et al., 2025). Studies on plant pathogens emphasize the significance of secretomes in plant–fungus interactions, the proportion of these secreted proteins ranges from 4% to 14% for necrotrophs compared to biotrophs (Lowe and Howlett, 2012). Ravalason et al. (2012) reported the first proteome of *Fv* 7600, identifying a total of 166 secreted proteins, which represented only 1.17% of the predicted proteome, a relatively low proportion of the total protein-coding genes. This limited detection may be attributed to the proteomic technique used at the time, which likely had lower sensitivity and coverage compared to other advanced or complementary techniques available today. A few years later, Navale et al. (2022) performed whole-genome sequencing of *F. verticillioides* BIONCL4 strain isolated from maize grains using Illumina technology. They predicted a total of 15,053 protein-coding-genes, of which 2,058 were secreted proteins and 676 were associated with the classical secretory system. These represent 13.6% and 4.49% of the total of secreted proteins and classical secretory pathways, respectively. The predicted secretome of *F. graminearum*, is approximately 4.11% (Brown et al., 2012). In the *in silico* secretome of the palm dieback-causing agent *F. oxysporum* f. sp. albedinis, 1,464 out of 16,887 genes (8.6%) were predicted as secreted proteins (Rafiqi et al., 2022). A recent study in *F. graminearum* provides the first proteome secreted into the apoplast of wheat coleoptiles, identifying the metalloprotease effector Fg28, which induces cell death and reactive oxygen species at the first day of infection (Li et al., 2025). Similarly, in *F. oxysporum*, a novel secreted cysteine-rich protein, FolSCP1, was identified as a virulence factor that promotes infection by binding and inhibiting PR-5, a positive regulator of tomato immunity (Qian et al., 2025). In *F. verticillioides*, a RNA-seq analysis revealed that FvLcp1, a secreted LysM protein required for fumonisin production, which accumulates in appressoria and contributes to host recognition, suppression of host cell death, and promotion of fumonisin biosynthesis during maize kernel colonization (Zhang et al., 2022). These studies highlight the role of secreted proteins in *Fusarium* host interactions. In particular, these emphasize the biological relevance of the *Fv* secretome and the need for further studies to better understand its role in the molecular mechanisms of maize–*Fusarium* interactions.

The GO analysis of the DA42 strain secretome (**Figure 3B**) revealed enrichment in enzymatic activities such as endogalacturonases, exoglucosidases, and feruloyl esterases, which are essential for breaking down pectin, hemicellulose, and lignin. These biological processes were classified under the carbohydrate catabolism. Also, processes related to proteolysis, cell adhesion and cell wall organization were identified. These results are consistent with secretome analyses of necrotrophic fungi, such as *Neofusicoccum parvum* (Nazar Pour et al., 2022) and *Botrytis cinerea* (Nazar Pour et al., 2022), where CAZymes, including pectinesterases, endopolygalacturonases, and proteases, are involved in host protein degradation.

Similarly, several effectors were identified in *Fv* DA42, including peroxidases and necrosis-inducing proteins, which are comparable to those known to induce host necrosis or suppress host immunity in other fungal pathogens (Cheung et al., 2020; Gupta et al., 2022; Bouqellah et al., 2023). A meta-analysis conducted by Jia et al. (2023) on 150 phytopathogenic fungi revealed that secretomes vary with fungal lifestyle, with necrotrophs tending to secrete a wide range of CAZymes targeting pectin and xylan. One study showed that the predicted secretome of *F. graminearum*, which includes 574 proteins, contains phytotoxic enzymes and effectors, many of which are transcriptionally upregulated during infection (Brown et al., 2012). The interaction between CAZymes and proteases (**Figure 4**) and the connected nodes in the protein–protein interaction network (**Figure 5**) for FVEG_10795, FVEG_09361, and FVEG_09149 reinforce the hypothesis of a coordinated mechanism for fungal attack. This could involve sequential pectin demethylation, enzymatic breakdown, and oxidative stress, forming a tripartite virulence strategy. The collapse of root hairs and degradation of the cell wall epidermis observed by SEM microscopy (**Figure 1**) confirms that enzymatic attack is effective during the early stages of infection, as previously hypothesized by Quiroz-Figueroa et al. (2023).

**Figure 7:**
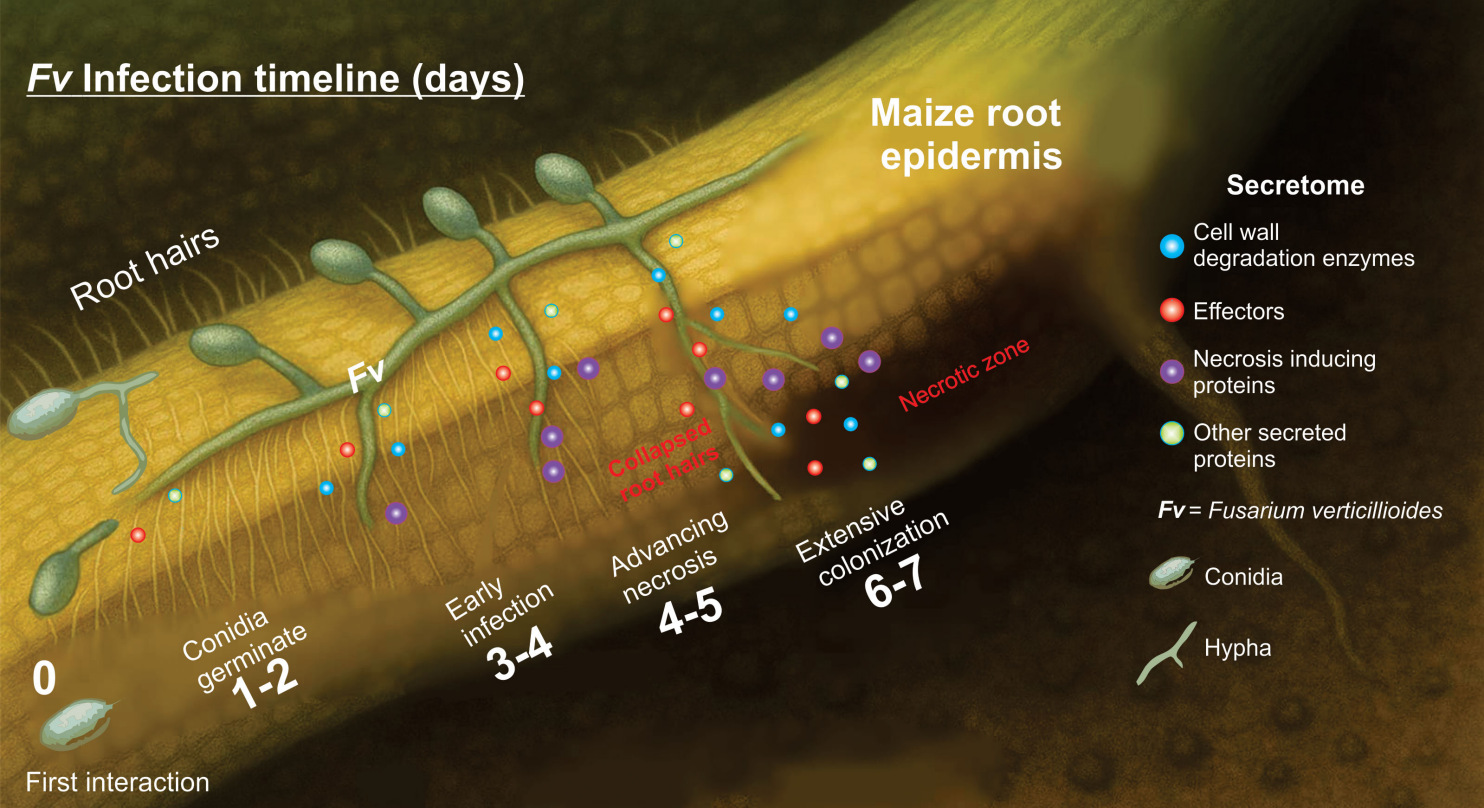
Proposed model of *Fusarium verticillioides* infection in maize roots from 0 to 7-day post-inoculation. The diagram illustrates five progressive stages of *Fv* infection: Day 0: The first interaction: Healthy maize root with abundant root hairs and no visible root damage. Conidia are deposited on the epidermal surface. Days 1–2: Conidia germinate, forming germ tubes and initiating surface contact with epidermal cells. Root hairs remain intact. The secretome signaling is activated. Day 3-4: Early infection. The hyphae increase their enzyme secretion, and signs of color change appear in the root epidermis. Root hairs are still present, but they are beginning to collapse at the base. Days 4–5: Advancing necrosis. Epidermal cells are damage, root hairs disappear in infected zones, and the hyphal invasion expands. Days 6–7: Extensive colonization. Fungal hyphae are well-established within inner tissues, and necrotic lesions dominate the infection site. This model proposes a dynamic interaction between *Fv* and the roots of susceptible maize, highlighting the progressive loss of root integrity and root hairs as the infection advance. Root hair collapse is notably associated with tissue necrosis and fungal invasion. A similar model cannot be ruled out for other necrotrophic fungi.

This research integrates scan electronic microscopy, genome sequencing, *in silico* secretome analysis, and protein–protein interaction analysis to decipher the infection strategy of *F. verticillioides* in maize roots. Previous studies have addressed the general pathogenicity of this species, primarily focusing on ear and stalk infections. Our study provides the first integrated correlation between *in silico* secretome composition and SEM observations of early-stage root tissue degradation, including root hairs collapse and breakage of epidermal cell walls. To our knowledge, this is the first report that combines multi-layered genomic and proteomic predictions with structural (SEM) evidence of infection in maize roots by *Fv*. This integrative approach opens new aspects to understand the molecular arsenal underlying fungal root pathogenesis and to identify specific secreted proteins as promising targets for resistance breeding and antifungal strategies, thereby contributing with valuable tools for sustainable crop protection. In summary, the secretome of *F. verticillioides* plays a central role in colonizing susceptible maize roots, making evident the enrichment of enzymes related to cell wall degradation (carbohydrate polymers, glucans and proteins), effectors, and virulence. Although *Fv* has been described as a hemibiotroph, our OMIC data and SEM observations (in this and our previous report) suggest a predominantly necrotrophic lifestyle when interacting with susceptible genotypes during the early infection stages, likely driven by the early and sustained activation of cell wall–degrading enzymes and necrosis-inducing factors through still unknown mechanisms (**Figure 7**).

## 5 Conclusion

This study provides a multidimensional analysis of *Fv* DA42, combining SEM, genome assembly, *in silico* secretome prediction, interaction network analyses, and phylogenetic. We identified that approximately 7% of the strain’s proteome consists of secreted proteins, which are rich in CAZymes, proteases, peroxidases, effectors and necrosis-inducing proteins. Through SEM, we detected early-stage damage, including the collapse of root hairs and degradation of root epidermal cell walls, directly linking molecular predictions with structural evidence. Significant interaction, such as the sequential action of FVEG_10795 (pectinesterase) and FVEG_08451 (polygalacturonase), support a model of coordinated enzymatic machinery that deconstructs pectin in a stepwise manner during the initial colonization. To our knowledge, this is the first integrated report that combines whole-genome secretome annotation, protein–protein interaction modeling, and root tissue-level SEM observations to elucidate the molecular mechanisms of *F. verticillioides* root infection. The specific secreted proteins identified emerge as promising targets for future genetic resistance breeding or/and antifungal control strategies. Our work thus lays a solid foundation for sustainable prevention methods against maize root rot and enhances the molecular understanding of *Fusarium*–maize interactions.

## Data Availability

The datasets presented in this study can be found online the BioProject PRJNA1294327.
