# Supplementary material for: Unveiling the molecular arsenal: genome sequencing and in silico secretome analysis of Fusarium verticillioides provide insights into maize root rot pathogenesis
Source: Front Plant Sci. 2025 Sep 30;16:1672761. doi: 10.3389/fpls.2025.1672761 (PMC12520017; doi:10.3389/fpls.2025.1672761)
Supplement: Supplementary file 1 [file Table1.docx]

Supplementary Material

for

Unveiling the molecular arsenal: Genome sequencing and *in silico* secretome analysis of *Fusarium verticillioides* provide insights into maize root rot pathogenesis

**Supplementary Table 1.** Set of secreted proteins obtained in *Fusarium verticillioides* (DA42 strain) annotated according to secretome pipeline

| **contig** | **chromosome** | **gene name** | **Refseq protein** | **Product** |
| --- | --- | --- | --- | --- |
| contig00001.g2 | NC_031675.1 | FVEG_00003 | XP_018741975.1 | Uncharacterized protein |
| contig00001.g14 | NC_031675.1 | FVEG_00014 | XP_018741988.1 | GEgh 16 protein |
| contig00001.g26 | NC_031675.1 | FVEG_00027 | XP_018742012.1 | Subtilisin |
| contig00001.g46 | NC_031675.1 | FVEG_00044 | XP_018742035.1 | glucan endo-1,3-beta-D-glucosidase (EC 3.2.1.39) |
| contig00001.g47 | NC_031675.1 | FVEG_00045 | XP_018742036.1 | Peptidase A1 domain-containing protein |
| contig00001.g63 | NC_031675.1 | FVEG_00062 | XP_018742052.1 | Multiprotein-bridging factor 1 |
| contig00001.g64 | NC_031675.1 | FVEG_00063 | XP_018742053.1 | Uncharacterized protein |
| contig00005.g3722 | NC_031675.1 | FVEG_00094 | XP_018742096.1 | Uncharacterized protein |
| contig00001.g99 | NC_031675.1 | FVEG_00097 | XP_018742099.1 | Lysine-specific metallo-endopeptidase domain-containing protein |
| contig00001.g122 | NC_031675.1 | FVEG_00120 | XP_018742128.1 | Glycan binding protein Y3-like domain-containing protein |
| contig00001.g124 | NC_031675.1 | FVEG_00121 | XP_018742130.1 | Lysine-specific metallo-endopeptidase domain-containing protein |
| contig00001.g127 | NC_031675.1 | FVEG_00123 | XP_018742133.1 | Cyanovirin-N domain-containing protein |
| contig00001.g141 | NC_031675.1 | FVEG_00136 | XP_018742148.1 | SGNH hydrolase-type esterase domain-containing protein |
| contig00001.g201 | NC_031675.1 | FVEG_00200 | XP_018742223.1 | GH16 domain-containing protein |
| contig00001.g211 | NC_031675.1 | FVEG_00212 | XP_018742236.1 | Cerevisin |
| contig00001.g224 | NC_031675.1 | FVEG_00226 | XP_018742253.1 | Uncharacterized protein |
| contig00001.g247 | NC_031675.1 | FVEG_00250 | XP_018742281.1 | Transcription factor domain-containing protein |
| contig00001.g258 | NC_031675.1 | FVEG_00262 | XP_018742298.1 | Glucose-methanol-choline oxidoreductase |
| contig00001.g261 | NC_031675.1 | FVEG_00265 | XP_018742303.1 | Uncharacterized protein |
| contig00001.g267 | NC_031675.1 | FVEG_00271 | XP_018742311.1 | Counting factor 60 |
| contig00001.g268 | NC_031675.1 | FVEG_00272 | XP_018742313.1 | SET domain-containing protein |
| contig00001.g269 | NC_031675.1 | FVEG_00273 | XP_018742314.1 | Ecp2 effector protein-like domain-containing protein |
| contig00001.g287 | NC_031675.1 | FVEG_00291 | XP_018742342.1 | Sialidase-1 |
| contig00001.g294 | NC_031675.1 | FVEG_00297 | XP_018742350.1 | Uncharacterized protein |
| contig00001.g295 | NC_031675.1 | FVEG_00298 | XP_018742351.1 | Uncharacterized protein |
| contig00001.g346 | NC_031675.1 | FVEG_00352 | XP_018742438.1 | Glycosyl hydrolase |
| contig00001.g365 | NC_031675.1 | FVEG_00370 | XP_018742465.1 | Subtilisin |
| contig00001.g402 | NC_031675.1 | FVEG_00392 | XP_018742496.1 | MUC1 Extracellular alpha-1,4-glucan glucosidase |
| contig00001.g407 | NC_031675.1 | FVEG_00396 | XP_018742507.1 | Acetylxylan esterase |
| contig00001.g408 | NC_031675.1 | FVEG_00397 | XP_018742509.1 | Acetylxylan esterase 2 |
| contig00001.g544 | NC_031675.1 | FVEG_00537 | XP_018742751.1 | alpha-1,2-Mannosidase (EC 3.2.1.-) |
| contig00001.g562 | NC_031675.1 | FVEG_00554 | XP_018742801.1 | Spore coat protein SP96 |
| contig00001.g635 | NC_031675.1 | FVEG_00632 | XP_018742928.1 | glucan endo-1,3-beta-D-glucosidase (EC 3.2.1.39) |
| contig00001.g646 | NC_031675.1 | FVEG_00645 | XP_018742941.1 | DUF7492 domain-containing protein |
| contig00001.g756 | NC_031675.1 | FVEG_00761 | XP_018743106.1 | Asl1-like glycosyl hydrolase catalytic domain-containing protein |
| contig00001.g836 | NC_031675.1 | FVEG_00846 | XP_018743266.1 | IDI-2 |
| contig00001.g898 | NC_031675.1 | FVEG_00910 | XP_018743393.1 | Terpene synthase (EC 4.2.3.-) |
| contig00001.g1022 | NC_031675.1 | FVEG_01038 | XP_018743616.1 | Chromatin structure-remodeling complex subunit SFH1 |
| contig00001.g1046 | NC_031675.1 | FVEG_01064 | XP_018743654.1 | Uncharacterized protein |
| contig00001.g1084 | NC_031675.1 | FVEG_01111 | XP_018743716.1 | Uncharacterized protein |
| contig00001.g1086 | NC_031675.1 | FVEG_01112 | XP_018743718.1 | Rhamnogalacturonate lyase (EC 4.2.2.23) |
| contig00001.g1208 | NC_031675.1 | FVEG_01240 | XP_018743933.1 | chitinase (EC 3.2.1.14) |
| contig00001.g1210 | NC_031675.1 | FVEG_01243 | XP_018743938.1 | Uncharacterized protein |
| contig00001.g1243 | NC_031675.1 | FVEG_01275 | XP_018743978.1 | Endopolyphosphatase (EC 3.6.1.10) |
| contig00001.g1263 | NC_031675.1 | FVEG_01295 | XP_018744031.1 | Uncharacterized protein |
| contig00001.g1320 | NC_031675.1 | FVEG_01351 | XP_018744157.1 | SGNH hydrolase-type esterase domain-containing protein |
| contig00001.g1330 | NC_031675.1 | FVEG_01361 | XP_018744181.1 | Protein transport protein YOS1 |
| contig00001.g1337 | NC_031675.1 | FVEG_01368 | XP_018744196.1 | AA1-like domain-containing protein |
| contig00001.g1338 | NC_031675.1 | FVEG_01369 | XP_018744197.1 | Fungal lipase-type domain-containing protein |
| contig00032.g13164 | NC_031675.1 | FVEG_01377 | XP_018744207.1 | Protein disulfide-isomerase (EC 5.3.4.1) |
| contig00032.g13126 | NC_031675.1 | FVEG_01414 | XP_018744263.1 | Beta-hexosaminidase (EC 3.2.1.52) |
| contig00032.g13061 | NC_031675.1 | FVEG_01486 | XP_018744406.1 | Murein transglycosylase |
| contig00016.g8870 | NC_031675.1 | FVEG_09484 | XP_018756370.1 | peptidylprolyl isomerase (EC 5.2.1.8) |
| contig00016.g8929 | NC_031675.1 | FVEG_09545 | XP_018756470.1 | EF-hand domain-containing protein |
| contig00016.g8931 | NC_031675.1 | FVEG_09547 | XP_018756472.1 | Uncharacterized protein |
| contig00016.g8991 | NC_031675.1 | FVEG_09611 | XP_018756561.1 | FMN hydroxy acid dehydrogenase domain-containing protein |
| contig00016.g9028 | NC_031675.1 | FVEG_09653 | XP_018756619.1 | Cutinase (EC 3.1.1.74) |
| contig00016.g9069 | NC_031675.1 | FVEG_09694 | XP_018756682.1 | Carboxylic ester hydrolase (EC 3.1.1.-) |
| contig00016.g9076 | NC_031675.1 | FVEG_09702 | XP_018756690.1 | pectin lyase (EC 4.2.2.10) |
| contig00016.g9087 | NC_031675.1 | FVEG_09713 | XP_018756708.1 | LicD/FKTN/FKRP nucleotidyltransferase domain-containing protein |
| contig00016.g9093 | NC_031675.1 | FVEG_09719 | XP_018756716.1 | cellulase (EC 3.2.1.4) |
| contig00030.g12789 | NC_031675.1 | FVEG_09724 | XP_018756722.1 | Histone H2A |
| contig00016.g9130 | NC_031675.1 | FVEG_09758 | XP_018756773.1 | Uncharacterized protein |
| contig00016.g9143 | NC_031675.1 | FVEG_09770 | XP_018756793.1 | Uncharacterized protein |
| contig00016.g9144 | NC_031675.1 | FVEG_09772 | XP_018756795.1 | Beta-glucosidase |
| contig00016.g9151 | NC_031675.1 | FVEG_09778 | XP_018756809.1 | Uncharacterized protein |
| contig00005.g3662 | NC_031675.1 | FVEG_09821 | XP_018756867.1 | protein disulfide-isomerase (EC 5.3.4.1) |
| contig00005.g3659 | NC_031675.1 | FVEG_09824 | XP_018756871.1 | D-amino-acid oxidase |
| contig00005.g3657 | NC_031675.1 | FVEG_09826 | XP_018756873.1 | Oxidoreductase |
| contig00005.g3652 | NC_031675.1 | FVEG_09828 | XP_018756876.1 | Cyanovirin-N domain-containing protein |
| contig00005.g3635 | NC_031675.1 | FVEG_09843 | XP_018756894.1 | Hydrophobin |
| contig00005.g3634 | NC_031675.1 | FVEG_09844 | XP_018756895.1 | Hydrophobin |
| contig00005.g3619 | NC_031675.1 | FVEG_09857 | XP_018756910.1 | Fibronectin type-III domain-containing protein |
| contig00005.g3612 | NC_031675.1 | FVEG_09861 | XP_018756916.1 | xylan 1,4-beta-xylosidase (EC 3.2.1.37) |
| contig00005.g3564 | NC_031675.1 | FVEG_09913 | XP_018756973.1 | Uncharacterized protein |
| contig00005.g3562 | NC_031675.1 | FVEG_09915 | XP_018756976.1 | Apple domain-containing protein |
| contig00005.g3543 | NC_031675.1 | FVEG_09935 | XP_018756996.1 | Ig-like domain-containing protein |
| contig00005.g3535 | NC_031675.1 | FVEG_09943 | XP_018757004.1 | Uncharacterized protein |
| contig00005.g3532 | NC_031675.1 | FVEG_09947 | XP_018757008.1 | Carboxylic ester hydrolase (EC 3.1.1.-) |
| contig00005.g3523 | NC_031675.1 | FVEG_09955 | XP_018757017.1 | SCP domain-containing protein |
| contig00001.g3 | NC_031675.1 | FVEG_14562 | XP_018741976.1 | Cytochrome P450 oxidoreductase |
| contig00001.g51 | NC_031675.1 | FVEG_14574 | XP_018742040.1 | Uncharacterized protein |
| contig00001.g167 | NC_031675.1 | FVEG_14597 | XP_018742178.1 | SGNH hydrolase-type esterase domain-containing protein |
| contig00001.g491 | NC_031675.1 | FVEG_14670 | XP_018742654.1 | Uncharacterized protein |
| contig00005.g3640 | NC_031675.1 | FVEG_16679 | XP_018756890.1 | Trichothecene biosynthesis protein |
| contig00005.g3620 | NC_031675.1 | FVEG_16681 | XP_018756909.1 | Uncharacterized protein |
| contig00005.g3526 | NC_031675.1 | FVEG_16697 | XP_018757014.1 | Zn(2)-C6 fungal-type domain-containing protein |
| contig00031.g12848 | NC_031676.1 | FVEG_03433 | XP_018747485.1 | Alkaline foam protein B |
| contig00031.g12857 | NC_031676.1 | FVEG_03442 | XP_018747495.1 | WSC domain-containing protein |
| contig00031.g12859 | NC_031676.1 | FVEG_03444 | XP_018747497.1 | PBP domain-containing protein |
| contig00031.g12874 | NC_031676.1 | FVEG_03457 | XP_018747512.1 | Beta-lactamase-related domain-containing protein |
| contig00031.g12880 | NC_031676.1 | FVEG_03463 | XP_018747518.1 | mannan endo-1,4-beta-mannosidase (EC 3.2.1.78) |
| contig00031.g12882 | NC_031676.1 | FVEG_03465 | XP_018747520.1 | Apple domain-containing protein |
| contig00031.g12908 | NC_031676.1 | FVEG_03492 | XP_018747544.1 | Killer toxin Kp4 domain-containing protein |
| contig00031.g12910 | NC_031676.1 | FVEG_03494 | XP_018747547.1 | tripeptidyl-peptidase II (EC 3.4.14.10) |
| contig00031.g12919 | NC_031676.1 | FVEG_03505 | XP_018747565.1 | Uncharacterized protein |
| contig00031.g12920 | NC_031676.1 | FVEG_03506 | XP_018747566.1 | lytic cellulose monooxygenase (C4-dehydrogenating) (EC 1.14.99.56) |
| contig00031.g12933 | NC_031676.1 | FVEG_03519 | XP_018747580.1 | Pectate lyase (EC 4.2.2.2) |
| contig00031.g12936 | NC_031676.1 | FVEG_03522 | XP_018747584.1 | Uncharacterized protein |
| contig00031.g12937 | NC_031676.1 | FVEG_03523 | XP_018747587.1 | Killer toxin Kp4 domain-containing protein |
| contig00031.g12938 | NC_031676.1 | FVEG_03524 | XP_018747588.1 | Killer toxin Kp4 domain-containing protein |
| contig00031.g12939 | NC_031676.1 | FVEG_03525 | XP_018747589.1 | Killer toxin Kp4 domain-containing protein |
| contig00031.g12940 | NC_031676.1 | FVEG_03526 | XP_018747590.1 | Glucose-methanol-choline oxidoreductase |
| contig00031.g12942 | NC_031676.1 | FVEG_03528 | XP_018747593.1 | Uncharacterized protein |
| contig00031.g12943 | NC_031676.1 | FVEG_03529 | XP_018747594.1 | Ig-like domain-containing protein |
| contig00031.g12983 | NC_031676.1 | FVEG_03576 | XP_018747651.1 | Uncharacterized protein |
| contig00031.g12984 | NC_031676.1 | FVEG_03577 | XP_018747652.1 | Endoplasmic oxidoreductin-1 |
| contig00031.g12999 | NC_031676.1 | FVEG_03591 | XP_018747671.1 | Uncharacterized protein |
| contig00031.g13022 | NC_031676.1 | FVEG_03617 | XP_018747710.1 | LysM domain-containing protein |
| contig00031.g13025 | NC_031676.1 | FVEG_03620 | XP_018747713.1 | Uncharacterized protein |
| contig00031.g13028 | NC_031676.1 | FVEG_03623 | XP_018747716.1 | LysM domain-containing protein |
| contig00007.g4793 | NC_031676.1 | FVEG_03639 | XP_018747739.1 | LicD/FKTN/FKRP nucleotidyltransferase domain-containing protein |
| contig00007.g4796 | NC_031676.1 | FVEG_03642 | XP_018747742.1 | beta-glucosidase (EC 3.2.1.21) |
| contig00007.g4802 | NC_031676.1 | FVEG_03648 | XP_018747751.1 | Dipeptidase |
| contig00007.g4806 | NC_031676.1 | FVEG_03654 | XP_018747758.1 | Uncharacterized protein |
| contig00007.g4809 | NC_031676.1 | FVEG_03657 | XP_018747762.1 | Glutathione hydrolase (EC 2.3.2.2) (EC 3.4.19.13) |
| contig00007.g4816 | NC_031676.1 | FVEG_03664 | XP_018747769.1 | Kelch repeat-containing protein |
| contig00007.g4820 | NC_031676.1 | FVEG_03669 | XP_018747775.1 | Ubiquitin 3 binding protein But2 C-terminal domain-containing protein |
| contig00007.g4826 | NC_031676.1 | FVEG_03675 | XP_018747782.1 | Carboxylic ester hydrolase (EC 3.1.1.-) |
| contig00007.g4833 | NC_031676.1 | FVEG_03682 | XP_018747791.1 | Uncharacterized protein |
| contig00007.g4834 | NC_031676.1 | FVEG_03683 | XP_018747792.1 | Uncharacterized protein |
| contig00007.g4835 | NC_031676.1 | FVEG_03684 | XP_018747793.1 | Peptidase M43 pregnancy-associated plasma |
| contig00007.g4844 | NC_031676.1 | FVEG_03690 | XP_018747803.1 | Uncharacterized protein |
| contig00007.g4847 | NC_031676.1 | FVEG_03694 | XP_018747806.1 | L-amino-acid oxidase |
| contig00007.g4861 | NC_031676.1 | FVEG_03708 | XP_018747826.1 | Killer toxin Kp4 domain-containing protein |
| contig00007.g4879 | NC_031676.1 | FVEG_03727 | XP_018747845.1 | Phosphatidylcholine-sterol acyltransferase |
| contig00007.g4890 | NC_031676.1 | FVEG_03738 | XP_018747858.1 | GEgh 16 protein |
| contig00007.g4906 | NC_031676.1 | FVEG_03751 | XP_018747876.1 | Uncharacterized protein |
| contig00005.g3516 | NC_031676.1 | FVEG_03871 | XP_018748066.1 | Protein Zds1 C-terminal domain-containing protein |
| contig00007.g5062 | NC_031676.1 | FVEG_03905 | XP_018748115.1 | Pectate lyase C |
| contig00007.g5063 | NC_031676.1 | FVEG_03906 | XP_018748116.1 | GH16 domain-containing protein |
| contig00007.g5085 | NC_031676.1 | FVEG_03930 | XP_018748143.1 | Peptidase S8/S53 domain-containing protein |
| contig00007.g5113 | NC_031676.1 | FVEG_03960 | XP_018748188.1 | Peptide hydrolase (EC 3.4.-.-) |
| contig00007.g5124 | NC_031676.1 | FVEG_03971 | XP_018748206.1 | Circumsporozoite protein |
| contig00007.g5142 | NC_031676.1 | FVEG_03990 | XP_018748229.1 | Agglutinin-like protein 2 |
| contig00007.g5149 | NC_031676.1 | FVEG_03997 | XP_018748236.1 | Rhamnogalacturonase A/B/Epimerase-like pectate lyase domain |
| contig00007.g5172 | NC_031676.1 | FVEG_04019 | XP_018748269.1 | Endoplasmic reticulum chaperone BIP (EC 3.6.4.10) |
| contig00007.g5175 | NC_031676.1 | FVEG_04022 | XP_018748272.1 | Endonuclease/exonuclease/phosphatase domain-containing protein |
| contig00007.g5222 | NC_031676.1 | FVEG_04071 | XP_018748340.1 | Tyrosinase copper-binding domain-containing protein |
| contig00007.g5249 | NC_031676.1 | FVEG_04097 | XP_018748385.1 | SCP domain-containing protein |
| contig00007.g5273 | NC_031676.1 | FVEG_04124 | XP_018748428.1 | Uncharacterized protein |
| contig00007.g5289 | NC_031676.1 | FVEG_04141 | XP_018748454.1 | Phosphatidylglycerol/phosphatidylinositol transfer protein |
| contig00035.g13536 | NC_031676.1 | FVEG_04196 | XP_018748557.1 | Laccase-2 |
| contig00035.g13603 | NC_031676.1 | FVEG_04262 | XP_018748655.1 | Ecp2 effector protein-like domain-containing protein |
| contig00035.g13607 | NC_031676.1 | FVEG_04266 | XP_018748664.1 | Glycolipid 2-alpha-mannosyltransferase |
| contig00035.g13618 | NC_031676.1 | FVEG_04278 | XP_018748677.1 | Uncharacterized protein |
| contig00012.g7656 | NC_031676.1 | FVEG_05801 | XP_018751007.1 | galacturonan 1,4-alpha-galacturonidase (EC 3.2.1.67) |
| contig00012.g7654 | NC_031676.1 | FVEG_05803 | XP_018751009.1 | WSC domain-containing protein |
| contig00012.g7644 | NC_031676.1 | FVEG_05815 | XP_018751028.1 | Uncharacterized protein |
| contig00012.g7638 | NC_031676.1 | FVEG_05821 | XP_018751037.1 | Uncharacterized protein |
| contig00012.g7620 | NC_031676.1 | FVEG_05840 | XP_018751062.1 | Uncharacterized protein |
| contig00012.g7612 | NC_031676.1 | FVEG_05849 | XP_018751073.1 | Agglutinin-like protein 2 |
| contig00012.g7595 | NC_031676.1 | FVEG_05867 | XP_018751093.1 | Ig-like domain-containing protein |
| contig00012.g7590 | NC_031676.1 | FVEG_05871 | XP_018751100.1 | GH16 domain-containing protein |
| contig00012.g7566 | NC_031676.1 | FVEG_05898 | XP_018751128.1 | Uncharacterized protein |
| contig00012.g7565 | NC_031676.1 | FVEG_05899 | XP_018751130.1 | Cupin type-1 domain-containing protein |
| contig00012.g7564 | NC_031676.1 | FVEG_05900 | XP_018751131.1 | Apple domain-containing protein |
| contig00016.g9160 | NC_031676.1 | FVEG_05913 | XP_018751145.1 | Zn(2)-C6 fungal-type domain-containing protein |
| contig00012.g7522 | NC_031676.1 | FVEG_05942 | XP_018751181.1 | LysM domain-containing protein |
| contig00012.g7511 | NC_031676.1 | FVEG_05945 | XP_018751184.1 | Hydrophobin |
| contig00012.g7514 | NC_031676.1 | FVEG_05948 | XP_018751188.1 | Uncharacterized protein |
| contig00012.g7434 | NC_031676.1 | FVEG_06027 | XP_018751290.1 | Acetylornithine deacetylase |
| contig00012.g7426 | NC_031676.1 | FVEG_06038 | XP_018751307.1 | Pheromone protein 1 |
| contig00012.g7425 | NC_031676.1 | FVEG_06039 | XP_018751308.1 | 3-phytase |
| contig00012.g7400 | NC_031676.1 | FVEG_06068 | XP_018751350.1 | Cupin type-1 domain-containing protein |
| contig00012.g7302 | NC_031676.1 | FVEG_06174 | XP_018751543.1 | lytic cellulose monooxygenase (C4-dehydrogenating) (EC 1.14.99.56) |
| contig00012.g7282 | NC_031676.1 | FVEG_06194 | XP_018751574.1 | Cell wall protein SED1 |
| contig00012.g7266 | NC_031676.1 | FVEG_06212 | XP_018751600.1 | Methyl-accepting transducer domain-containing protein |
| contig00024.g11452 | NC_031676.1 | FVEG_06297 | XP_018751729.1 | FAS1 domain-containing protein |
| contig00024.g11586 | NC_031676.1 | FVEG_06439 | XP_018751951.1 | Arabinosidase |
| contig00024.g11590 | NC_031676.1 | FVEG_06442 | XP_018751956.1 | Carboxylic ester hydrolase (EC 3.1.1.-) |
| contig00024.g11608 | NC_031676.1 | FVEG_06459 | XP_018751992.1 | lytic cellulose monooxygenase (C4-dehydrogenating) (EC 1.14.99.56) |
| contig00031.g12850 | NC_031676.1 | FVEG_15264 | XP_018747488.1 | WSC domain-containing protein |
| contig00031.g12884 | NC_031676.1 | FVEG_15271 | XP_018747521.1 | Helicase ATP-binding domain-containing protein |
| contig00031.g13027 | NC_031676.1 | FVEG_15301 | XP_018747715.1 | Secreted in xylem 14 |
| contig00007.g4778 | NC_031676.1 | FVEG_15302 | XP_018747719.1 | Oxidoreductase |
| contig00007.g4780 | NC_031676.1 | FVEG_15303 | XP_018747721.1 | Major facilitator superfamily (MFS) profile domain-containing protein |
| contig00007.g4977 | NC_031676.1 | FVEG_15341 | XP_018747995.1 | Ig-like domain-containing protein |
| contig00007.g5053 | NC_031676.1 | FVEG_15359 | XP_018748102.1 | SNF2 N-terminal domain-containing protein |
| contig00012.g7513 | NC_031676.1 | FVEG_15768 | XP_018751187.1 | Uncharacterized protein |
| contig00003.g2539 | NC_031677.1 | FVEG_05347 | XP_018750393.1 | WSC domain-containing protein |
| contig00003.g2496 | NC_031677.1 | FVEG_05394 | XP_018750471.1 | Extracellular membrane protein CFEM domain-containing protein |
| contig00003.g2464 | NC_031677.1 | FVEG_05433 | XP_018750532.1 | Questionable protein |
| contig00003.g2400 | NC_031677.1 | FVEG_05497 | XP_018750627.1 | Thioredoxin 1 |
| contig00003.g2388 | NC_031677.1 | FVEG_05503 | XP_018750640.1 | Uncharacterized protein |
| contig00003.g2384 | NC_031677.1 | FVEG_05507 | XP_018750644.1 | Rhamnogalacturonase A/B/Epimerase-like pectate lyase domain |
| contig00003.g2382 | NC_031677.1 | FVEG_05510 | XP_018750649.1 | Peptidase M20 domain-containing protein 2 |
| contig00003.g2378 | NC_031677.1 | FVEG_05513 | XP_018750653.1 | Alkaline phosphatase |
| contig00003.g2368 | NC_031677.1 | FVEG_05521 | XP_018750667.1 | beta-glucosidase (EC 3.2.1.21) |
| contig00003.g2350 | NC_031677.1 | FVEG_05540 | XP_018750690.1 | Carboxylic ester hydrolase (EC 3.1.1.-) |
| contig00003.g2331 | NC_031677.1 | FVEG_05561 | XP_018750726.1 | Peptidase S8/S53 domain-containing protein |
| contig00003.g2300 | NC_031677.1 | FVEG_05596 | XP_018750763.1 | Chitin binding protein |
| contig00003.g2292 | NC_031677.1 | FVEG_05601 | XP_018750771.1 | Peptide hydrolase (EC 3.4.-.-) |
| contig00003.g2253 | NC_031677.1 | FVEG_05642 | XP_018750821.1 | Chitin-binding type-4 domain-containing protein |
| contig00003.g2246 | NC_031677.1 | FVEG_05650 | XP_018750831.1 | Peptidase A1 domain-containing protein |
| contig00003.g2242 | NC_031677.1 | FVEG_05654 | XP_018750835.1 | AB hydrolase-1 domain-containing protein |
| contig00003.g2238 | NC_031677.1 | FVEG_05658 | XP_018750839.1 | Carboxypeptidase D |
| contig00003.g2214 | NC_031677.1 | FVEG_05679 | XP_018750867.1 | Expansin-like EG45 domain-containing protein |
| contig00003.g2203 | NC_031677.1 | FVEG_05689 | XP_018750882.1 | non-reducing end alpha-L-arabinofuranosidase (EC 3.2.1.55) |
| contig00003.g2200 | NC_031677.1 | FVEG_05691 | XP_018750885.1 | AB hydrolase-1 domain-containing protein |
| contig00003.g2196 | NC_031677.1 | FVEG_05694 | XP_018750889.1 | Uncharacterized protein |
| contig00003.g2193 | NC_031677.1 | FVEG_05697 | XP_018750892.1 | Murein transglycosylase |
| contig00003.g2190 | NC_031677.1 | FVEG_05699 | XP_018750894.1 | Amidohydrolase ytcJ-like |
| contig00003.g2189 | NC_031677.1 | FVEG_05700 | XP_018750895.1 | pectin lyase (EC 4.2.2.10) |
| contig00003.g2176 | NC_031677.1 | FVEG_05714 | XP_018750907.1 | Uncharacterized protein |
| contig00003.g2173 | NC_031677.1 | FVEG_05717 | XP_018750910.1 | PA14 domain-containing protein |
| contig00003.g2166 | NC_031677.1 | FVEG_05726 | XP_018750918.1 | Manganese lipoxygenase (EC 1.13.11.45) |
| contig00003.g2162 | NC_031677.1 | FVEG_05729 | XP_018750922.1 | Apple domain-containing protein |
| contig00003.g2161 | NC_031677.1 | FVEG_05730 | XP_018750923.1 | Apple domain-containing protein |
| contig00003.g2154 | NC_031677.1 | FVEG_05738 | XP_018750934.1 | Ecp2 effector protein domain-containing protein |
| contig00003.g2153 | NC_031677.1 | FVEG_05739 | XP_018750935.1 | Uncharacterized protein |
| contig00003.g2152 | NC_031677.1 | FVEG_05740 | XP_018750936.1 | Hydrophobin |
| contig00003.g2146 | NC_031677.1 | FVEG_05745 | XP_018750943.1 | Uncharacterized protein |
| contig00003.g2136 | NC_031677.1 | FVEG_05753 | XP_018750953.1 | Intradiol ring-cleavage dioxygenases domain-containing protein |
| contig00003.g2126 | NC_031677.1 | FVEG_05763 | XP_018750963.1 | Peptidase A1 domain-containing protein |
| contig00003.g2125 | NC_031677.1 | FVEG_05764 | XP_018750964.1 | Amidase domain-containing protein |
| contig00003.g2118 | NC_031677.1 | FVEG_05772 | XP_018750973.1 | NodB homology domain-containing protein |
| contig00003.g2116 | NC_031677.1 | FVEG_05774 | XP_018750975.1 | Uncharacterized protein |
| contig00003.g2112 | NC_031677.1 | FVEG_05778 | XP_018750980.1 | Linalool dehydratase/isomerase domain-containing protein |
| contig00015.g8562 | NC_031677.1 | FVEG_07833 | XP_018754006.1 | Uncharacterized protein |
| contig00015.g8619 | NC_031677.1 | FVEG_07889 | XP_018754089.1 | alkaline phosphatase (EC 3.1.3.1) |
| contig00015.g8709 | NC_031677.1 | FVEG_07981 | XP_018754243.1 | protein disulfide-isomerase (EC 5.3.4.1) |
| contig00027.g12245 | NC_031677.1 | FVEG_08190 | XP_018754597.1 | GmrSD restriction endonucleases C-terminal domain |
| contig00020.g10583 | NC_031677.1 | FVEG_12524 | XP_018760458.1 | Carboxylic ester hydrolase (EC 3.1.1.-) |
| contig00020.g10581 | NC_031677.1 | FVEG_12526 | XP_018760461.1 | Metallo-beta-lactamase domain-containing protein |
| contig00020.g10568 | NC_031677.1 | FVEG_12539 | XP_018760478.1 | AB hydrolase-1 domain-containing protein |
| contig00020.g10561 | NC_031677.1 | FVEG_12546 | XP_018760488.1 | Carboxypeptidase M14B (Carboxypeptidase MCPB) |
| contig00020.g10554 | NC_031677.1 | FVEG_12553 | XP_018760496.1 | alpha-L-fucosidase (EC 3.2.1.51) |
| contig00020.g10521 | NC_031677.1 | FVEG_12585 | XP_018760536.1 | Uncharacterized protein |
| contig00031.g12887 | NC_031677.1 | FVEG_12594 | XP_018760553.1 | glucan 1,3-beta-glucosidase (EC 3.2.1.58) |
| contig00020.g10500 | NC_031677.1 | FVEG_12602 | XP_018760566.1 | Uncharacterized protein |
| contig00020.g10484 | NC_031677.1 | FVEG_12615 | XP_018760585.1 | Amine oxidase domain-containing protein |
| contig00020.g10482 | NC_031677.1 | FVEG_12617 | XP_018760587.1 | NACHT domain-containing protein |
| contig00020.g10456 | NC_031677.1 | FVEG_12643 | XP_018760616.1 | chitinase (EC 3.2.1.14) |
| contig00020.g10435 | NC_031677.1 | FVEG_12666 | XP_018760641.1 | Alpha-L-arabinofuranosidase (EC 3.2.1.55) |
| contig00020.g10432 | NC_031677.1 | FVEG_12670 | XP_018760644.1 | Cyanovirin-N domain-containing protein |
| contig00020.g10421 | NC_031677.1 | FVEG_12682 | XP_018760659.1 | Uncharacterized protein |
| contig00020.g10401 | NC_031677.1 | FVEG_12704 | XP_018760685.1 | chitinase (EC 3.2.1.14) |
| contig00020.g10387 | NC_031677.1 | FVEG_12716 | XP_018760700.1 | Uncharacterized protein |
| contig00020.g10384 | NC_031677.1 | FVEG_12719 | XP_018760703.1 | Dockerin type 1 |
| contig00020.g10381 | NC_031677.1 | FVEG_12722 | XP_018760709.1 | mannan endo-1,4-beta-mannosidase (EC 3.2.1.78) |
| contig00020.g10375 | NC_031677.1 | FVEG_12728 | XP_018760716.1 | PA14 domain-containing protein |
| contig00020.g10370 | NC_031677.1 | FVEG_12733 | XP_018760724.1 | FAD/NAD(P)-binding domain-containing protein |
| contig00020.g10360 | NC_031677.1 | FVEG_12744 | XP_018760740.1 | galacturonan 1,4-alpha-galacturonidase (EC 3.2.1.67) |
| contig00020.g10339 | NC_031677.1 | FVEG_12765 | XP_018760771.1 | Extracellular serine-rich protein |
| contig00020.g10338 | NC_031677.1 | FVEG_12766 | XP_018760772.1 | pectinesterase (EC 3.1.1.11) |
| contig00020.g10333 | NC_031677.1 | FVEG_12770 | XP_018760777.1 | Apple domain-containing protein |
| contig00020.g10323 | NC_031677.1 | FVEG_12780 | XP_018760792.1 | Purine nucleoside permease |
| contig00020.g10297 | NC_031677.1 | FVEG_12804 | XP_018760835.1 | chitinase (EC 3.2.1.14) |
| contig00020.g10296 | NC_031677.1 | FVEG_12805 | XP_018760836.1 | glycerophosphodiester phosphodiesterase (EC 3.1.4.46) |
| contig00015.g8628 | NC_031677.1 | FVEG_16208 | XP_018754104.1 | Uncharacterized protein |
| contig00027.g12280 | NC_031677.1 | FVEG_16262 | XP_018754546.1 | BZIP domain-containing protein |
| contig00020.g10510 | NC_031677.1 | FVEG_17305 | XP_018760550.1 | Invertebrate defensins family profile domain-containing protein |
| contig00020.g10490 | NC_031677.1 | FVEG_17310 | XP_018760579.1 | Uncharacterized protein |
| contig00020.g10406 | NC_031677.1 | FVEG_17330 | XP_018760680.1 | Uncharacterized protein |
| contig00002.g2076 | NC_031678.1 | FVEG_04310 | XP_018748724.1 | Purple acid phosphatase (EC 3.1.3.2) |
| contig00002.g2061 | NC_031678.1 | FVEG_04325 | XP_018748747.1 | Linalool dehydratase/isomerase domain-containing protein |
| contig00002.g2060 | NC_031678.1 | FVEG_04326 | XP_018748748.1 | Uncharacterized protein |
| contig00002.g2033 | NC_031678.1 | FVEG_04351 | XP_018748778.1 | Uncharacterized protein |
| contig00002.g2016 | NC_031678.1 | FVEG_04367 | XP_018748798.1 | Uncharacterized protein |
| contig00002.g2015 | NC_031678.1 | FVEG_04368 | XP_018748799.1 | Amine oxidase (EC 1.4.3.-) |
| contig00002.g2014 | NC_031678.1 | FVEG_04369 | XP_018748800.1 | Amidase domain-containing protein |
| contig00002.g1990 | NC_031678.1 | FVEG_04390 | XP_018748826.1 | FAD-binding domain-containing protein |
| contig00002.g1978 | NC_031678.1 | FVEG_04402 | XP_018748838.1 | Carboxypeptidase (EC 3.4.16.-) |
| contig00002.g1957 | NC_031678.1 | FVEG_04421 | XP_018748860.1 | Beta-glucanase |
| contig00002.g1930 | NC_031678.1 | FVEG_04450 | XP_018748890.1 | Uncharacterized protein |
| contig00002.g1923 | NC_031678.1 | FVEG_04457 | XP_018748900.1 | Chitin binding protein |
| contig00002.g1920 | NC_031678.1 | FVEG_04460 | XP_018748904.1 | Murein transglycosylase |
| contig00002.g1879 | NC_031678.1 | FVEG_04501 | XP_018748955.1 | 50S ribosomal protein L1 |
| contig00002.g1851 | NC_031678.1 | FVEG_04529 | XP_018748992.1 | Fungal lipase-type domain-containing protein |
| contig00043.g14114 | NC_031678.1 | FVEG_04535 | XP_018748998.1 | CAAX prenyl protease (EC 3.4.24.84) |
| contig00002.g1826 | NC_031678.1 | FVEG_04555 | XP_018749028.1 | Beta-lactamase-related domain-containing protein |
| contig00002.g1822 | NC_031678.1 | FVEG_04558 | XP_018749032.1 | FMN hydroxy acid dehydrogenase domain-containing protein |
| contig00002.g1820 | NC_031678.1 | FVEG_04559 | XP_018749034.1 | Carboxylic ester hydrolase (EC 3.1.1.-) |
| contig00002.g1812 | NC_031678.1 | FVEG_04567 | XP_018749053.1 | Uncharacterized protein |
| contig00002.g1775 | NC_031678.1 | FVEG_04601 | XP_018749106.1 | Chitinase |
| contig00002.g1766 | NC_031678.1 | FVEG_04610 | XP_018749116.1 | Glucose-methanol-choline oxidoreductase |
| contig00002.g1734 | NC_031678.1 | FVEG_04647 | XP_018749173.1 | Necrosis inducing protein |
| contig00002.g1701 | NC_031678.1 | FVEG_04685 | XP_018749238.1 | Prenylcysteine oxidase/farnesylcysteine lyase |
| contig00002.g1665 | NC_031678.1 | FVEG_04722 | XP_018749305.1 | Spore coat protein SP96 |
| contig00002.g1641 | NC_031678.1 | FVEG_04743 | XP_018749339.1 | lytic cellulose monooxygenase (C4-dehydrogenating) (EC 1.14.99.56) |
| contig00002.g1633 | NC_031678.1 | FVEG_04751 | XP_018749347.1 | rhamnogalacturonan endolyase (EC 4.2.2.23) |
| contig00002.g1618 | NC_031678.1 | FVEG_04765 | XP_018749376.1 | DUF7907 domain-containing protein |
| contig00002.g1551 | NC_031678.1 | FVEG_04834 | XP_018749480.1 | Heterokaryon incompatibility Het-C |
| contig00002.g1545 | NC_031678.1 | FVEG_04840 | XP_018749495.1 | CYTH domain-containing protein |
| contig00002.g1542 | NC_031678.1 | FVEG_04842 | XP_018749497.1 | Pirin (Iron-binding nuclear protein) |
| contig00002.g1501 | NC_031678.1 | FVEG_04883 | XP_018749567.1 | Mannosyl-oligosaccharide glucosidase (EC 3.2.1.106) (Glucosidase I) |
| contig00002.g1455 | NC_031678.1 | FVEG_04931 | XP_018749671.1 | DJ-1/PfpI domain-containing protein |
| contig00002.g1438 | NC_031678.1 | FVEG_04947 | XP_018749701.1 | Glucoamylase (EC 3.2.1.3) |
| contig00002.g1416 | NC_031678.1 | FVEG_04968 | XP_018749735.1 | Carbohydrate-binding module family 19 domain-containing protein |
| contig00002.g1352 | NC_031678.1 | FVEG_05034 | XP_018749840.1 | Uncharacterized protein |
| contig00039.g13978 | NC_031678.1 | FVEG_05076 | XP_018749917.1 | CHRD domain-containing protein |
| contig00039.g13985 | NC_031678.1 | FVEG_05084 | XP_018749925.1 | Tetratricopeptide repeat and J domain-containing co-chaperone DNJ1 |
| contig00018.g9920 | NC_031678.1 | FVEG_11810 | XP_018759555.1 | Uncharacterized protein |
| contig00018.g9906 | NC_031678.1 | FVEG_11824 | XP_018759572.1 | 6-methylsalicylate decarboxylase (EC 4.1.1.52) |
| contig00018.g9903 | NC_031678.1 | FVEG_11827 | XP_018759576.1 | Apple domain-containing protein |
| contig00018.g9878 | NC_031678.1 | FVEG_11849 | XP_018759604.1 | Beta-xylanase (EC 3.2.1.8) |
| contig00018.g9874 | NC_031678.1 | FVEG_11851 | XP_018759608.1 | Aggrecan core protein |
| contig00018.g9856 | NC_031678.1 | FVEG_11867 | XP_018759628.1 | cellulase (EC 3.2.1.4) |
| contig00018.g9854 | NC_031678.1 | FVEG_11869 | XP_018759631.1 | SGNH hydrolase-type esterase domain-containing protein |
| contig00018.g9849 | NC_031678.1 | FVEG_11874 | XP_018759636.1 | Acid phosphatase |
| contig00018.g9842 | NC_031678.1 | FVEG_11882 | XP_018759643.1 | Peptidase A1 domain-containing protein |
| contig00018.g9834 | NC_031678.1 | FVEG_11889 | XP_018759653.1 | Carboxylic ester hydrolase (EC 3.1.1.-) |
| contig00018.g9832 | NC_031678.1 | FVEG_11891 | XP_018759655.1 | AB hydrolase-1 domain-containing protein |
| contig00018.g9823 | NC_031678.1 | FVEG_11901 | XP_018759665.1 | Glucose-methanol-choline oxidoreductase |
| contig00018.g9822 | NC_031678.1 | FVEG_11902 | XP_018759666.1 | DUF5703 domain-containing protein |
| contig00018.g9819 | NC_031678.1 | FVEG_11904 | XP_018759671.1 | Uncharacterized protein |
| contig00018.g9812 | NC_031678.1 | FVEG_11911 | XP_018759683.1 | FTP domain-containing protein |
| contig00018.g9806 | NC_031678.1 | FVEG_11917 | XP_018759689.1 | 3-carboxymuconate cyclase |
| contig00018.g9790 | NC_031678.1 | FVEG_11933 | XP_018759708.1 | Beta-fructofuranosidase |
| contig00018.g9788 | NC_031678.1 | FVEG_11934 | XP_018759710.1 | FAD/NAD(P)-binding domain-containing protein |
| contig00018.g9777 | NC_031678.1 | FVEG_11944 | XP_018759723.1 | Endo-1,3(4)-beta-glucanase |
| contig00018.g9772 | NC_031678.1 | FVEG_11948 | XP_018759728.1 | L-ascorbate oxidase |
| contig00018.g9767 | NC_031678.1 | FVEG_11952 | XP_018759735.1 | SGNH hydrolase-type esterase domain-containing protein |
| contig00018.g9760 | NC_031678.1 | FVEG_11959 | XP_018759745.1 | Uncharacterized protein |
| contig00018.g9731 | NC_031678.1 | FVEG_11988 | XP_018759780.1 | CBM-cenC domain-containing protein |
| contig00018.g9717 | NC_031678.1 | FVEG_12001 | XP_018759795.1 | Uncharacterized protein |
| contig00030.g12739 | NC_031678.1 | FVEG_12002 | XP_018759796.1 | chitinase (EC 3.2.1.14) |
| contig00018.g9711 | NC_031678.1 | FVEG_12008 | XP_018759803.1 | SCP domain-containing protein |
| contig00018.g9705 | NC_031678.1 | FVEG_12015 | XP_018759812.1 | AB hydrolase-1 domain-containing protein |
| contig00018.g9694 | NC_031678.1 | FVEG_12024 | XP_018759822.1 | agmatinase (EC 3.5.3.11) |
| contig00018.g9639 | NC_031678.1 | FVEG_12078 | XP_018759895.1 | Lysine-specific metallo-endopeptidase domain-containing protein |
| contig00018.g9624 | NC_031678.1 | FVEG_12093 | XP_018759914.1 | VOC domain-containing protein |
| contig00018.g9611 | NC_031678.1 | FVEG_12107 | XP_018759931.1 | Carbohydrate-binding domain-containing protein |
| contig00018.g9607 | NC_031678.1 | FVEG_12110 | XP_018759935.1 | Serum paraoxonase/lactonase 3 |
| contig00018.g9593 | NC_031678.1 | FVEG_12124 | XP_018759957.1 | Endonuclease/exonuclease/phosphatase domain-containing protein |
| contig00018.g9590 | NC_031678.1 | FVEG_12127 | XP_018759959.1 | DUF7371 domain-containing protein |
| contig00018.g9582 | NC_031678.1 | FVEG_12137 | XP_018759969.1 | UDP-glucose:glycoprotein glucosyltransferase |
| contig00018.g9577 | NC_031678.1 | FVEG_12142 | XP_018759980.1 | Murein transglycosylase |
| contig00019.g10282 | NC_031678.1 | FVEG_12176 | XP_018760027.1 | DUF7908 domain-containing protein |
| contig00019.g10278 | NC_031678.1 | FVEG_12180 | XP_018760031.1 | SGNH hydrolase-type esterase domain-containing protein |
| contig00019.g10274 | NC_031678.1 | FVEG_12185 | XP_018760036.1 | Alkaline phosphatase |
| contig00019.g10266 | NC_031678.1 | FVEG_12189 | XP_018760042.1 | alpha-L-fucosidase (EC 3.2.1.51) |
| contig00019.g10261 | NC_031678.1 | FVEG_12194 | XP_018760047.1 | Carboxypeptidase A4 |
| contig00019.g10251 | NC_031678.1 | FVEG_12202 | XP_018760057.1 | Amine oxidase domain-containing protein |
| contig00019.g10250 | NC_031678.1 | FVEG_12203 | XP_018760058.1 | MOSC domain-containing protein |
| contig00019.g10245 | NC_031678.1 | FVEG_12209 | XP_018760064.1 | pectin lyase (EC 4.2.2.10) |
| contig00019.g10242 | NC_031678.1 | FVEG_12212 | XP_018760068.1 | Heme haloperoxidase family profile domain-containing protein |
| contig00019.g10241 | NC_031678.1 | FVEG_12213 | XP_018760069.1 | Glycosyltransferase family 28 N-terminal domain-containing protein |
| contig00019.g10217 | NC_031678.1 | FVEG_12235 | XP_018760094.1 | Heme haloperoxidase family profile domain-containing protein |
| contig00019.g10216 | NC_031678.1 | FVEG_12236 | XP_018760095.1 | Lipase B |
| contig00019.g10213 | NC_031678.1 | FVEG_12238 | XP_018760098.1 | Glucose-methanol-choline oxidoreductase |
| contig00019.g10207 | NC_031678.1 | FVEG_12244 | XP_018760104.1 | Peptidase S8/S53 domain-containing protein |
| contig00019.g10206 | NC_031678.1 | FVEG_12245 | XP_018760106.1 | Ecp2 effector protein-like domain-containing protein |
| contig00019.g10171 | NC_031678.1 | FVEG_12278 | XP_018760145.1 | GPI anchored protein |
| contig00019.g10170 | NC_031678.1 | FVEG_12279 | XP_018760146.1 | pectate lyase (EC 4.2.2.2) |
| contig00019.g10168 | NC_031678.1 | FVEG_12280 | XP_018760148.1 | Trypsin |
| contig00019.g10167 | NC_031678.1 | FVEG_12281 | XP_018760149.1 | Apple domain-containing protein |
| contig00019.g10165 | NC_031678.1 | FVEG_12284 | XP_018760152.1 | Antifungal protein |
| contig00019.g10164 | NC_031678.1 | FVEG_12285 | XP_018760153.1 | FAD-binding PCMH-type domain-containing protein |
| contig00019.g10160 | NC_031678.1 | FVEG_12289 | XP_018760161.1 | Alpha-galactosidase (EC 3.2.1.22) (Melibiase) |
| contig00019.g10154 | NC_031678.1 | FVEG_12295 | XP_018760167.1 | ribonuclease T1 (EC 4.6.1.24) |
| contig00019.g10150 | NC_031678.1 | FVEG_12299 | XP_018760172.1 | Glycoside hydrolase family 5 domain-containing protein |
| contig00019.g10133 | NC_031678.1 | FVEG_12315 | XP_018760194.1 | Uncharacterized protein |
| contig00019.g10118 | NC_031678.1 | FVEG_12324 | XP_018760212.1 | LysM domain-containing protein |
| contig00019.g10112 | NC_031678.1 | FVEG_12330 | XP_018760218.1 | Protein SnodProt1 |
| contig00019.g10111 | NC_031678.1 | FVEG_12331 | XP_018760219.1 | Uncharacterized protein |
| contig00019.g10098 | NC_031678.1 | FVEG_12345 | XP_018760233.1 | Xyloglucanase |
| contig00019.g10097 | NC_031678.1 | FVEG_12346 | XP_018760234.1 | cutinase (EC 3.1.1.74) |
| contig00019.g10089 | NC_031678.1 | FVEG_12353 | XP_018760242.1 | Peptidase M14 domain-containing protein |
| contig00019.g10088 | NC_031678.1 | FVEG_12354 | XP_018760243.1 | Ecp2 effector protein domain-containing protein |
| contig00019.g10072 | NC_031678.1 | FVEG_12368 | XP_018760259.1 | rhamnogalacturonan endolyase (EC 4.2.2.23) |
| contig00019.g10066 | NC_031678.1 | FVEG_12372 | XP_018760265.1 | Glucose-methanol-choline oxidoreductase |
| contig00019.g10058 | NC_031678.1 | FVEG_12381 | XP_018760276.1 | Peptidase A1 domain-containing protein |
| contig00019.g10051 | NC_031678.1 | FVEG_12387 | XP_018760282.1 | Apple domain-containing protein |
| contig00019.g10041 | NC_031678.1 | FVEG_12396 | XP_018760293.1 | CBM-cenC domain-containing protein |
| contig00019.g10040 | NC_031678.1 | FVEG_12397 | XP_018760294.1 | Ig-like domain-containing protein |
| contig00019.g10038 | NC_031678.1 | FVEG_12399 | XP_018760296.1 | Lysine-specific metallo-endopeptidase domain-containing protein |
| contig00019.g10037 | NC_031678.1 | FVEG_12400 | XP_018760297.1 | Uncharacterized protein |
| contig00019.g10034 | NC_031678.1 | FVEG_12404 | XP_018760301.1 | Tyrosinase copper-binding domain-containing protein |
| contig00019.g10015 | NC_031678.1 | FVEG_12422 | XP_018760325.1 | Uncharacterized protein |
| contig00019.g10014 | NC_031678.1 | FVEG_12423 | XP_018760326.1 | mannan endo-1,4-beta-mannosidase (EC 3.2.1.78) |
| contig00019.g10005 | NC_031678.1 | FVEG_12434 | XP_018760344.1 | Pectate lyase (EC 4.2.2.2) |
| contig00019.g10004 | NC_031678.1 | FVEG_12435 | XP_018760345.1 | Uncharacterized protein |
| contig00019.g9999 | NC_031678.1 | FVEG_12442 | XP_018760355.1 | WSC domain-containing protein |
| contig00019.g9995 | NC_031678.1 | FVEG_12445 | XP_018760359.1 | carbonic anhydrase (EC 4.2.1.1) |
| contig00019.g9992 | NC_031678.1 | FVEG_12448 | XP_018760362.1 | Uncharacterized protein |
| contig00019.g9982 | NC_031678.1 | FVEG_12459 | XP_018760377.1 | Uncharacterized protein |
| contig00019.g9958 | NC_031678.1 | FVEG_12482 | XP_018760408.1 | Pyrroloquinoline quinone-dependent pyranose dehydrogenase |
| contig00019.g9957 | NC_031678.1 | FVEG_12483 | XP_018760409.1 | Apple domain-containing protein |
| contig00019.g9956 | NC_031678.1 | FVEG_12484 | XP_018760410.1 | Apple domain-containing protein |
| contig00019.g9955 | NC_031678.1 | FVEG_12485 | XP_018760411.1 | Feruloyl esterase C (EC 3.1.1.73) (Ferulic acid esterase C) |
| contig00019.g9954 | NC_031678.1 | FVEG_12486 | XP_018760412.1 | Murein transglycosylase |
| contig00019.g9935 | NC_031678.1 | FVEG_12502 | XP_018760431.1 | Beta-xylanase (EC 3.2.1.8) |
| contig00002.g2018 | NC_031678.1 | FVEG_15461 | XP_018748796.1 | TIL domain-containing protein |
| contig00002.g1931 | NC_031678.1 | FVEG_15481 | XP_018748891.1 | Extracellular membrane protein CFEM domain-containing protein |
| contig00002.g1379 | NC_031678.1 | FVEG_15586 | XP_018749796.1 | Peptidase S8/S53 domain-containing protein |
| contig00018.g9871 | NC_031678.1 | FVEG_17120 | XP_018759611.1 | Uncharacterized protein |
| contig00019.g10268 | NC_031678.1 | FVEG_17178 | XP_018760041.1 | Glycosyl hydrolase family 32 N-terminal domain-containing protein |
| contig00019.g10169 | NC_031678.1 | FVEG_17206 | XP_018760147.1 | Uncharacterized protein |
| contig00019.g10121 | NC_031678.1 | FVEG_17224 | XP_018760208.1 | Uncharacterized protein |
| contig00019.g9981 | NC_031678.1 | FVEG_17275 | XP_018760378.1 | Uncharacterized protein |
| contig00010.g6542 | NC_031679.1 | FVEG_02601 | XP_018746211.1 | Uncharacterized protein |
| contig00010.g6566 | NC_031679.1 | FVEG_02627 | XP_018746261.1 | DNA replication factor Cdt1 C-terminal domain-containing protein |
| contig00010.g6627 | NC_031679.1 | FVEG_02691 | XP_018746400.1 | Amidohydrolase ytcJ-like |
| contig00010.g6631 | NC_031679.1 | FVEG_02695 | XP_018746409.1 | MICOS complex subunit MIC12 (Altered inheritance of mitochondria p 5) |
| contig00001.g384 | NC_031679.1 | FVEG_02824 | XP_018746630.1 | Uncharacterized protein |
| contig00004.g2910 | NC_031679.1 | FVEG_02982 | XP_018746867.1 | RlpA-like protein double-psi beta-barrel domain-containing protein |
| contig00004.g2931 | NC_031679.1 | FVEG_03005 | XP_018746906.1 | Protein SnodProt1 |
| contig00004.g2971 | NC_031679.1 | FVEG_03046 | XP_018746959.1 | Malate dehydrogenase |
| contig00004.g2999 | NC_031679.1 | FVEG_03072 | XP_018746992.1 | tripeptidyl-peptidase II (EC 3.4.14.10) |
| contig00004.g3017 | NC_031679.1 | FVEG_03090 | XP_018747023.1 | Uncharacterized protein |
| contig00004.g3048 | NC_031679.1 | FVEG_03121 | XP_018747081.1 | DnaJ-related protein SCJ1 |
| contig00004.g3052 | NC_031679.1 | FVEG_03124 | XP_018747085.1 | Laccase abr2 |
| contig00004.g3068 | NC_031679.1 | FVEG_03140 | XP_018747107.1 | Beta-xylanase (EC 3.2.1.8) |
| contig00004.g3071 | NC_031679.1 | FVEG_03143 | XP_018747112.1 | Uncharacterized protein |
| contig00004.g3072 | NC_031679.1 | FVEG_03144 | XP_018747113.1 | inorganic diphosphatase (EC 3.6.1.1) |
| contig00004.g3143 | NC_031679.1 | FVEG_03210 | XP_018747204.1 | Ig-like domain-containing protein |
| contig00004.g3175 | NC_031679.1 | FVEG_03245 | XP_018747256.1 | Subtilisin |
| contig00004.g3189 | NC_031679.1 | FVEG_03260 | XP_018747276.1 | CBM1 domain-containing protein |
| contig00004.g3204 | NC_031679.1 | FVEG_03274 | XP_018747294.1 | Uncharacterized protein |
| contig00004.g3205 | NC_031679.1 | FVEG_03275 | XP_018747295.1 | Serine/threonine protein kinase |
| contig00004.g3207 | NC_031679.1 | FVEG_03277 | XP_018747297.1 | asparaginase (EC 3.5.1.1) |
| contig00004.g3214 | NC_031679.1 | FVEG_03284 | XP_018747309.1 | Uncharacterized protein |
| contig00004.g3219 | NC_031679.1 | FVEG_03290 | XP_018747315.1 | Uncharacterized protein |
| contig00004.g3223 | NC_031679.1 | FVEG_03292 | XP_018747319.1 | Uncharacterized protein |
| contig00004.g3239 | NC_031679.1 | FVEG_03305 | XP_018747335.1 | Ecp2 effector protein domain-containing protein |
| contig00004.g3242 | NC_031679.1 | FVEG_03307 | XP_018747338.1 | Secreted in xylem 2 |
| contig00004.g3265 | NC_031679.1 | FVEG_03324 | XP_018747359.1 | Alpha-glucuronidase (EC 3.2.1.139) |
| contig00004.g3276 | NC_031679.1 | FVEG_03334 | XP_018747369.1 | Uncharacterized protein |
| contig00004.g3290 | NC_031679.1 | FVEG_03346 | XP_018747383.1 | Uncharacterized protein |
| contig00004.g3297 | NC_031679.1 | FVEG_03351 | XP_018747391.1 | Cutinase (EC 3.1.1.74) |
| contig00004.g3308 | NC_031679.1 | FVEG_03362 | XP_018747403.1 | Murein transglycosylase |
| contig00004.g3188 | NC_031679.1 | FVEG_03382 | XP_018747428.1 | Zn(2)-C6 fungal-type domain-containing protein |
| contig00004.g3339 | NC_031679.1 | FVEG_03395 | XP_018747444.1 | Cutinase (EC 3.1.1.74) |
| contig00004.g3342 | NC_031679.1 | FVEG_03398 | XP_018747447.1 | Lipase A |
| contig00004.g3360 | NC_031679.1 | FVEG_03416 | XP_018747466.1 | Alginate lyase domain-containing protein |
| contig00008.g5736 | NC_031679.1 | FVEG_08899 | XP_018755529.1 | Meiotic sister chromatid recombination protein 1 |
| contig00008.g5656 | NC_031679.1 | FVEG_08978 | XP_018755643.1 | 6-phosphogluconolactonase |
| contig00008.g5625 | NC_031679.1 | FVEG_09009 | XP_018755693.1 | AB hydrolase-1 domain-containing protein |
| contig00008.g5624 | NC_031679.1 | FVEG_09010 | XP_018755694.1 | Cupin type-1 domain-containing protein |
| contig00008.g5598 | NC_031679.1 | FVEG_09034 | XP_018755724.1 | Beta-xylosidase C-terminal Concanavalin A-like domain |
| contig00008.g5597 | NC_031679.1 | FVEG_09035 | XP_018755725.1 | ribonuclease T2 (EC 4.6.1.19) |
| contig00008.g5596 | NC_031679.1 | FVEG_09036 | XP_018755726.1 | Small secreted protein |
| contig00008.g5556 | NC_031679.1 | FVEG_09073 | XP_018755774.1 | tripeptidyl-peptidase II (EC 3.4.14.10) |
| contig00008.g5519 | NC_031679.1 | FVEG_09116 | XP_018755828.1 | lytic cellulose monooxygenase (C4-dehydrogenating) (EC 1.14.99.56) |
| contig00008.g5496 | NC_031679.1 | FVEG_09142 | XP_018755868.1 | Secreted protein NIS1 |
| contig00008.g5488 | NC_031679.1 | FVEG_09149 | XP_018755879.1 | Peroxidase (EC 1.11.1.-) |
| contig00008.g5408 | NC_031679.1 | FVEG_09233 | XP_018756050.1 | AB hydrolase-1 domain-containing protein |
| contig00008.g5359 | NC_031679.1 | FVEG_09284 | XP_018756125.1 | Endo-1,3(4)-beta-glucanase |
| contig00008.g5358 | NC_031679.1 | FVEG_09285 | XP_018756126.1 | Cellulase (EC 3.2.1.4) |
| contig00008.g5356 | NC_031679.1 | FVEG_09287 | XP_018756129.1 | Cytochrome c oxidase assembly protein |
| contig00008.g5322 | NC_031679.1 | FVEG_09321 | XP_018756173.1 | Apple domain-containing protein |
| contig00004.g3383 | NC_031679.1 | FVEG_09332 | XP_018756186.1 | Spore coat protein SP96 |
| contig00004.g3397 | NC_031679.1 | FVEG_09348 | XP_018756201.1 | Apple domain-containing protein |
| contig00004.g3399 | NC_031679.1 | FVEG_09350 | XP_018756203.1 | Apple domain-containing protein |
| contig00004.g3410 | NC_031679.1 | FVEG_09361 | XP_018756214.1 | feruloyl esterase (EC 3.1.1.73) |
| contig00004.g3419 | NC_031679.1 | FVEG_09370 | XP_018756226.1 | GH64 domain-containing protein |
| contig00004.g3427 | NC_031679.1 | FVEG_09375 | XP_018756234.1 | Peptidase S1 domain-containing protein |
| contig00003.g2666 | NC_031679.1 | FVEG_09387 | XP_018756247.1 | Pectate lyase (EC 4.2.2.2) |
| contig00004.g3455 | NC_031679.1 | FVEG_09405 | XP_018756265.1 | Acetylesterase |
| contig00004.g3456 | NC_031679.1 | FVEG_09406 | XP_018756266.1 | Uncharacterized protein |
| contig00004.g3460 | NC_031679.1 | FVEG_09409 | XP_018756269.1 | Uncharacterized protein |
| contig00004.g3469 | NC_031679.1 | FVEG_09416 | XP_018756280.1 | Uncharacterized protein |
| contig00004.g3492 | NC_031679.1 | FVEG_09438 | XP_018756308.1 | Uncharacterized protein |
| contig00004.g3495 | NC_031679.1 | FVEG_09440 | XP_018756311.1 | Trichothecene biosynthesis protein |
| contig00004.g3498 | NC_031679.1 | FVEG_09443 | XP_018756314.1 | Glucan endo-1,3-beta-glucosidase |
| contig00004.g3499 | NC_031679.1 | FVEG_09444 | XP_018756315.1 | Ecp2 effector protein domain-containing protein |
| contig00004.g2864 | NC_031679.1 | FVEG_15153 | XP_018746789.1 | Ricin B lectin domain-containing protein |
| contig00004.g3213 | NC_031679.1 | FVEG_15231 | XP_018747308.1 | Protein disulfide-isomerase (EC 5.3.4.1) |
| contig00004.g3227 | NC_031679.1 | FVEG_15235 | XP_018747323.1 | Uncharacterized protein |
| contig00008.g5577 | NC_031679.1 | FVEG_16512 | XP_018755749.1 | DUF7908 domain-containing protein |
| contig00008.g5375 | NC_031679.1 | FVEG_16549 | XP_018756097.1 | Modin |
| contig00004.g3463 | NC_031679.1 | FVEG_16576 | XP_018756272.1 | Chitin-binding type-2 domain-containing protein |
| contig00004.g3468 | NC_031679.1 | FVEG_16579 | XP_018756279.1 | Uncharacterized protein |
| contig00004.g3482 | NC_031679.1 | FVEG_16584 | XP_018756296.1 | Uncharacterized protein |
| contig00023.g11147 | NC_031680.1 | FVEG_01522 | XP_018744453.1 | Enoyl reductase (ER) domain-containing protein |
| contig00023.g11169 | NC_031680.1 | FVEG_01544 | XP_018744476.1 | Uncharacterized protein |
| contig00023.g11172 | NC_031680.1 | FVEG_01546 | XP_018744479.1 | ABC transporter |
| contig00023.g11173 | NC_031680.1 | FVEG_01547 | XP_018744480.1 | Cell surface protein |
| contig00023.g11178 | NC_031680.1 | FVEG_01553 | XP_018744487.1 | Cupin 2 conserved barrel domain-containing protein |
| contig00023.g11179 | NC_031680.1 | FVEG_01554 | XP_018744488.1 | Uncharacterized protein |
| contig00023.g11182 | NC_031680.1 | FVEG_01557 | XP_018744491.1 | Peptide hydrolase (EC 3.4.-.-) |
| contig00023.g11194 | NC_031680.1 | FVEG_01567 | XP_018744501.1 | Regulatory P domain-containing protein |
| contig00023.g11203 | NC_031680.1 | FVEG_01575 | XP_018744514.1 | Hydrophobin |
| contig00023.g11212 | NC_031680.1 | FVEG_01583 | XP_018744523.1 | chitinase (EC 3.2.1.14) |
| contig00023.g11213 | NC_031680.1 | FVEG_01584 | XP_018744524.1 | Non-secreted LysM effector LCP1 |
| contig00023.g11225 | NC_031680.1 | FVEG_01594 | XP_018744537.1 | Apple domain-containing protein |
| contig00023.g11228 | NC_031680.1 | FVEG_01596 | XP_018744540.1 | Thiaminase-2/PQQC domain-containing protein |
| contig00023.g11231 | NC_031680.1 | FVEG_01598 | XP_018744545.1 | Uncharacterized protein |
| contig00023.g11247 | NC_031680.1 | FVEG_01614 | XP_018744582.1 | Tyrosinase |
| contig00023.g11248 | NC_031680.1 | FVEG_01615 | XP_018744583.1 | WSC domain-containing protein |
| contig00023.g11257 | NC_031680.1 | FVEG_01622 | XP_018744595.1 | Extracellular membrane protein CFEM domain-containing protein |
| contig00023.g11264 | NC_031680.1 | FVEG_01630 | XP_018744610.1 | Cell wall protein PhiA |
| contig00023.g11280 | NC_031680.1 | FVEG_01647 | XP_018744629.1 | Glucosidase 2 subunit beta |
| contig00023.g11292 | NC_031680.1 | FVEG_01659 | XP_018744642.1 | Uncharacterized protein |
| contig00023.g11293 | NC_031680.1 | FVEG_01660 | XP_018744643.1 | PA14 domain-containing protein |
| contig00023.g11302 | NC_031680.1 | FVEG_01670 | XP_018744653.1 | FAD-binding domain-containing protein |
| contig00023.g11358 | NC_031680.1 | FVEG_01726 | XP_018744727.1 | Extracellular membrane protein CFEM domain-containing protein |
| contig00023.g11361 | NC_031680.1 | FVEG_01729 | XP_018744730.1 | EthD domain-containing protein |
| contig00023.g11363 | NC_031680.1 | FVEG_01731 | XP_018744732.1 | Meiotically up-regulated gene 157 protein |
| contig00023.g11374 | NC_031680.1 | FVEG_01746 | XP_018744746.1 | Apple domain-containing protein |
| contig00023.g11385 | NC_031680.1 | FVEG_01758 | XP_018744757.1 | Small secreted protein |
| contig00023.g11396 | NC_031680.1 | FVEG_01770 | XP_018744771.1 | Glucanase (EC 3.2.1.-) |
| contig00023.g11406 | NC_031680.1 | FVEG_01778 | XP_018744783.1 | Endo-chitosanase (EC 3.2.1.132) |
| contig00021.g10820 | NC_031680.1 | FVEG_01779 | XP_018744784.1 | glucan endo-1,6-beta-glucosidase (EC 3.2.1.75) |
| contig00021.g10801 | NC_031680.1 | FVEG_01795 | XP_018744804.1 | Beta-lactamase-related domain-containing protein |
| contig00021.g10794 | NC_031680.1 | FVEG_01804 | XP_018744819.1 | Neutral protease 2 (EC 3.4.24.39) (Deuterolysin) |
| contig00030.g12755 | NC_031680.1 | FVEG_01809 | XP_018744825.1 | Leucine-rich repeat-containing protein 40 |
| contig00021.g10778 | NC_031680.1 | FVEG_01819 | XP_018744836.1 | Carboxylic ester hydrolase (EC 3.1.1.-) |
| contig00021.g10727 | NC_031680.1 | FVEG_01870 | XP_018744907.1 | glucan endo-1,3-beta-D-glucosidase (EC 3.2.1.39) |
| contig00021.g10718 | NC_031680.1 | FVEG_01879 | XP_018744920.1 | Uncharacterized protein |
| contig00006.g4712 | NC_031680.1 | FVEG_02095 | XP_018745298.1 | FAD dependent oxidoreductase domain-containing protein |
| contig00006.g4676 | NC_031680.1 | FVEG_02133 | XP_018745425.1 | Alkaline proteinase |
| contig00006.g4643 | NC_031680.1 | FVEG_02166 | XP_018745476.1 | Ribosomal protein s17 |
| contig00006.g4642 | NC_031680.1 | FVEG_02167 | XP_018745479.1 | Nuclease S1 |
| contig00006.g4514 | NC_031680.1 | FVEG_02309 | XP_018745700.1 | DUF7143 domain-containing protein |
| contig00025.g11730 | NC_031680.1 | FVEG_02317 | XP_018745719.1 | Cytochrome P450 oxidoreductase |
| contig00006.g4416 | NC_031680.1 | FVEG_02403 | XP_018745857.1 | FAD-binding PCMH-type domain-containing protein |
| contig00006.g4415 | NC_031680.1 | FVEG_02404 | XP_018745858.1 | Dienelactone hydrolase domain-containing protein |
| contig00006.g4413 | NC_031680.1 | FVEG_02406 | XP_018745861.1 | AB hydrolase-1 domain-containing protein |
| contig00006.g4411 | NC_031680.1 | FVEG_02407 | XP_018745863.1 | Uncharacterized protein |
| contig00006.g4313 | NC_031680.1 | FVEG_02506 | XP_018746004.1 | Glycoside hydrolase 131 |
| contig00026.g12079 | NC_031680.1 | FVEG_13160 | XP_018761302.1 | FAS1 domain-containing protein |
| contig00026.g12064 | NC_031680.1 | FVEG_13176 | XP_018761321.1 | Uncharacterized protein |
| contig00026.g12059 | NC_031680.1 | FVEG_13181 | XP_018761327.1 | Heterokaryon incompatibility domain-containing protein |
| contig00026.g12057 | NC_031680.1 | FVEG_13183 | XP_018761329.1 | Cell wall glycosyl hydrolase YteR |
| contig00026.g12035 | NC_031680.1 | FVEG_13207 | XP_018761356.1 | Endo-1,4-beta-xylanase (EC 3.2.1.8) |
| contig00026.g11994 | NC_031680.1 | FVEG_13248 | XP_018761403.1 | AttH domain-containing protein |
| contig00026.g11988 | NC_031680.1 | FVEG_13254 | XP_018761409.1 | Apple domain-containing protein |
| contig00026.g11986 | NC_031680.1 | FVEG_13256 | XP_018761411.1 | Xaa-Pro dipeptidyl-peptidase C-terminal domain-containing protein |
| contig00026.g11981 | NC_031680.1 | FVEG_13262 | XP_018761417.1 | Sialidase domain-containing protein |
| contig00026.g11951 | NC_031680.1 | FVEG_13289 | XP_018761450.1 | Amine oxidase domain-containing protein |
| contig00026.g11941 | NC_031680.1 | FVEG_13300 | XP_018761461.1 | BNR/Asp-box repeat domain protein |
| contig00026.g11937 | NC_031680.1 | FVEG_13304 | XP_018761465.1 | Murein transglycosylase |
| contig00026.g11936 | NC_031680.1 | FVEG_13305 | XP_018761466.1 | Beta-xylosidase |
| contig00026.g11935 | NC_031680.1 | FVEG_13306 | XP_018761467.1 | Peptidase A1 domain-containing protein |
| contig00026.g11934 | NC_031680.1 | FVEG_13307 | XP_018761468.1 | Glucose-methanol-choline oxidoreductase |
| contig00026.g11930 | NC_031680.1 | FVEG_13310 | XP_018761472.1 | CBM1 domain-containing protein |
| contig00023.g11183 | NC_031680.1 | FVEG_14861 | XP_018744492.1 | NACHT domain-containing protein |
| contig00023.g11392 | NC_031680.1 | FVEG_14919 | XP_018744766.1 | Cytochrome P450 |
| contig00006.g4633 | NC_031680.1 | FVEG_14997 | XP_018745498.1 | MUC1-Extracellular alpha-1,4-glucan glucosidase |
| contig00026.g11953 | NC_031680.1 | FVEG_17483 | XP_018761448.1 | 3-carboxymuconate cyclase |
| contig00026.g11914 | NC_031680.1 | FVEG_17495 | XP_018761493.1 | SCP domain-containing protein |
| contig00011.g6783 | NC_031681.1 | FVEG_06522 | XP_018752070.1 | Galactose oxidase |
| contig00011.g6787 | NC_031681.1 | FVEG_06525 | XP_018752075.1 | Intradiol ring-cleavage dioxygenases domain-containing protein |
| contig00011.g6800 | NC_031681.1 | FVEG_06538 | XP_018752089.1 | Hydrophobin 3 |
| contig00011.g6842 | NC_031681.1 | FVEG_06580 | XP_018752139.1 | Amidase domain-containing protein |
| contig00011.g6846 | NC_031681.1 | FVEG_06584 | XP_018752144.1 | FAD-binding PCMH-type domain-containing protein |
| contig00011.g6891 | NC_031681.1 | FVEG_06631 | XP_018752222.1 | Filamentous hemagglutinin |
| contig00011.g6966 | NC_031681.1 | FVEG_06704 | XP_018752314.1 | Peptide hydrolase (EC 3.4.-.-) |
| contig00011.g6977 | NC_031681.1 | FVEG_06715 | XP_018752328.1 | Uncharacterized protein |
| contig00011.g6989 | NC_031681.1 | FVEG_06728 | XP_018752349.1 | Beta-glucosidase cel3A (EC 3.2.1.21) |
| contig00011.g7002 | NC_031681.1 | FVEG_06739 | XP_018752365.1 | Stress response protein rds1p |
| contig00011.g7012 | NC_031681.1 | FVEG_06750 | XP_018752382.1 | Palmitoyl-protein thioesterase 1 (EC 3.1.2.22) |
| contig00011.g7037 | NC_031681.1 | FVEG_06777 | XP_018752412.1 | LysM domain-containing protein |
| contig00011.g7039 | NC_031681.1 | FVEG_06779 | XP_018752414.1 | chitinase (EC 3.2.1.14) |
| contig00011.g7065 | NC_031681.1 | FVEG_06805 | XP_018752447.1 | Uncharacterized protein |
| contig00011.g7067 | NC_031681.1 | FVEG_06807 | XP_018752449.1 | SGNH hydrolase-type esterase domain-containing protein |
| contig00011.g7085 | NC_031681.1 | FVEG_06822 | XP_018752465.1 | NodB homology domain-containing protein |
| contig00011.g7096 | NC_031681.1 | FVEG_06833 | XP_018752484.1 | Glucose-methanol-choline oxidoreductase |
| contig00011.g7120 | NC_031681.1 | FVEG_06859 | XP_018752516.1 | tyrosinase (EC 1.14.18.1) |
| contig00011.g7123 | NC_031681.1 | FVEG_06863 | XP_018752526.1 | Beta-1,4-mannosyl-glycoprotein 4-beta-N-acetylglucosaminyltransferase |
| contig00011.g7125 | NC_031681.1 | FVEG_06865 | XP_018752528.1 | Trehalase (EC 3.2.1.28) (Alpha-trehalose glucohydrolase) |
| contig00011.g7160 | NC_031681.1 | FVEG_06900 | XP_018752570.1 | glucan endo-1,6-beta-glucosidase (EC 3.2.1.75) |
| contig00010.g6439 | NC_031681.1 | FVEG_07082 | XP_018752900.1 | Agmatinase |
| contig00005.g3721 | NC_031681.1 | FVEG_07105 | XP_018752948.1 | Translation initiation factor 4G |
| contig00010.g6480 | NC_031681.1 | FVEG_07124 | XP_018752991.1 | DUF7137 domain-containing protein |
| contig00040.g14028 | NC_031681.1 | FVEG_11396 | XP_018758944.1 | Inhibitor I9 domain-containing protein |
| contig00040.g14015 | NC_031681.1 | FVEG_11410 | XP_018758975.1 | Uncharacterized protein |
| contig00017.g9523 | NC_031681.1 | FVEG_11453 | XP_018759035.1 | Vacuolar protease A |
| contig00017.g9521 | NC_031681.1 | FVEG_11455 | XP_018759037.1 | Uncharacterized protein |
| contig00017.g9486 | NC_031681.1 | FVEG_11492 | XP_018759102.1 | Peptidase A1 domain-containing protein |
| contig00017.g9380 | NC_031681.1 | FVEG_11600 | XP_018759297.1 | Expansin-like EG45 domain-containing protein |
| contig00017.g9370 | NC_031681.1 | FVEG_11609 | XP_018759313.1 | Uncharacterized protein |
| contig00017.g9326 | NC_031681.1 | FVEG_11665 | XP_018759370.1 | Uncharacterized protein |
| contig00017.g9325 | NC_031681.1 | FVEG_11666 | XP_018759371.1 | Apple domain-containing protein |
| contig00017.g9324 | NC_031681.1 | FVEG_11667 | XP_018759372.1 | Amine oxidase |
| contig00015.g8551 | NC_031681.1 | FVEG_11681 | XP_018759391.1 | Anucleate primary sterigmata protein B |
| contig00017.g9300 | NC_031681.1 | FVEG_11689 | XP_018759403.1 | PA14 domain-containing protein |
| contig00017.g9291 | NC_031681.1 | FVEG_11695 | XP_018759412.1 | Probable dipeptidyl-aminopeptidase B (EC 3.4.14.5) |
| contig00017.g9286 | NC_031681.1 | FVEG_11700 | XP_018759418.1 | Carboxylic ester hydrolase (EC 3.1.1.-) |
| contig00017.g9272 | NC_031681.1 | FVEG_11714 | XP_018759432.1 | N1-acetylpolyamine oxidase |
| contig00017.g9269 | NC_031681.1 | FVEG_11717 | XP_018759435.1 | Amidohydrolase-related domain-containing protein |
| contig00017.g9266 | NC_031681.1 | FVEG_11720 | XP_018759438.1 | Uncharacterized protein |
| contig00017.g9264 | NC_031681.1 | FVEG_11722 | XP_018759441.1 | Celp0028 effector like protein |
| contig00017.g9263 | NC_031681.1 | FVEG_11723 | XP_018759442.1 | Uncharacterized protein |
| contig00017.g9253 | NC_031681.1 | FVEG_11731 | XP_018759453.1 | FAD-binding PCMH-type domain-containing protein |
| contig00017.g9247 | NC_031681.1 | FVEG_11737 | XP_018759463.1 | Uncharacterized protein |
| contig00017.g9246 | NC_031681.1 | FVEG_11738 | XP_018759464.1 | Uncharacterized protein |
| contig00017.g9234 | NC_031681.1 | FVEG_11749 | XP_018759477.1 | beta-glucosidase (EC 3.2.1.21) |
| contig00033.g13299 | NC_031681.1 | FVEG_11762 | XP_018759490.1 | Carrier domain-containing protein |
| contig00017.g9219 | NC_031681.1 | FVEG_11764 | XP_018759492.1 | Amine oxidase (EC 1.4.3.-) |
| contig00017.g9214 | NC_031681.1 | FVEG_11768 | XP_018759497.1 | AB hydrolase-1 domain-containing protein |
| contig00017.g9211 | NC_031681.1 | FVEG_11772 | XP_018759501.1 | Choline dehydrogenase |
| contig00017.g9210 | NC_031681.1 | FVEG_11773 | XP_018759502.1 | Uncharacterized protein |
| contig00017.g9195 | NC_031681.1 | FVEG_11787 | XP_018759523.1 | endo-polygalacturonase (EC 3.2.1.15) (Pectinase) |
| contig00017.g9186 | NC_031681.1 | FVEG_11796 | XP_018759532.1 | Murein transglycosylase |
| contig00011.g6802 | NC_031681.1 | FVEG_15883 | XP_018752091.1 | Glycosyltransferase 2-like domain-containing protein |
| contig00011.g6809 | NC_031681.1 | FVEG_15886 | XP_018752099.1 | C2H2-type domain-containing protein |
| contig00011.g6888 | NC_031681.1 | FVEG_15904 | XP_018752217.1 | Carboxylic ester hydrolase (EC 3.1.1.-) |
| contig00011.g6913 | NC_031681.1 | FVEG_15912 | XP_018752246.1 | Long chronological lifespan protein 2 |
| contig00011.g7140 | NC_031681.1 | FVEG_15951 | XP_018752543.1 | Uncharacterized protein |
| contig00010.g6487 | NC_031681.1 | FVEG_16023 | XP_018753000.1 | GH16 domain-containing protein |
| contig00017.g9262 | NC_031681.1 | FVEG_17080 | XP_018759443.1 | Uncharacterized protein |
| contig00017.g9171 | NC_031681.1 | FVEG_17099 | XP_018759551.1 | Carboxylesterase type B domain-containing protein |
| contig00029.g12692 | NC_031682.1 | FVEG_07167 | XP_018753073.1 | FAD-binding PCMH-type domain-containing protein |
| contig00029.g12691 | NC_031682.1 | FVEG_07168 | XP_018753074.1 | Extracellular serine-rich protein |
| contig00029.g12689 | NC_031682.1 | FVEG_07171 | XP_018753077.1 | IDI-2 |
| contig00029.g12688 | NC_031682.1 | FVEG_07172 | XP_018753078.1 | Uncharacterized protein |
| contig00029.g12687 | NC_031682.1 | FVEG_07173 | XP_018753079.1 | Endoglucanase E |
| contig00029.g12686 | NC_031682.1 | FVEG_07174 | XP_018753080.1 | Alginate lyase 2 domain-containing protein |
| contig00029.g12663 | NC_031682.1 | FVEG_07198 | XP_018753118.1 | Peptidase M14 domain-containing protein |
| contig00029.g12658 | NC_031682.1 | FVEG_07203 | XP_018753123.1 | Uncharacterized protein |
| contig00029.g12630 | NC_031682.1 | FVEG_07227 | XP_018753155.1 | N4-(Beta-N-acetylglucosaminyl)-L-asparaginase |
| contig00029.g12625 | NC_031682.1 | FVEG_07232 | XP_018753161.1 | lytic cellulose monooxygenase (C4-dehydrogenating) (EC 1.14.99.56) |
| contig00029.g12624 | NC_031682.1 | FVEG_07233 | XP_018753162.1 | Extracellular serine carboxypeptidase |
| contig00029.g12594 | NC_031682.1 | FVEG_07261 | XP_018753195.1 | Endo-1,4-beta-xylanase (EC 3.2.1.8) |
| contig00014.g8085 | NC_031682.1 | FVEG_07357 | XP_018753350.1 | Inactive metallocarboxypeptidase ECM14 |
| contig00014.g8108 | NC_031682.1 | FVEG_07382 | XP_018753393.1 | Malate dehydrogenase |
| contig00014.g8155 | NC_031682.1 | FVEG_07432 | XP_018753478.1 | Intradiol ring-cleavage dioxygenases domain-containing protein |
| contig00014.g8164 | NC_031682.1 | FVEG_07441 | XP_018753490.1 | Tyrosinase |
| contig00014.g8170 | NC_031682.1 | FVEG_07448 | XP_018753497.1 | Amidohydrolase ytcJ-like |
| contig00014.g8199 | NC_031682.1 | FVEG_07478 | XP_018753540.1 | CPAF-like PDZ domain-containing protein |
| contig00014.g8210 | NC_031682.1 | FVEG_07490 | XP_018753552.1 | Alpha-L-arabinofuranosidase II |
| contig00014.g8216 | NC_031682.1 | FVEG_07496 | XP_018753559.1 | Uncharacterized protein |
| contig00014.g8231 | NC_031682.1 | FVEG_07510 | XP_018753579.1 | Superoxide dismutase (EC 1.15.1.1) |
| contig00014.g8232 | NC_031682.1 | FVEG_07511 | XP_018753580.1 | Uncharacterized protein |
| contig00014.g8240 | NC_031682.1 | FVEG_07518 | XP_018753588.1 | Alpha-galactosidase (EC 3.2.1.22) (Melibiase) |
| contig00014.g8293 | NC_031682.1 | FVEG_07571 | XP_018753681.1 | AB hydrolase-1 domain-containing protein |
| contig00014.g8294 | NC_031682.1 | FVEG_07572 | XP_018753682.1 | Kazal-like domain-containing protein |
| contig00014.g8312 | NC_031682.1 | FVEG_07588 | XP_018753697.1 | LysM domain-containing protein |
| contig00014.g8328 | NC_031682.1 | FVEG_07607 | XP_018753723.1 | Peptidase M14 domain-containing protein |
| contig00014.g8377 | NC_031682.1 | FVEG_07653 | XP_018753777.1 | Peptide hydrolase (EC 3.4.-.-) |
| contig00014.g8381 | NC_031682.1 | FVEG_07656 | XP_018753780.1 | PA14 domain-containing protein |
| contig00014.g8382 | NC_031682.1 | FVEG_07657 | XP_018753781.1 | Tyrosinase copper-binding domain-containing protein |
| contig00014.g8389 | NC_031682.1 | FVEG_07663 | XP_018753789.1 | Uncharacterized protein |
| contig00014.g8390 | NC_031682.1 | FVEG_07664 | XP_018753790.1 | Ig-like domain-containing protein |
| contig00014.g8392 | NC_031682.1 | FVEG_07665 | XP_018753792.1 | Apple domain-containing protein |
| contig00014.g8405 | NC_031682.1 | FVEG_07677 | XP_018753807.1 | Peptidase S53 domain-containing protein |
| contig00014.g8406 | NC_031682.1 | FVEG_07678 | XP_018753808.1 | SGNH hydrolase-type esterase domain-containing protein |
| contig00014.g8415 | NC_031682.1 | FVEG_07688 | XP_018753818.1 | Aldose 1-epimerase |
| contig00014.g8417 | NC_031682.1 | FVEG_07690 | XP_018753820.1 | PA14 domain-containing protein |
| contig00014.g8423 | NC_031682.1 | FVEG_07695 | XP_018753826.1 | Hydrophobin |
| contig00014.g8431 | NC_031682.1 | FVEG_07702 | XP_018753834.1 | Beta-xylosidase C-terminal Concanavalin A-like domain |
| contig00014.g8443 | NC_031682.1 | FVEG_07712 | XP_018753846.1 | Carboxypeptidase (EC 3.4.16.-) |
| contig00014.g8447 | NC_031682.1 | FVEG_07716 | XP_018753850.1 | Alpha-galactosidase (EC 3.2.1.22) |
| contig00014.g8449 | NC_031682.1 | FVEG_07718 | XP_018753852.1 | FAD-binding PCMH-type domain-containing protein |
| contig00014.g8460 | NC_031682.1 | FVEG_07728 | XP_018753863.1 | CBM-cenC domain-containing protein |
| contig00014.g8462 | NC_031682.1 | FVEG_07730 | XP_018753865.1 | Carboxylic ester hydrolase (EC 3.1.1.-) |
| contig00014.g8481 | NC_031682.1 | FVEG_07747 | XP_018753883.1 | CBM-cenC domain-containing protein |
| contig00014.g8483 | NC_031682.1 | FVEG_07750 | XP_018753888.1 | Uncharacterized protein |
| contig00014.g8484 | NC_031682.1 | FVEG_07751 | XP_018753889.1 | Uncharacterized protein |
| contig00014.g8485 | NC_031682.1 | FVEG_07752 | XP_018753890.1 | Cyanovirin-N domain-containing protein |
| contig00014.g8487 | NC_031682.1 | FVEG_07753 | XP_018753892.1 | Glycoside hydrolase family 5 domain-containing protein |
| contig00014.g8496 | NC_031682.1 | FVEG_07763 | XP_018753905.1 | Fibronectin type-III domain-containing protein |
| contig00025.g11910 | NC_031682.1 | FVEG_13325 | XP_018761497.1 | AB hydrolase-1 domain-containing protein |
| contig00025.g11908 | NC_031682.1 | FVEG_13328 | XP_018761500.1 | CBM6 domain-containing protein |
| contig00025.g11895 | NC_031682.1 | FVEG_13339 | XP_018761514.1 | Carboxylic ester hydrolase (EC 3.1.1.-) |
| contig00025.g11890 | NC_031682.1 | FVEG_13343 | XP_018761519.1 | Beta-xylanase (EC 3.2.1.8) |
| contig00025.g11889 | NC_031682.1 | FVEG_13344 | XP_018761520.1 | lytic cellulose monooxygenase (C4-dehydrogenating) (EC 1.14.99.56) |
| contig00025.g11887 | NC_031682.1 | FVEG_13346 | XP_018761522.1 | Uncharacterized protein |
| contig00025.g11882 | NC_031682.1 | FVEG_13350 | XP_018761527.1 | Secreted protein |
| contig00025.g11877 | NC_031682.1 | FVEG_13356 | XP_018761535.1 | Uncharacterized protein |
| contig00025.g11874 | NC_031682.1 | FVEG_13359 | XP_018761538.1 | Carboxylic ester hydrolase (EC 3.1.1.-) |
| contig00025.g11869 | NC_031682.1 | FVEG_13364 | XP_018761545.1 | AB hydrolase-1 domain-containing protein |
| contig00025.g11841 | NC_031682.1 | FVEG_13391 | XP_018761574.1 | beta-glucosidase (EC 3.2.1.21) |
| contig00025.g11827 | NC_031682.1 | FVEG_13404 | XP_018761588.1 | Peptidase S8/S53 domain-containing protein |
| contig00025.g11826 | NC_031682.1 | FVEG_13405 | XP_018761589.1 | Laccase |
| contig00025.g11804 | NC_031682.1 | FVEG_13426 | XP_018761619.1 | Alpha-N-arabinofuranosidase |
| contig00025.g11797 | NC_031682.1 | FVEG_13433 | XP_018761626.1 | Peptidase M43 pregnancy-associated plasma-A domain |
| contig00025.g11792 | NC_031682.1 | FVEG_13439 | XP_018761631.1 | DUF7908 domain-containing protein |
| contig00025.g11781 | NC_031682.1 | FVEG_13450 | XP_018761643.1 | FAD-binding PCMH-type domain-containing protein |
| contig00025.g11777 | NC_031682.1 | FVEG_13454 | XP_018761648.1 | Chitinase |
| contig00025.g11775 | NC_031682.1 | FVEG_13456 | XP_018761650.1 | Cyanovirin-N domain-containing protein |
| contig00025.g11774 | NC_031682.1 | FVEG_13457 | XP_018761651.1 | Uncharacterized protein |
| contig00025.g11768 | NC_031682.1 | FVEG_13463 | XP_018761658.1 | Carbohydrate-binding module family 19 domain-containing protein |
| contig00025.g11760 | NC_031682.1 | FVEG_13471 | XP_018761669.1 | Cell wall mannoprotein |
| contig00025.g11747 | NC_031682.1 | FVEG_13483 | XP_018761683.1 | Fungal calcium binding protein domain-containing protein |
| contig00025.g11736 | NC_031682.1 | FVEG_13495 | XP_018761694.1 | Uncharacterized protein |
| contig00025.g11732 | NC_031682.1 | FVEG_13498 | XP_018761697.1 | Extracellular membrane protein CFEM domain-containing protein |
| contig00025.g11727 | NC_031682.1 | FVEG_13504 | XP_018761701.1 | lytic cellulose monooxygenase (C4-dehydrogenating) (EC 1.14.99.56) |
| contig00025.g11713 | NC_031682.1 | FVEG_13516 | XP_018761716.1 | endo-polygalacturonase (EC 3.2.1.15) (Pectinase) |
| contig00025.g11707 | NC_031682.1 | FVEG_13522 | XP_018761723.1 | Carboxylic ester hydrolase (EC 3.1.1.-) |
| contig00025.g11697 | NC_031682.1 | FVEG_13531 | XP_018761734.1 | D-arabinono-1,4-lactone oxidase (EC 1.1.3.37) |
| contig00025.g11694 | NC_031682.1 | FVEG_13534 | XP_018761737.1 | Cutinase |
| contig00025.g11677 | NC_031682.1 | FVEG_13553 | XP_018761760.1 | Arabinosidase |
| contig00025.g11676 | NC_031682.1 | FVEG_13554 | XP_018761761.1 | Uncharacterized protein |
| contig00025.g11675 | NC_031682.1 | FVEG_13555 | XP_018761762.1 | Purple acid phosphatase (EC 3.1.3.2) |
| contig00025.g11674 | NC_031682.1 | FVEG_13557 | XP_018761763.1 | Apple domain-containing protein |
| contig00025.g11663 | NC_031682.1 | FVEG_13565 | XP_018761774.1 | Uncharacterized protein |
| contig00025.g11652 | NC_031682.1 | FVEG_13578 | XP_018761787.1 | Glycosyl hydrolase family 30 beta sandwich domain-containing protein |
| contig00025.g11649 | NC_031682.1 | FVEG_13581 | XP_018761790.1 | Apple domain-containing protein |
| contig00025.g11645 | NC_031682.1 | FVEG_13584 | XP_018761794.1 | Phosphatidylinositol-specific phospholipase C X domain |
| contig00036.g13641 | NC_031682.1 | FVEG_13840 | XP_018762230.1 | Extracellular membrane protein CFEM domain-containing protein |
| contig00036.g13642 | NC_031682.1 | FVEG_13841 | XP_018762231.1 | Pel9A-like right handed beta-helix region domain-containing protein |
| contig00036.g13648 | NC_031682.1 | FVEG_13846 | XP_018762238.1 | Lysine-specific metallo-endopeptidase domain-containing protein |
| contig00036.g13649 | NC_031682.1 | FVEG_13848 | XP_018762240.1 | DUF4189 domain-containing protein |
| contig00036.g13650 | NC_031682.1 | FVEG_13849 | XP_018762242.1 | Glucoamylase (EC 3.2.1.3) (1,4-alpha-D-glucan glucohydrolase) |
| contig00036.g13654 | NC_031682.1 | FVEG_13853 | XP_018762246.1 | Apple domain-containing protein |
| contig00036.g13658 | NC_031682.1 | FVEG_13856 | XP_018762250.1 | Uncharacterized protein |
| contig00036.g13666 | NC_031682.1 | FVEG_13865 | XP_018762266.1 | Killer toxin Kp4 domain-containing protein |
| contig00036.g13672 | NC_031682.1 | FVEG_13872 | XP_018762276.1 | Uncharacterized protein |
| contig00036.g13678 | NC_031682.1 | FVEG_13877 | XP_018762282.1 | CBM-cenC domain-containing protein |
| contig00036.g13689 | NC_031682.1 | FVEG_13886 | XP_018762295.1 | Lysine-specific metallo-endopeptidase domain-containing protein |
| contig00036.g13690 | NC_031682.1 | FVEG_13887 | XP_018762296.1 | Methyl-accepting transducer domain-containing protein |
| contig00036.g13699 | NC_031682.1 | FVEG_13897 | XP_018762309.1 | Immune-responsive protein 1 |
| contig00036.g13705 | NC_031682.1 | FVEG_13902 | XP_018762316.1 | Uncharacterized protein |
| contig00036.g13713 | NC_031682.1 | FVEG_13909 | XP_018762324.1 | 6-phosphogluconolactonase |
| contig00036.g13718 | NC_031682.1 | FVEG_13912 | XP_018762329.1 | Apple domain-containing protein |
| contig00036.g13728 | NC_031682.1 | FVEG_13920 | XP_018762340.1 | Uncharacterized protein |
| contig00036.g13733 | NC_031682.1 | FVEG_13926 | XP_018762346.1 | Carboxylesterase type B domain-containing protein |
| contig00036.g13745 | NC_031682.1 | FVEG_13934 | XP_018762357.1 | Subtilisin |
| contig00036.g13758 | NC_031682.1 | FVEG_13947 | XP_018762374.1 | Uncharacterized protein |
| contig00036.g13759 | NC_031682.1 | FVEG_13948 | XP_018762375.1 | DJ-1/PfpI domain-containing protein |
| contig00036.g13760 | NC_031682.1 | FVEG_13949 | XP_018762376.1 | Epoxide hydrolase N-terminal domain-containing protein |
| contig00041.g14076 | NC_031682.1 | FVEG_13957 | XP_018762388.1 | LysM domain-containing protein |
| contig00041.g14068 | NC_031682.1 | FVEG_13964 | XP_018762396.1 | Killer toxin Kp4 domain-containing protein |
| contig00041.g14065 | NC_031682.1 | FVEG_13966 | XP_018762399.1 | Hypersensitive response-inducing protein |
| contig00041.g14044 | NC_031682.1 | FVEG_13986 | XP_018762423.1 | Uncharacterized protein |
| contig00014.g8246 | NC_031682.1 | FVEG_16133 | XP_018753594.1 | Apple domain-containing protein |
| contig00014.g8313 | NC_031682.1 | FVEG_16144 | XP_018753698.1 | Uncharacterized protein |
| contig00014.g8412 | NC_031682.1 | FVEG_16167 | XP_018753815.1 | DUF6604 domain-containing protein |
| contig00025.g11886 | NC_031682.1 | FVEG_17506 | XP_018761523.1 | Secreted protein |
| contig00025.g11793 | NC_031682.1 | FVEG_17524 | XP_018761630.1 | SGNH hydrolase-type esterase domain-containing protein |
| contig00025.g11731 | NC_031682.1 | FVEG_17533 | XP_018761698.1 | alpha,alpha-trehalase (EC 3.2.1.28) |
| contig00025.g11684 | NC_031682.1 | FVEG_17546 | XP_018761748.1 | chitinase (EC 3.2.1.14) |
| contig00025.g11683 | NC_031682.1 | FVEG_17547 | XP_018761749.1 | Glucan 1,3-beta-glucosidase |
| contig00036.g13681 | NC_031682.1 | FVEG_17643 | XP_018762286.1 | CBM-cenC domain-containing protein |
| contig00013.g8017 | NC_031683.1 | FVEG_11018^a^ | XP_018758417.1 | Secreted protein |
| contig00009.g5850 | NC_031683.1 | FVEG_09988 | XP_018757050.1 | DUF7908 domain-containing protein |
| contig00009.g5860 | NC_031683.1 | FVEG_09998 | XP_018757063.1 | Uncharacterized protein |
| contig00009.g5862 | NC_031683.1 | FVEG_10000 | XP_018757066.1 | Early nodulin 75 |
| contig00009.g5901 | NC_031683.1 | FVEG_10031 | XP_018757105.1 | Ecp2 effector protein domain-containing protein |
| contig00009.g5902 | NC_031683.1 | FVEG_10032 | XP_018757106.1 | alpha-1,2-Mannosidase (EC 3.2.1.-) |
| contig00009.g5926 | NC_031683.1 | FVEG_10058 | XP_018757132.1 | Uncharacterized protein |
| contig00009.g5928 | NC_031683.1 | FVEG_10059 | XP_018757134.1 | Ubiquitin 3 binding protein But2 C-terminal domain-containing protein |
| contig00009.g5937 | NC_031683.1 | FVEG_10071 | XP_018757147.1 | Phospholipase A2 |
| contig00009.g5938 | NC_031683.1 | FVEG_10072 | XP_018757148.1 | Carboxypeptidase Y inhibitor |
| contig00009.g5942 | NC_031683.1 | FVEG_10077 | XP_018757152.1 | Protein rds1 |
| contig00009.g5948 | NC_031683.1 | FVEG_10083 | XP_018757157.1 | Levanase |
| contig00009.g5958 | NC_031683.1 | FVEG_10092 | XP_018757168.1 | AB hydrolase-1 domain-containing protein |
| contig00009.g5963 | NC_031683.1 | FVEG_10098 | XP_018757172.1 | Uncharacterized protein |
| contig00009.g5965 | NC_031683.1 | FVEG_10100 | XP_018757174.1 | CBM-cenC domain-containing protein |
| contig00009.g5966 | NC_031683.1 | FVEG_10101 | XP_018757175.1 | pectate lyase (EC 4.2.2.2) |
| contig00009.g5967 | NC_031683.1 | FVEG_10102 | XP_018757176.1 | Pectate lyase (EC 4.2.2.2) |
| contig00009.g5968 | NC_031683.1 | FVEG_10103 | XP_018757177.1 | Isochorismatase-like domain-containing protein |
| contig00009.g5976 | NC_031683.1 | FVEG_10111 | XP_018757186.1 | FAD-binding PCMH-type domain-containing protein |
| contig00009.g5980 | NC_031683.1 | FVEG_10115 | XP_018757190.1 | Peptidase M20 dimerisation domain-containing protein |
| contig00009.g5981 | NC_031683.1 | FVEG_10116 | XP_018757191.1 | lytic cellulose monooxygenase (C4-dehydrogenating) (EC 1.14.99.56) |
| contig00009.g5982 | NC_031683.1 | FVEG_10117 | XP_018757192.1 | Multiple epidermal growth factor-like domains protein 6 |
| contig00009.g5985 | NC_031683.1 | FVEG_10119 | XP_018757195.1 | Putative peptidase domain-containing protein |
| contig00009.g6013 | NC_031683.1 | FVEG_10146 | XP_018757228.1 | Endonuclease/exonuclease/phosphatase domain-containing protein |
| contig00009.g6017 | NC_031683.1 | FVEG_10149 | XP_018757231.1 | FAD-binding PCMH-type domain-containing protein |
| contig00009.g6064 | NC_031683.1 | FVEG_10189 | XP_018757286.1 | Amidase domain-containing protein |
| contig00009.g6111 | NC_031683.1 | FVEG_10241 | XP_018757355.1 | Expansin-like EG45 domain-containing protein |
| contig00009.g6154 | NC_031683.1 | FVEG_10284 | XP_018757417.1 | Carboxypeptidase D |
| contig00009.g6203 | NC_031683.1 | FVEG_10333 | XP_018757517.1 | Chitin binding protein |
| contig00009.g6234 | NC_031683.1 | FVEG_10364 | XP_018757562.1 | Allergen Asp f 4 |
| contig00009.g6278 | NC_031683.1 | FVEG_10404 | XP_018757627.1 | AA1-like domain-containing protein |
| contig00009.g6306 | NC_031683.1 | FVEG_10428 | XP_018757665.1 | Secreted protein |
| contig00009.g6323 | NC_031683.1 | FVEG_10446 | XP_018757689.1 | DUF1237 domain protein |
| contig00013.g8068 | NC_031683.1 | FVEG_10964 | XP_018758347.1 | Uncharacterized protein |
| contig00013.g8054 | NC_031683.1 | FVEG_10979 | XP_018758369.1 | Amine oxidase domain-containing protein |
| contig00013.g8024 | NC_031683.1 | FVEG_11011 | XP_018758408.1 | PA14 domain-containing protein |
| contig00002.g2056 | NC_031683.1 | FVEG_11018 | XP_018758417.1 | Secreted protein |
| contig00013.g8003 | NC_031683.1 | FVEG_11031 | XP_018758432.1 | AB hydrolase-1 domain-containing protein |
| contig00013.g7976 | NC_031683.1 | FVEG_11053 | XP_018758460.1 | Uncharacterized protein |
| contig00013.g7969 | NC_031683.1 | FVEG_11061 | XP_018758469.1 | carbonic anhydrase (EC 4.2.1.1) |
| contig00013.g7965 | NC_031683.1 | FVEG_11065 | XP_018758473.1 | Small secreted protein |
| contig00013.g7942 | NC_031683.1 | FVEG_11083 | XP_018758496.1 | Secreted aspartic protease FUS4 (EC 3.4.23.-) |
| contig00013.g7939 | NC_031683.1 | FVEG_11087 | XP_018758500.1 | Asl1-like glycosyl hydrolase catalytic domain-containing protein |
| contig00013.g7934 | NC_031683.1 | FVEG_11094 | XP_018758506.1 | Phosphoglycerate mutase |
| contig00013.g7924 | NC_031683.1 | FVEG_11103 | XP_018758520.1 | Uncharacterized protein |
| contig00013.g7914 | NC_031683.1 | FVEG_11115 | XP_018758533.1 | CBM-cenC domain-containing protein |
| contig00013.g7902 | NC_031683.1 | FVEG_11125 | XP_018758546.1 | 1-alkyl-2-acetylglycerophosphocholine esterase (EC 3.1.1.47) |
| contig00030.g12773 | NC_031683.1 | FVEG_11126 | XP_018758547.1 | Jacalin-type lectin domain-containing protein |
| contig00013.g7900 | NC_031683.1 | FVEG_11127 | XP_018758548.1 | Carboxypeptidase (EC 3.4.16.-) |
| contig00013.g7890 | NC_031683.1 | FVEG_11137 | XP_018758560.1 | Tyrosinase copper-binding domain-containing protein |
| contig00013.g7874 | NC_031683.1 | FVEG_11151 | XP_018758577.1 | Ecp2 effector protein domain-containing protein |
| contig00013.g7854 | NC_031683.1 | FVEG_11173 | XP_018758606.1 | Cerato-platanin |
| contig00013.g7836 | NC_031683.1 | FVEG_11197 | XP_018758643.1 | RlpA-like protein double-psi beta-barrel domain-containing protein |
| contig00013.g7835 | NC_031683.1 | FVEG_11198 | XP_018758644.1 | FAD-binding PCMH-type domain-containing protein |
| contig00013.g7811 | NC_031683.1 | FVEG_11221 | XP_018758672.1 | Peroxidase (EC 1.11.1.-) |
| contig00013.g7806 | NC_031683.1 | FVEG_11228 | XP_018758677.1 | pectinesterase (EC 3.1.1.11) (Pectin methylesterase A) |
| contig00013.g7802 | NC_031683.1 | FVEG_11232 | XP_018758681.1 | Apple domain-containing protein |
| contig00013.g7801 | NC_031683.1 | FVEG_11233 | XP_018758682.1 | Pectate lyase domain-containing protein |
| contig00013.g7763 | NC_031683.1 | FVEG_11273 | XP_018758736.1 | Phytocyanin domain-containing protein |
| contig00013.g7753 | NC_031683.1 | FVEG_11284 | XP_018758755.1 | Late sexual development protein |
| contig00013.g7718 | NC_031683.1 | FVEG_11317 | XP_018758818.1 | Peptidase A1 domain-containing protein |
| contig00009.g5962 | NC_031683.1 | FVEG_16722 | XP_018757171.1 | Beta-galactosidase (EC 3.2.1.23) |
| contig00009.g6072 | NC_031683.1 | FVEG_16752 | XP_018757295.1 | Proteophosphoglycan ppg4 |
| contig00013.g8010 | NC_031683.1 | FVEG_16950 | XP_018758425.1 | Uncharacterized protein |
| contig00005.g3700 | NC_031684.1 | FVEG_08301 | XP_018754781.1 | GH16 domain-containing protein |
| contig00005.g3707 | NC_031684.1 | FVEG_08305 | XP_018754791.1 | Prolyl 4-hydroxylase alpha subunit domain-containing protein |
| contig00005.g3709 | NC_031684.1 | FVEG_08309 | XP_018754795.1 | Tyrosinase copper-binding domain-containing protein |
| contig00005.g3738 | NC_031684.1 | FVEG_08331 | XP_018754818.1 | GEgh 16 protein |
| contig00005.g3754 | NC_031684.1 | FVEG_08344 | XP_018754835.1 | CBM6 domain-containing protein |
| contig00005.g3755 | NC_031684.1 | FVEG_08345 | XP_018754836.1 | Murein transglycosylase |
| contig00005.g3757 | NC_031684.1 | FVEG_08347 | XP_018754838.1 | Uncharacterized protein |
| contig00005.g3758 | NC_031684.1 | FVEG_08348 | XP_018754839.1 | Ecp2 effector protein domain-containing protein |
| contig00005.g3759 | NC_031684.1 | FVEG_08349 | XP_018754840.1 | Ecp2 effector protein domain-containing protein |
| contig00005.g3766 | NC_031684.1 | FVEG_08355 | XP_018754849.1 | Alpha-L-rhamnosidase C-terminal domain-containing protein |
| contig00005.g3786 | NC_031684.1 | FVEG_08373 | XP_018754870.1 | Aspergillopepsin-2 |
| contig00005.g3803 | NC_031684.1 | FVEG_08386 | XP_018754890.1 | Zonadhesin |
| contig00005.g3810 | NC_031684.1 | FVEG_08392 | XP_018754900.1 | WSC domain-containing protein |
| contig00005.g3815 | NC_031684.1 | FVEG_08397 | XP_018754905.1 | CBM-cenC domain-containing protein |
| contig00005.g3821 | NC_031684.1 | FVEG_08403 | XP_018754911.1 | Peptidase A1 domain-containing protein |
| contig00005.g3832 | NC_031684.1 | FVEG_08413 | XP_018754925.1 | Glycoside hydrolase family 39 protein |
| contig00005.g3840 | NC_031684.1 | FVEG_08421 | XP_018754934.1 | Endo-arabinase |
| contig00005.g3841 | NC_031684.1 | FVEG_08422 | XP_018754937.1 | FAD-binding PCMH-type domain-containing protein |
| contig00005.g3848 | NC_031684.1 | FVEG_08430 | XP_018754947.1 | Cytochrome P450 oxidoreductase |
| contig00005.g3853 | NC_031684.1 | FVEG_08435 | XP_018754952.1 | CBM-cenC domain-containing protein |
| contig00005.g3872 | NC_031684.1 | FVEG_08451 | XP_018754972.1 | endo-polygalacturonase (EC 3.2.1.15) (Pectinase) |
| contig00005.g3879 | NC_031684.1 | FVEG_08459 | XP_018754982.1 | Uncharacterized protein |
| contig00005.g3881 | NC_031684.1 | FVEG_08461 | XP_018754984.1 | Carboxylic ester hydrolase (EC 3.1.1.-) |
| contig00005.g3890 | NC_031684.1 | FVEG_08470 | XP_018754998.1 | chitinase (EC 3.2.1.14) |
| contig00005.g3933 | NC_031684.1 | FVEG_08511 | XP_018755046.1 | Glycerol-3-phosphate dehydrogenase (EC 1.1.5.3) |
| contig00005.g3939 | NC_031684.1 | FVEG_08518 | XP_018755055.1 | Carboxylic ester hydrolase (EC 3.1.1.-) |
| contig00005.g3967 | NC_031684.1 | FVEG_08546 | XP_018755087.1 | Acriflavine sensitivity control protein acr-2 |
| contig00005.g3977 | NC_031684.1 | FVEG_08555 | XP_018755097.1 | Galactose oxidase (EC 1.1.3.9) |
| contig00005.g3980 | NC_031684.1 | FVEG_08558 | XP_018755100.1 | Secreted protein CSS2 C-terminal domain-containing protein |
| contig00005.g4007 | NC_031684.1 | FVEG_08586 | XP_018755138.1 | Fungal N-terminal domain-containing protein |
| contig00005.g4020 | NC_031684.1 | FVEG_08596 | XP_018755151.1 | TNT domain-containing protein |
| contig00005.g4030 | NC_031684.1 | FVEG_08605 | XP_018755161.1 | Beta-xylosidase C-terminal Concanavalin A-like domain |
| contig00005.g4066 | NC_031684.1 | FVEG_08638 | XP_018755204.1 | IDI-2 |
| contig00005.g4079 | NC_031684.1 | FVEG_08650 | XP_018755218.1 | beta-glucosidase (EC 3.2.1.21) |
| contig00005.g4087 | NC_031684.1 | FVEG_08658 | XP_018755227.1 | Phosphorylcholine phosphatase |
| contig00005.g4088 | NC_031684.1 | FVEG_08659 | XP_018755228.1 | beta-galactosidase (EC 3.2.1.23) |
| contig00005.g4110 | NC_031684.1 | FVEG_08679 | XP_018755253.1 | Alkaline proteinase |
| contig00005.g4117 | NC_031684.1 | FVEG_08686 | XP_018755260.1 | SCP domain-containing protein |
| contig00005.g4119 | NC_031684.1 | FVEG_08688 | XP_018755264.1 | Uncharacterized protein |
| contig00005.g4121 | NC_031684.1 | FVEG_08690 | XP_018755265.1 | FAD-binding PCMH-type domain-containing protein |
| contig00005.g4124 | NC_031684.1 | FVEG_08694 | XP_018755269.1 | FAD-binding PCMH-type domain-containing protein |
| contig00005.g4132 | NC_031684.1 | FVEG_08702 | XP_018755278.1 | Glucose-methanol-choline oxidoreductase |
| contig00005.g4137 | NC_031684.1 | FVEG_08707 | XP_018755284.1 | Glucose-methanol-choline oxidoreductase |
| contig00005.g4152 | NC_031684.1 | FVEG_08723 | XP_018755302.1 | GH16 domain-containing protein |
| contig00005.g4167 | NC_031684.1 | FVEG_08734 | XP_018755318.1 | Pectate lyase (EC 4.2.2.2) |
| contig00005.g4178 | NC_031684.1 | FVEG_08746 | XP_018755331.1 | Uncharacterized protein |
| contig00005.g4180 | NC_031684.1 | FVEG_08748 | XP_018755334.1 | Uncharacterized protein |
| contig00005.g4191 | NC_031684.1 | FVEG_08757 | XP_018755347.1 | Heme haloperoxidase family profile domain-containing protein |
| contig00005.g4193 | NC_031684.1 | FVEG_08758 | XP_018755349.1 | Apple domain-containing protein |
| contig00005.g4217 | NC_031684.1 | FVEG_08780 | XP_018755379.1 | Carboxypeptidase M14A |
| contig00005.g4220 | NC_031684.1 | FVEG_08782 | XP_018755382.1 | TNT domain-containing protein |
| contig00005.g4221 | NC_031684.1 | FVEG_08783 | XP_018755383.1 | lytic cellulose monooxygenase (C4-dehydrogenating) (EC 1.14.99.56) |
| contig00005.g4223 | NC_031684.1 | FVEG_08785 | XP_018755385.1 | Carboxylic ester hydrolase (EC 3.1.1.-) |
| contig00005.g4240 | NC_031684.1 | FVEG_08802 | XP_018755408.1 | Ecp2 effector protein domain-containing protein |
| contig00005.g4246 | NC_031684.1 | FVEG_08808 | XP_018755415.1 | Uncharacterized protein |
| contig00008.g5820 | NC_031684.1 | FVEG_08821 | XP_018755431.1 | lytic cellulose monooxygenase (C4-dehydrogenating) (EC 1.14.99.56) |
| contig00008.g5817 | NC_031684.1 | FVEG_08824 | XP_018755434.1 | Alpha-glucuronidase (EC 3.2.1.139) |
| contig00008.g5816 | NC_031684.1 | FVEG_08825 | XP_018755435.1 | Glucanase (EC 3.2.1.-) |
| contig00008.g5812 | NC_031684.1 | FVEG_08828 | XP_018755440.1 | Endo-1,4-beta-xylanase (EC 3.2.1.8) |
| contig00008.g5807 | NC_031684.1 | FVEG_08833 | XP_018755445.1 | SGNH hydrolase-type esterase domain-containing protein |
| contig00008.g5804 | NC_031684.1 | FVEG_08836 | XP_018755449.1 | Beta-xylosidase C-terminal Concanavalin A-like domain |
| contig00008.g5799 | NC_031684.1 | FVEG_08840 | XP_018755454.1 | Uncharacterized protein |
| contig00008.g5785 | NC_031684.1 | FVEG_08854 | XP_018755470.1 | Sialidase domain-containing protein |
| contig00008.g5780 | NC_031684.1 | FVEG_08858 | XP_018755475.1 | Alginate lyase domain-containing protein |
| contig00008.g5770 | NC_031684.1 | FVEG_08867 | XP_018755486.1 | Mutanase |
| contig00008.g5766 | NC_031684.1 | FVEG_08870 | XP_018755490.1 | Carboxylic ester hydrolase (EC 3.1.1.-) |
| contig00008.g5764 | NC_031684.1 | FVEG_08871 | XP_018755492.1 | CFEM domain-containing protein |
| contig00008.g5763 | NC_031684.1 | FVEG_08872 | XP_018755493.1 | Uncharacterized protein |
| contig00008.g5743 | NC_031684.1 | FVEG_08891 | XP_018755518.1 | Galactose oxidase (EC 1.1.3.9) (Sialidase-1) |
| contig00008.g5740 | NC_031684.1 | FVEG_08895 | XP_018755523.1 | Uncharacterized protein |
| contig00008.g5739 | NC_031684.1 | FVEG_08896 | XP_018755524.1 | Uncharacterized protein |
| contig00033.g13343 | NC_031684.1 | FVEG_13589 | XP_018761800.1 | Uncharacterized protein |
| contig00033.g13326 | NC_031684.1 | FVEG_13605 | XP_018761822.1 | Peptidase A1 domain-containing protein |
| contig00033.g13316 | NC_031684.1 | FVEG_13614 | XP_018761833.1 | PRC1-carboxypeptidase y, serine-type protease |
| contig00033.g13302 | NC_031684.1 | FVEG_13630 | XP_018761850.1 | Extracellular metalloproteinase (EC 3.4.24.-) (Fungalysin) |
| contig00033.g13295 | NC_031684.1 | FVEG_13638 | XP_018761858.1 | cutinase (EC 3.1.1.74) |
| contig00033.g13290 | NC_031684.1 | FVEG_13642 | XP_018761864.1 | Small secreted protein |
| contig00033.g13289 | NC_031684.1 | FVEG_13643 | XP_018761865.1 | Uncharacterized protein |
| contig00033.g13276 | NC_031684.1 | FVEG_13655 | XP_018761883.1 | EthD domain-containing protein |
| contig00033.g13274 | NC_031684.1 | FVEG_13657 | XP_018761885.1 | glucan endo-1,3-beta-D-glucosidase (EC 3.2.1.39) |
| contig00033.g13263 | NC_031684.1 | FVEG_13667 | XP_018761906.1 | Uncharacterized protein |
| contig00033.g13262 | NC_031684.1 | FVEG_13668 | XP_018761907.1 | Ecp2 effector protein domain-containing protein |
| contig00033.g13260 | NC_031684.1 | FVEG_13669 | XP_018761909.1 | Small secreted protein |
| contig00033.g13255 | NC_031684.1 | FVEG_13674 | XP_018761914.1 | Apple domain-containing protein |
| contig00033.g13244 | NC_031684.1 | FVEG_13685 | XP_018761925.1 | Acetylornithine deacetylase |
| contig00033.g13237 | NC_031684.1 | FVEG_13692 | XP_018761933.1 | DUF985 domain-containing protein |
| contig00033.g13221 | NC_031684.1 | FVEG_13708 | XP_018761950.1 | Uncharacterized protein |
| contig00033.g13217 | NC_031684.1 | FVEG_13711 | XP_018761954.1 | pectin lyase (EC 4.2.2.10) |
| contig00033.g13201 | NC_031684.1 | FVEG_13728 | XP_018761969.1 | Ig-like domain-containing protein |
| contig00033.g13199 | NC_031684.1 | FVEG_13730 | XP_018761971.1 | NADP-dependent oxidoreductase domain-containing protein |
| contig00033.g13193 | NC_031684.1 | FVEG_13736 | XP_018761979.1 | Right handed beta helix domain-containing protein |
| contig00033.g13172 | NC_031684.1 | FVEG_13757 | XP_018762001.1 | SGNH hydrolase-type esterase domain-containing protein |
| contig00038.g13872 | NC_031684.1 | FVEG_13759 | XP_018762003.1 | Probable glucan endo-1,3-beta-glucosidase eglC (EC 3.2.1.39) |
| contig00038.g13879 | NC_031684.1 | FVEG_13765 | XP_018762011.1 | LysM domain-containing protein |
| contig00038.g13880 | NC_031684.1 | FVEG_13766 | XP_018762012.1 | LysM domain-containing protein |
| contig00038.g13889 | NC_031684.1 | FVEG_13776 | XP_018762025.1 | Tyrosinase copper-binding domain-containing protein |
| contig00038.g13901 | NC_031684.1 | FVEG_13788 | XP_018762038.1 | Apple domain-containing protein |
| contig00038.g13923 | NC_031684.1 | FVEG_13811 | XP_018762061.1 | Alkaline protease 1 |
| contig00038.g13925 | NC_031684.1 | FVEG_13813 | XP_018762063.1 | alpha-galactosidase (EC 3.2.1.22) |
| contig00038.g13931 | NC_031684.1 | FVEG_13819 | XP_018762072.1 | NodB homology domain-containing protein |
| contig00038.g13935 | NC_031684.1 | FVEG_13823 | XP_018762078.1 | Carboxylic ester hydrolase (EC 3.1.1.-) |
| contig00038.g13940 | NC_031684.1 | FVEG_13826 | XP_018762083.1 | SMP-30/Gluconolactonase/LRE-like region domain-containing protein |
| contig00038.g13946 | NC_031684.1 | FVEG_13832 | XP_018762091.1 | DUF7872 domain-containing protein |
| contig00038.g13947 | NC_031684.1 | FVEG_13833 | XP_018762092.1 | Uncharacterized protein |
| contig00038.g13948 | NC_031684.1 | FVEG_13834 | XP_018762093.1 | Uncharacterized protein |
| contig00005.g3779 | NC_031684.1 | FVEG_16301 | XP_018754862.1 | Ecp2 effector protein domain-containing protein |
| contig00005.g3950 | NC_031684.1 | FVEG_16349 | XP_018755067.1 | Uncharacterized protein |
| contig00005.g4033 | NC_031684.1 | FVEG_16373 | XP_018755165.1 | WSC domain-containing protein |
| contig00005.g4050 | NC_031684.1 | FVEG_16379 | XP_018755184.1 | Apple domain-containing protein |
| contig00005.g4091 | NC_031684.1 | FVEG_16393 | XP_018755232.1 | Ig-like domain-containing protein |
| contig00005.g4098 | NC_031684.1 | FVEG_16396 | XP_018755241.1 | Beta-galactosidase |
| contig00005.g4111 | NC_031684.1 | FVEG_16398 | XP_018755254.1 | Tenascin X |
| contig00005.g4162 | NC_031684.1 | FVEG_16409 | XP_018755313.1 | FAD-binding PCMH-type domain-containing protein |
| contig00005.g4238 | NC_031684.1 | FVEG_16443 | XP_018755405.1 | Uncharacterized protein |
| contig00008.g5791 | NC_031684.1 | FVEG_16455 | XP_018755462.1 | Uncharacterized protein |
| contig00038.g13878 | NC_031684.1 | FVEG_17600 | XP_018762010.1 | chitinase (EC 3.2.1.14) |
| contig00038.g13929 | NC_031684.1 | FVEG_17613 | XP_018762070.1 | Exo-1,4-beta-D-glucosaminidase |
| contig00038.g13937 | NC_031684.1 | FVEG_17615 | XP_018762080.1 | Apple domain-containing protein |
| contig00022.g10881 | NC_031685.1 | FVEG_10538 | XP_018757812.1 | AttH domain-containing protein |
| contig00022.g10885 | NC_031685.1 | FVEG_10542 | XP_018757816.1 | Cupin type-1 domain-containing protein |
| contig00022.g10887 | NC_031685.1 | FVEG_10544 | XP_018757818.1 | Ig-like domain-containing protein |
| contig00022.g10907 | NC_031685.1 | FVEG_10558 | XP_018757835.1 | Peptide hydrolase (EC 3.4.-.-) |
| contig00022.g10921 | NC_031685.1 | FVEG_10576 | XP_018757862.1 | Uncharacterized protein |
| contig00022.g10924 | NC_031685.1 | FVEG_10579 | XP_018757865.1 | Fungal calcium binding protein domain-containing protein |
| contig00022.g10928 | NC_031685.1 | FVEG_10582 | XP_018757870.1 | Heme haloperoxidase family profile domain-containing protein |
| contig00022.g10930 | NC_031685.1 | FVEG_10584 | XP_018757872.1 | Choline dehydrogenase |
| contig00022.g10976 | NC_031685.1 | FVEG_10600 | XP_018757894.1 | Ecp2 effector protein domain-containing protein |
| contig00022.g10974 | NC_031685.1 | FVEG_10602 | XP_018757896.1 | Carboxypeptidase M14B (Carboxypeptidase MCPB) |
| contig00022.g10971 | NC_031685.1 | FVEG_10605 | XP_018757899.1 | Alpha-L-rhamnosidase six-hairpin glycosidase domain |
| contig00022.g10951 | NC_031685.1 | FVEG_10624 | XP_018757923.1 | Arabinan endo-1,5-alpha-L-arabinosidase (EC 3.2.1.99) |
| contig00022.g10950 | NC_031685.1 | FVEG_10625 | XP_018757924.1 | Uncharacterized protein |
| contig00022.g10992 | NC_031685.1 | FVEG_10647 | XP_018757949.1 | FAD-binding domain-containing protein |
| contig00022.g11020 | NC_031685.1 | FVEG_10673 | XP_018757981.1 | Carboxylic ester hydrolase (EC 3.1.1.-) |
| contig00022.g11086 | NC_031685.1 | FVEG_10735 | XP_018758065.1 | BNR/Asp-box repeat domain protein |
| contig00022.g11088 | NC_031685.1 | FVEG_10738 | XP_018758069.1 | Tat pathway signal sequence domain protein |
| contig00022.g11090 | NC_031685.1 | FVEG_10740 | XP_018758071.1 | Beta-lactamase-related domain-containing protein |
| contig00022.g11095 | NC_031685.1 | FVEG_10744 | XP_018758077.1 | Alpha-L-arabinofuranosidase II |
| contig00022.g11098 | NC_031685.1 | FVEG_10747 | XP_018758080.1 | Ubiquitin 3 binding protein But2 C-terminal domain-containing protein |
| contig00028.g12312 | NC_031685.1 | FVEG_10761 | XP_018758099.1 | Ecp2 effector protein domain-containing protein |
| contig00028.g12315 | NC_031685.1 | FVEG_10764 | XP_018758102.1 | Multiprotein-bridging factor 1 |
| contig00028.g12316 | NC_031685.1 | FVEG_10765 | XP_018758103.1 | Uncharacterized protein |
| contig00028.g12322 | NC_031685.1 | FVEG_10770 | XP_018758110.1 | Uncharacterized protein |
| contig00028.g12347 | NC_031685.1 | FVEG_10795 | XP_018758142.1 | Pectinesterase (EC 3.1.1.11) |
| contig00028.g12348 | NC_031685.1 | FVEG_10796 | XP_018758143.1 | Uncharacterized protein |
| contig00028.g12351 | NC_031685.1 | FVEG_10798 | XP_018758146.1 | Peptidase S9 prolyl oligopeptidase catalytic domain-containing protein |
| contig00028.g12363 | NC_031685.1 | FVEG_10812 | XP_018758160.1 | CBM-cenC domain-containing protein |
| contig00028.g12365 | NC_031685.1 | FVEG_10814 | XP_018758162.1 | Pel9A-like right handed beta-helix region domain-containing protein |
| contig00028.g12387 | NC_031685.1 | FVEG_10833 | XP_018758187.1 | SCP domain-containing protein |
| contig00028.g12400 | NC_031685.1 | FVEG_10845 | XP_018758202.1 | Right handed beta helix domain-containing protein |
| contig00028.g12402 | NC_031685.1 | FVEG_10848 | XP_018758205.1 | Antigenic cell wall galactomannoprotein |
| contig00028.g12413 | NC_031685.1 | FVEG_10860 | XP_018758220.1 | Uncharacterized protein |
| contig00028.g12415 | NC_031685.1 | FVEG_10862 | XP_018758222.1 | Pectate lyase (EC 4.2.2.2) |
| contig00028.g12416 | NC_031685.1 | FVEG_10863 | XP_018758223.1 | Cuticle-degrading protease |
| contig00028.g12419 | NC_031685.1 | FVEG_10867 | XP_018758228.1 | FAD-binding PCMH-type domain-containing protein |
| contig00028.g12421 | NC_031685.1 | FVEG_10869 | XP_018758230.1 | Uncharacterized protein |
| contig00028.g12446 | NC_031685.1 | FVEG_10896 | XP_018758260.1 | Cellulose-binding protein |
| contig00028.g12447 | NC_031685.1 | FVEG_10897 | XP_018758261.1 | Glucanase (EC 3.2.1.-) |
| contig00028.g12452 | NC_031685.1 | FVEG_10901 | XP_018758269.1 | Carboxylic ester hydrolase (EC 3.1.1.-) |
| contig00028.g12458 | NC_031685.1 | FVEG_10907 | XP_018758275.1 | N1-acetylpolyamine oxidase |
| contig00028.g12476 | NC_031685.1 | FVEG_10918 | XP_018758291.1 | Secreted protein |
| contig00028.g12485 | NC_031685.1 | FVEG_10926 | XP_018758302.1 | Acid phosphatase |
| contig00028.g12487 | NC_031685.1 | FVEG_10928 | XP_018758305.1 | DUF7029 domain-containing protein |
| contig00028.g12495 | NC_031685.1 | FVEG_10936 | XP_018758314.1 | ribonuclease T2 (EC 4.6.1.19) |
| contig00028.g12501 | NC_031685.1 | FVEG_10941 | XP_018758320.1 | Necrosis inducing protein (NPP1) |
| contig00028.g12518 | NC_031685.1 | FVEG_10956 | XP_018758339.1 | ShKT domain-containing protein |
| contig00028.g12524 | NC_031685.1 | FVEG_10963 | XP_018758346.1 | Inositol polyphosphate-related phosphatase domain |
| contig00037.g13861 | NC_031685.1 | FVEG_12823 | XP_018760862.1 | Amine oxidase (EC 1.4.3.-) |
| contig00037.g13842 | NC_031685.1 | FVEG_12840 | XP_018760885.1 | GH16 domain-containing protein |
| contig00037.g13824 | NC_031685.1 | FVEG_12856 | XP_018760913.1 | Spore coat protein SP96 |
| contig00037.g13802 | NC_031685.1 | FVEG_12878 | XP_018760946.1 | Carboxypeptidase (EC 3.4.16.-) |
| contig00037.g13801 | NC_031685.1 | FVEG_12879 | XP_018760947.1 | Uncharacterized protein |
| contig00037.g13792 | NC_031685.1 | FVEG_12888 | XP_018760958.1 | Catalase-peroxidase (CP) (EC 1.11.1.21) (Peroxidase/catalase) |
| contig00037.g13776 | NC_031685.1 | FVEG_12905 | XP_018760980.1 | Uncharacterized protein |
| contig00037.g13769 | NC_031685.1 | FVEG_12910 | XP_018760988.1 | Gluconolactonase |
| contig00037.g13766 | NC_031685.1 | FVEG_12913 | XP_018760991.1 | Endoglycoceramidase |
| contig00034.g13348 | NC_031685.1 | FVEG_12922 | XP_018761002.1 | Uncharacterized protein |
| contig00034.g13355 | NC_031685.1 | FVEG_12928 | XP_018761009.1 | Acid phosphatase |
| contig00034.g13357 | NC_031685.1 | FVEG_12930 | XP_018761011.1 | cellulase (EC 3.2.1.4) |
| contig00034.g13361 | NC_031685.1 | FVEG_12936 | XP_018761017.1 | Tyrosinase copper-binding domain |
| contig00004.g3510 | NC_031685.1 | FVEG_12952 | XP_018761036.1 | Alpha-L-arabinofuranosidase (EC 3.2.1.55) |
| contig00034.g13383 | NC_031685.1 | FVEG_12957 | XP_018761041.1 | Alpha-amylase |
| contig00034.g13390 | NC_031685.1 | FVEG_12964 | XP_018761049.1 | Peptidase M43 pregnancy-associated plasma-A domain |
| contig00034.g13392 | NC_031685.1 | FVEG_12965 | XP_018761050.1 | GH16 domain-containing protein |
| contig00034.g13414 | NC_031685.1 | FVEG_12983 | XP_018761075.1 | Peptidase M6-like domain-containing protein |
| contig00034.g13416 | NC_031685.1 | FVEG_12984 | XP_018761077.1 | Apple domain-containing protein |
| contig00034.g13439 | NC_031685.1 | FVEG_13004 | XP_018761101.1 | Uncharacterized protein |
| contig00034.g13443 | NC_031685.1 | FVEG_13008 | XP_018761105.1 | Gylcosyl hydrolase 115 C-terminal domain |
| contig00034.g13492 | NC_031685.1 | FVEG_13055 | XP_018761171.1 | Beta-glucosidase cel3A (EC 3.2.1.21) |
| contig00034.g13515 | NC_031685.1 | FVEG_13080 | XP_018761198.1 | GEgh 16 protein |
| contig00022.g10823 | NC_031685.1 | FVEG_16810 | XP_018757742.1 | Carboxylic ester hydrolase (EC 3.1.1.-) |
| contig00022.g10864 | NC_031685.1 | FVEG_16817 | XP_018757790.1 | Antifungal protein |
| contig00022.g10866 | NC_031685.1 | FVEG_16818 | XP_018757792.1 | Secreted protein |
| contig00022.g10899 | NC_031685.1 | FVEG_16828 | XP_018757832.1 | Extracellular membrane protein CFEM domain |
| contig00022.g11027 | NC_031685.1 | FVEG_16861 | XP_018757988.1 | Uncharacterized protein |
| contig00034.g13413 | NC_031685.1 | FVEG_17415 | XP_018761074.1 | Uncharacterized protein |
| contig00034.g13415 | NC_031685.1 | FVEG_17416 | XP_018761076.1 | Uncharacterized protein |
| contig00034.g13425 | NC_031685.1 | FVEG_17418 | XP_018761086.1 | Uncharacterized protein |
| contig00034.g13378 | NC_031685.1 | . | XP_018761036.1 | alpha-N-arabinofuranosidase |
| contig00043.g14138 | NW_017387866.1 | FVEG_14091 | XP_018762185.1 | Carboxylic ester hydrolase (EC 3.1.1.-) |
| contig00043.g14137 | NW_017387866.1 | FVEG_14092 | XP_018762186.1 | Ricin B lectin |
| contig00030.g12800 | NW_017387870.1 | FVEG_13989 | XP_018762428.1 | SMP-30/Gluconolactonase/LRE-like region domain |
| contig00030.g12825 | NW_017387870.1 | FVEG_14015 | XP_018762460.1 | Uncharacterized protein |
| contig00030.g12833 | NW_017387870.1 | FVEG_14021 | XP_018762468.1 | Uncharacterized protein |
| contig00030.g12837 | NW_017387870.1 | FVEG_14025 | XP_018762472.1 | Uncharacterized protein |
| contig00030.g12815 | NW_017387870.1 | FVEG_17692 | XP_018762445.1 | Uncharacterized protein |
| contig00030.g12826 | NW_017387870.1 | FVEG_17699 | XP_018762461.1 | Heterokaryon incompatibility domain-containing protein |
| contig00023.g11142 | NW_017387871.1 | FVEG_14117 | XP_018762478.1 | Uncharacterized protein |
| contig00023.g11123 | NW_017387871.1 | FVEG_14136 | XP_018762500.1 | alpha-glucosidase (EC 3.2.1.20) |
| contig00023.g11119 | NW_017387871.1 | FVEG_14140 | XP_018762505.1 | Glucose-methanol-choline oxidoreductase |
| contig00018.g9698 | . | FVER14953_20274^b^ | RBQ75082.1 | hypothetical protein |
| contig00030.g12704 | . | FVER53263_20291^b^ | RBQ86236.1 | hypothetical protein |
| contig00030.g12716 | . | FVER53263_20411^b^ | RBQ87190.1 | hypothetical protein |
| contig00034.g13462 | . | FVER53590_30399^b^ | RBR11767.1 | hypothetical protein |
| contig00030.g12741 | . | FVER53263_20903^b^ | RBR01903.1 | hypothetical protein |

a Duplicated

b According to Australian isolates of Fusarium verticillioides strain BRIP14953 (QFXM00000000.1)

**Supplementary Table 2.** The secretome of *F. verticillioides* (DA42 strain) is analyzed according to Figure 2.

| **contig** | **gene name** | **score** | **COG category** | **EC** | **KEGG ko** | **BRITE** | **CAZy** | **PFAMs** |
| --- | --- | --- | --- | --- | --- | --- | --- | --- |
| contig00001.g1022 | FVEG_01038 | 1154 | BK | - | ko:K11770 | ko00000,ko03021,ko03036 | - | GATA,SNF5 |
| contig00001.g1046 | FVEG_01064 | 608 | - | - | - | - | - | - |
| contig00001.g1084 | FVEG_01111 | 514 | - | - | - | - | - | - |
| contig00001.g1086 | FVEG_01112 | 1055 | G | 4.2.2.23 | ko:K18195 | ko00000,ko01000 | PL4 | CBM-like,RhgB_N,fn3_3 |
| contig00001.g1208 | FVEG_01240 | 1234 | G | 3.2.1.14 | ko:K01183 | ko00000,ko00001,ko01000 | GH18 | Glyco_hydro_18 |
| contig00001.g1210 | FVEG_01243 | 603 | S | - | - | - | - | HAD |
| contig00001.g122 | FVEG_00120 | 276 | - | - | - | - | - | - |
| contig00001.g124 | FVEG_00121 | 115 | - | - | - | - | - | - |
| contig00001.g1243 | FVEG_01275 | 1300 | I | 3.6.1.10 | ko:K06018 | ko00000,ko01000 | - | Metallophos |
| contig00001.g127 | FVEG_00123 | 205 | - | - | - | - | - | - |
| contig00001.g1320 | FVEG_01351 | 630 | S | - | - | - | - | Lipase_GDSL_2,MU117,Spherulin4,VCBS |
| contig00001.g1330 | FVEG_01361 | 126 | S | - | - | - | - | Yos1 |
| contig00001.g1337 | FVEG_01368 | 352 | - | - | - | - | - | - |
| contig00001.g1338 | FVEG_01369 | 831 | G | 3.1.1.73 | ko:K21016 | ko00000,ko01000 | - | Lipase_3 |
| contig00001.g14 | FVEG_00014 | 423 | S | - | - | - | - | DUF3129 |
| contig00001.g141 | FVEG_00136 | 512 | E | 3.1.1.86 | ko:K15530 | ko00000,ko01000 | - | Lipase_GDSL_2 |
| contig00001.g167 | FVEG_14597 | 655 | S | - | - | - | - | Lipase_GDSL_2,VCBS |
| contig00001.g2 | FVEG_00003 | 180 | - | - | - | - | - | - |
| contig00001.g201 | FVEG_00200 | 668 | G | - | - | - | - | Glyco_hydro_16 |
| contig00001.g211 | FVEG_00212 | 1035 | O | 3.4.21.48 | ko:K01336 | ko00000,ko00001,ko01000,ko01002,ko03110 | - | Inhibitor_I9,Peptidase_S8 |
| contig00001.g224 | FVEG_00226 | 179 | - | - | - | - | - | - |
| contig00001.g247 | FVEG_00250 | 781 | L | 3.2.1.40 | ko:K05989 | ko00000,ko01000 | - | Fungal_trans |
| contig00001.g258 | FVEG_00262 | 1263 | E | 1.1.99.1 | ko:K00108 | ko00000,ko00001,ko00002,ko01000 | - | GMC_oxred_C,GMC_oxred_N |
| contig00001.g26 | FVEG_00027 | 771 | O | 3.4.21.63 | ko:K18549 | ko00000,ko01000,ko01002 | - | Inhibitor_I9,Peptidase_S8 |
| contig00001.g261 | FVEG_00265 | 264 | - | - | - | - | - | - |
| contig00001.g267 | FVEG_00271 | 983 | S | - | ko:K21403 | ko00000,ko01000 | - | His_Phos_2 |
| contig00001.g268 | FVEG_00272 | 880 | B | - | ko:K07117,ko:K11426 | ko00000,ko03036 | - | SET |
| contig00001.g269 | FVEG_00273 | 130 | - | - | - | - | - | - |
| contig00001.g287 | FVEG_00291 | 1744 | S | 1.1.3.9 | ko:K04618 | ko00000,ko00001,ko01000 | - | DUF1929,F5_F8_type_C,Kelch_1,Kelch_4,Kelch_6 |
| contig00001.g294 | FVEG_00297 | 1254 | F | - | - | - | - | Metallophos |
| contig00001.g295 | FVEG_00298 | 172 | - | - | - | - | - | - |
| contig00001.g3 | FVEG_14562 | 367 | Q | - | - | - | - | p450 |
| contig00001.g346 | FVEG_00352 | 1303 | G | - | - | - | - | Glyco_hydro_76 |
| contig00001.g365 | FVEG_00370 | 776 | O | 3.4.21.63 | ko:K18549 | ko00000,ko01000,ko01002 | - | Inhibitor_I9,Peptidase_S8 |
| contig00001.g384 | FVEG_02824 | 1004 | - | - | - | - | - | - |
| contig00001.g402 | FVEG_00392 | 1232 | - | - | - | - | - | - |
| contig00001.g407 | FVEG_00396 | 446 | S | - | - | - | - | Cutinase |
| contig00001.g408 | FVEG_00397 | 452 | S | - | - | - | - | Cutinase |
| contig00001.g46 | FVEG_00044 | 1374 | G | 3.2.1.6 | ko:K01180 | ko00000,ko01000 | - | Glyco_hydro_81 |
| contig00001.g47 | FVEG_00045 | 717 | O | 3.4.23.1,3.4.23.34 | ko:K01382,ko:K06002 | ko00000,ko00001,ko01000,ko01002 | - | Asp |
| contig00001.g491 | FVEG_14670 | 1016 | S | - | - | - | - | UPF0183 |
| contig00001.g51 | FVEG_14574 | 311 | S | - | - | - | - | - |
| contig00001.g544 | FVEG_00537 | 1245 | G | 3.2.1.113 | ko:K01230 | ko00000,ko00001,ko00002,ko01000,ko04131 | GH47 | Glyco_hydro_47 |
| contig00001.g562 | FVEG_00554 | 678 | S | - | - | - | - | - |
| contig00001.g63 | FVEG_00062 | 266 | - | - | - | - | - | - |
| contig00001.g635 | FVEG_00632 | 905 | S | - | - | - | - | DUF2401,DUF2403 |
| contig00001.g64 | FVEG_00063 | 142 | S | - | - | - | - | HET |
| contig00001.g646 | FVEG_00645 | 1043 | - | - | - | - | - | - |
| contig00001.g756 | FVEG_00761 | 677 | O | - | - | - | - | Glyco_hydro_cc |
| contig00001.g836 | FVEG_00846 | 221 | - | - | - | - | - | - |
| contig00001.g898 | FVEG_00910 | 624 | S | - | - | - | - | Terpene_synth_C |
| contig00001.g99 | FVEG_00097 | 873 | - | - | - | - | - | - |
| contig00002.g1352 | FVEG_05034 | 496 | - | - | - | - | - | - |
| contig00002.g1379 | FVEG_15586 | 1359 | O | - | - | - | - | Peptidase_S8,fn3_5 |
| contig00002.g1416 | FVEG_04968 | 1384 | S | - | - | - | - | - |
| contig00002.g1438 | FVEG_04947 | 1207 | G | 3.2.1.3 | ko:K01178 | ko00000,ko00001,ko01000 | GH15 | CBM_20,Glyco_hydro_15 |
| contig00002.g1455 | FVEG_04931 | 543 | S | - | - | - | - | DJ-1_PfpI |
| contig00002.g1501 | FVEG_04883 | 1690 | G | 3.2.1.106 | ko:K01228 | ko00000,ko00001,ko00002,ko01000,ko04147 | - | Glyco_hydro_63,Glyco_hydro_63N |
| contig00002.g1542 | FVEG_04842 | 711 | S | - | ko:K06911 | ko00000 | - | Pirin,Pirin_C |
| contig00002.g1545 | FVEG_04840 | 501 | - | - | - | - | - | - |
| contig00002.g1551 | FVEG_04834 | 1382 | S | - | - | - | - | Het-C |
| contig00002.g1618 | FVEG_04765 | 488 | - | - | - | - | - | - |
| contig00002.g1633 | FVEG_04751 | 1369 | O | 4.2.2.23 | ko:K18195 | ko00000,ko01000 | PL4 | CBM-like,fn3_3 |
| contig00002.g1641 | FVEG_04743 | 576 | O | 1.14.99.54 | ko:K19356 | ko00000,ko01000 | AA9,CBM1 | CBM_1,Glyco_hydro_61 |
| contig00002.g1665 | FVEG_04722 | 655 | - | - | - | - | - | - |
| contig00002.g1701 | FVEG_04685 | 1086 | H | 1.8.3.5,1.8.3.6 | ko:K05906 | ko00000,ko00001,ko01000 | - | NAD_binding_8,Prenylcys_lyase |
| contig00002.g1734 | FVEG_04647 | 469 | S | - | - | - | - | NPP1 |
| contig00002.g1766 | FVEG_04610 | 1082 | E | 1.1.99.18 | ko:K19069 | ko00000,ko01000 | - | GMC_oxred_C,GMC_oxred_N |
| contig00002.g1775 | FVEG_04601 | 814 | - | - | - | - | - | - |
| contig00002.g1812 | FVEG_04567 | 209 | - | - | - | - | - | - |
| contig00002.g1820 | FVEG_04559 | 1090 | G | 3.1.1.1,3.1.1.56,3.1.1.84 | ko:K03927,ko:K15743 | ko00000,ko00001,ko01000 | CE10 | COesterase |
| contig00002.g1822 | FVEG_04558 | 774 | C | 1.1.2.3 | ko:K00101 | ko00000,ko00001,ko01000 | - | FMN_dh |
| contig00002.g1826 | FVEG_04555 | 1040 | S | - | - | - | - | Beta-lactamase |
| contig00002.g1851 | FVEG_04529 | 669 | G | - | - | - | - | Lipase3_N,Lipase_3 |
| contig00002.g1879 | FVEG_04501 | 590 | S | 3.1.3.5 | ko:K03787 | ko00000,ko00001,ko01000 | - | SurE |
| contig00002.g1920 | FVEG_04460 | 472 | G | 3.2.1.151 | ko:K18576 | ko00000,ko01000 | GH12 | Glyco_hydro_12 |
| contig00002.g1923 | FVEG_04457 | 837 | O | 3.5.1.41 | ko:K01452 | ko00000,ko00001,ko01000 | - | Chitin_bind_1,Polysacc_deac_1 |
| contig00002.g1930 | FVEG_04450 | 903 | S | - | - | - | - | DUF4419 |
| contig00002.g1931 | FVEG_15481 | 220 | - | - | - | - | - | - |
| contig00002.g1957 | FVEG_04421 | 1022 | G | - | - | - | - | Glyco_hydro_43 |
| contig00002.g1978 | FVEG_04402 | 1210 | O | 3.4.16.6 | ko:K01288 | ko00000,ko01000,ko01002 | - | Peptidase_S10 |
| contig00002.g1990 | FVEG_04390 | 797 | C | 1.14.13.1 | ko:K00480 | ko00000,ko00001,ko01000 | - | FAD_binding_3,NAD_binding_8 |
| contig00002.g2014 | FVEG_04369 | 1253 | J | - | - | - | - | Amidase |
| contig00002.g2015 | FVEG_04368 | 1519 | Q | 1.4.3.21 | ko:K00276 | ko00000,ko00001,ko01000 | - | Cu_amine_oxid,Cu_amine_oxidN2,DUF1965 |
| contig00002.g2016 | FVEG_04367 | 326 | - | - | - | - | - | - |
| contig00002.g2018 | FVEG_15461 | 73.6 | O | - | - | - | - | TIL |
| contig00002.g2033 | FVEG_04351 | 733 | G | 3.2.1.14 | ko:K01183 | ko00000,ko00001,ko01000 | GH18 | Chitin_bind_1,Glyco_hydro_18 |
| contig00002.g2056 | FVEG_11018 | 53.9 | - | - | - | - | - | - |
| contig00002.g2060 | FVEG_04326 | 347 | - | - | - | - | - | - |
| contig00002.g2061 | FVEG_04325 | 1126 | S | - | - | - | - | - |
| contig00002.g2076 | FVEG_04310 | 976 | G | - | ko:K22390 | ko00000 | - | Metallophos,Metallophos_C,Pur_ac_phosph_N,Ribonuclease_3 |
| contig00003.g2112 | FVEG_05778 | 1120 | - | - | - | - | - | - |
| contig00003.g2116 | FVEG_05774 | 459 | - | - | - | - | - | - |
| contig00003.g2118 | FVEG_05772 | 516 | G | - | - | - | - | Polysacc_deac_1 |
| contig00003.g2125 | FVEG_05764 | 1261 | J | - | - | - | - | Amidase |
| contig00003.g2126 | FVEG_05763 | 741 | O | - | - | - | - | Asp |
| contig00003.g2136 | FVEG_05753 | 754 | O | - | - | - | - | Dioxygenase_C |
| contig00003.g2146 | FVEG_05745 | 209 | - | - | - | - | - | - |
| contig00003.g2152 | FVEG_05740 | 349 | - | - | - | - | - | - |
| contig00003.g2153 | FVEG_05739 | 225 | - | - | - | - | - | - |
| contig00003.g2154 | FVEG_05738 | 523 | - | - | - | - | - | - |
| contig00003.g2161 | FVEG_05730 | 518 | - | - | - | - | - | - |
| contig00003.g2162 | FVEG_05729 | 1781 | - | - | - | - | - | PAN_1 |
| contig00003.g2166 | FVEG_05726 | 1076 | Q | - | - | - | - | Lipoxygenase |
| contig00003.g2173 | FVEG_05717 | 521 | S | - | - | - | - | GLEYA |
| contig00003.g2189 | FVEG_05700 | 737 | G | 4.2.2.10 | ko:K01732 | ko00000,ko01000 | - | Pec_lyase_C |
| contig00003.g2190 | FVEG_05699 | 1246 | G | - | - | - | - | Amidohydro_3 |
| contig00003.g2193 | FVEG_05697 | 935 | G | 3.2.1.171 | ko:K18580 | ko00000,ko01000 | GH28 | Glyco_hydro_28 |
| contig00003.g2196 | FVEG_05694 | 994 | - | - | - | - | - | - |
| contig00003.g2200 | FVEG_05691 | 680 | I | - | - | - | - | Abhydrolase_1 |
| contig00003.g2203 | FVEG_05689 | 1372 | O | 3.2.1.55 | ko:K01209 | ko00000,ko00001,ko01000 | GH51 | Alpha-L-AF_C,CBM_4_9 |
| contig00003.g2214 | FVEG_05679 | 489 | S | - | ko:K20628 | ko00000 | - | Cerato-platanin,Pollen_allerg_1 |
| contig00003.g2238 | FVEG_05658 | 1228 | O | - | - | - | - | Peptidase_S10 |
| contig00003.g2242 | FVEG_05654 | 745 | S | - | - | - | - | Abhydrolase_6,Hydrolase_4 |
| contig00003.g2246 | FVEG_05650 | 931 | O | - | - | - | - | Asp |
| contig00003.g2253 | FVEG_05642 | 672 | S | - | - | - | - | LPMO_10 |
| contig00003.g2292 | FVEG_05601 | 988 | O | - | - | - | - | PA,Peptidase_M28 |
| contig00003.g2300 | FVEG_05596 | 889 | O | - | - | - | - | Chitin_bind_1,Polysacc_deac_1 |
| contig00003.g2331 | FVEG_05561 | 1766 | O | - | - | - | - | FlgD_ig,PA,Peptidase_S8,fn3_5 |
| contig00003.g2350 | FVEG_05540 | 1023 | G | - | - | - | - | COesterase |
| contig00003.g2368 | FVEG_05521 | 1786 | G | 3.2.1.21 | ko:K05349 | ko00000,ko00001,ko01000 | GH3 | Fn3-like,Glyco_hydro_3,Glyco_hydro_3_C |
| contig00003.g2378 | FVEG_05513 | 1281 | P | 3.1.3.1 | ko:K01113 | ko00000,ko00001,ko00002,ko01000 | - | PhoD,PhoD_N |
| contig00003.g2382 | FVEG_05510 | 867 | O | - | - | - | - | M20_dimer,Peptidase_M20 |
| contig00003.g2384 | FVEG_05507 | 1542 | G | 3.2.1.58 | ko:K01210 | ko00000,ko00001,ko01000 | - | Pectate_lyase_3 |
| contig00003.g2388 | FVEG_05503 | 617 | D | - | - | - | - | WD40 |
| contig00003.g2400 | FVEG_05497 | 310 | O | - | ko:K03671 | ko00000,ko00001,ko03110 | - | Thioredoxin |
| contig00003.g2464 | FVEG_05433 | 285 | - | - | - | - | - | - |
| contig00003.g2496 | FVEG_05394 | 224 | - | - | - | - | - | - |
| contig00003.g2539 | FVEG_05347 | 1200 | GO | - | - | - | - | WSC |
| contig00003.g2666 | FVEG_09387 | 329 | S | - | - | - | - | - |
| contig00004.g2864 | FVEG_15153 | 314 | - | - | - | - | - | - |
| contig00004.g2910 | FVEG_02982 | 374 | S | - | - | - | - | - |
| contig00004.g2931 | FVEG_03005 | 281 | S | - | - | - | - | Cerato-platanin |
| contig00004.g2971 | FVEG_03046 | 483 | S | - | - | - | - | DUF3455 |
| contig00004.g2999 | FVEG_03072 | 1264 | O | 3.4.14.9 | ko:K01279 | ko00000,ko00001,ko01000,ko01002,ko03110,ko04147 | - | Peptidase_S8,Pro-kuma_activ |
| contig00004.g3017 | FVEG_03090 | 1228 | - | - | - | - | - | - |
| contig00004.g3048 | FVEG_03121 | 829 | O | - | ko:K14002 | ko00000,ko00001 | - | DnaJ,DnaJ_C,DnaJ_CXXCXGXG |
| contig00004.g3052 | FVEG_03124 | 1208 | Q | - | - | - | - | Cu-oxidase,Cu-oxidase_2,Cu-oxidase_3 |
| contig00004.g3068 | FVEG_03140 | 1005 | G | 3.2.1.8 | ko:K01181 | ko00000,ko01000 | - | CBM_1,Glyco_hydro_10 |
| contig00004.g3071 | FVEG_03143 | 298 | - | - | - | - | - | - |
| contig00004.g3072 | FVEG_03144 | 666 | C | 3.6.1.1 | ko:K01507 | ko00000,ko00001,ko01000 | - | Pyrophosphatase |
| contig00004.g3143 | FVEG_03210 | 399 | - | - | - | - | - | - |
| contig00004.g3175 | FVEG_03245 | 744 | O | - | - | - | - | Inhibitor_I9,Peptidase_S8 |
| contig00004.g3189 | FVEG_03260 | 780 | S | - | - | - | - | CBM_1 |
| contig00004.g3204 | FVEG_03274 | 1006 | S | - | - | - | - | MU117 |
| contig00004.g3205 | FVEG_03275 | 697 | D | - | - | - | - | Pkinase |
| contig00004.g3207 | FVEG_03277 | 704 | E | 3.5.1.1 | ko:K01424 | ko00000,ko00001,ko01000 | - | Asparaginase |
| contig00004.g3213 | FVEG_15231 | 773 | O | 5.3.4.1 | ko:K09580 | ko00000,ko00001,ko01000,ko03110,ko04131,ko04147 | - | Thioredoxin,Thioredoxin_6 |
| contig00004.g3214 | FVEG_03284 | 613 | - | - | - | - | - | - |
| contig00004.g3223 | FVEG_03292 | 421 | - | - | - | - | - | - |
| contig00004.g3239 | FVEG_03305 | 45.1 | - | - | - | - | - | - |
| contig00004.g3242 | FVEG_03307 | 332 | - | - | - | - | - | - |
| contig00004.g3265 | FVEG_03324 | 466 | G | 3.2.1.139 | ko:K01235 | ko00000,ko01000 | - | Glyco_hydro_67C,Glyco_hydro_67M,Glyco_hydro_67N |
| contig00004.g3276 | FVEG_03334 | 1033 | - | - | - | - | - | - |
| contig00004.g3290 | FVEG_03346 | 261 | - | - | - | - | - | - |
| contig00004.g3297 | FVEG_03351 | 358 | G | - | - | - | - | Cutinase |
| contig00004.g3308 | FVEG_03362 | 974 | G | 3.2.1.171 | ko:K18580 | ko00000,ko01000 | GH28 | CBM_1,Glyco_hydro_28 |
| contig00004.g3339 | FVEG_03395 | 444 | G | 3.1.1.74 | ko:K08095 | ko00000,ko01000 | - | Cutinase |
| contig00004.g3342 | FVEG_03398 | 868 | I | - | - | - | - | LIP |
| contig00004.g3360 | FVEG_03416 | 761 | S | - | - | - | - | Alginate_lyase |
| contig00004.g3383 | FVEG_09332 | 692 | - | - | - | - | - | - |
| contig00004.g3397 | FVEG_09348 | 264 | S | - | - | - | - | PAN_1,PAN_4 |
| contig00004.g3399 | FVEG_09350 | 504 | - | - | - | - | - | PAN_4 |
| contig00004.g3410 | FVEG_09361 | 646 | Q | - | - | - | - | Esterase_phd |
| contig00004.g3419 | FVEG_09370 | 904 | G | - | - | - | - | Glyco_hydro_64 |
| contig00004.g3427 | FVEG_09375 | 520 | O | - | - | - | - | Trypsin |
| contig00004.g3455 | FVEG_09405 | 575 | S | - | - | - | - | CBM_1,Lipase_GDSL,Lipase_GDSL_2 |
| contig00004.g3456 | FVEG_09406 | 315 | S | - | - | - | - | - |
| contig00004.g3460 | FVEG_09409 | 94 | - | - | - | - | - | - |
| contig00004.g3468 | FVEG_16579 | 786 | - | - | - | - | - | - |
| contig00004.g3469 | FVEG_09416 | 1152 | S | - | - | - | - | MU117 |
| contig00004.g3482 | FVEG_16584 | 153 | - | - | - | - | - | - |
| contig00004.g3492 | FVEG_09438 | 230 | - | - | - | - | - | - |
| contig00004.g3495 | FVEG_09440 | 689 | S | - | - | - | - | - |
| contig00004.g3498 | FVEG_09443 | 1293 | O | - | - | - | - | Pectate_lyase_3 |
| contig00004.g3510 | FVEG_12952 | 706 | G | 3.2.1.55 | ko:K20844 | ko00000,ko00001,ko01000 | CBM42,GH54 | AbfB,ArabFuran-catal |
| contig00005.g3516 | FVEG_03871 | 246 | A | - | - | - | - | DEAD,Helicase_C,OrsD |
| contig00005.g3523 | FVEG_09955 | 106 | S | - | - | - | - | CAP |
| contig00005.g3526 | FVEG_16697 | 656 | K | - | - | - | - | Fungal_trans,Zn_clus |
| contig00005.g3532 | FVEG_09947 | 919 | G | 3.1.1.73 | ko:K09252 | ko00000,ko01000 | - | Tannase |
| contig00005.g3535 | FVEG_09943 | 389 | - | - | - | - | - | - |
| contig00005.g3543 | FVEG_09935 | 105 | - | - | - | - | - | - |
| contig00005.g3562 | FVEG_09915 | 70.1 | - | - | - | - | - | PAN_1,PAN_4 |
| contig00005.g3564 | FVEG_09913 | 955 | - | - | - | - | - | - |
| contig00005.g3612 | FVEG_09861 | 1536 | G | 3.2.1.37 | ko:K15920 | ko00000,ko00001,ko01000 | GH3 | Fn3-like,Glyco_hydro_3,Glyco_hydro_3_C |
| contig00005.g3619 | FVEG_09857 | 3060 | S | - | - | - | - | Lipase_GDSL_2,fn3 |
| contig00005.g3634 | FVEG_09844 | 106 | - | - | - | - | - | - |
| contig00005.g3635 | FVEG_09843 | 286 | - | - | - | - | - | - |
| contig00005.g3640 | FVEG_16679 | 641 | S | - | - | - | - | - |
| contig00005.g3652 | FVEG_09828 | 254 | S | - | - | - | - | CVNH |
| contig00005.g3657 | FVEG_09826 | 665 | S | - | - | - | - | DUF1996 |
| contig00005.g3659 | FVEG_09824 | 745 | E | 1.4.3.1,1.4.3.3 | ko:K00272,ko:K00273 | ko00000,ko00001,ko01000 | - | DAO |
| contig00005.g3662 | FVEG_09821 | 372 | O | 5.3.4.1 | ko:K09580 | ko00000,ko00001,ko01000,ko03110,ko04131,ko04147 | - | Thioredoxin,Thioredoxin_6 |
| contig00005.g3700 | FVEG_08301 | 634 | O | - | - | - | - | Glyco_hydro_16 |
| contig00005.g3707 | FVEG_08305 | 590 | E | 1.14.11.2 | ko:K00472 | ko00000,ko00001,ko01000 | - | 2OG-FeII_Oxy_3 |
| contig00005.g3709 | FVEG_08309 | 521 | S | - | - | - | - | MFS_1 |
| contig00005.g3722 | FVEG_00094 | 131 | S | - | - | - | - | Candida_ALS |
| contig00005.g3738 | FVEG_08331 | 430 | S | - | - | - | - | DUF3129 |
| contig00005.g3754 | FVEG_08344 | 922 | G | - | - | - | - | CBM_1,CBM_6,Glyco_hydro_43 |
| contig00005.g3755 | FVEG_08345 | 1052 | G | - | - | - | - | Glyco_hydro_43 |
| contig00005.g3757 | FVEG_08347 | 168 | - | - | - | - | - | - |
| contig00005.g3758 | FVEG_08348 | 302 | - | - | - | - | - | - |
| contig00005.g3759 | FVEG_08349 | 58.2 | - | - | - | - | - | - |
| contig00005.g3766 | FVEG_08355 | 1600 | S | - | - | - | - | Bac_rhamnosid6H,Bac_rhamnosid_C |
| contig00005.g3779 | FVEG_16301 | 271 | C | - | - | - | - | ADH_N,ADH_zinc_N |
| contig00005.g3786 | FVEG_08373 | 498 | S | - | - | - | - | Peptidase_A4 |
| contig00005.g3803 | FVEG_08386 | 277 | - | - | - | - | - | - |
| contig00005.g3810 | FVEG_08392 | 301 | - | - | - | - | - | - |
| contig00005.g3815 | FVEG_08397 | 456 | - | - | - | - | - | - |
| contig00005.g3821 | FVEG_08403 | 809 | O | - | - | - | - | Asp |
| contig00005.g3832 | FVEG_08413 | 966 | S | - | - | - | - | Glyco_hydro_39 |
| contig00005.g3840 | FVEG_08421 | 660 | G | - | - | - | - | Glyco_hydro_43 |
| contig00005.g3841 | FVEG_08422 | 1308 | C | - | - | - | - | BBE,FAD_binding_4 |
| contig00005.g3848 | FVEG_08430 | 986 | Q | - | - | - | - | p450 |
| contig00005.g3853 | FVEG_08435 | 947 | I | - | - | - | - | - |
| contig00005.g3872 | FVEG_08451 | 743 | G | 3.2.1.15,3.2.1.67 | ko:K01184,ko:K01213 | ko00000,ko00001,ko00002,ko01000 | - | Glyco_hydro_28 |
| contig00005.g3879 | FVEG_08459 | 248 | - | - | - | - | - | - |
| contig00005.g3881 | FVEG_08461 | 1157 | G | - | - | - | - | COesterase |
| contig00005.g3890 | FVEG_08470 | 878 | G | 3.2.1.14 | ko:K01183 | ko00000,ko00001,ko01000 | GH18 | Glyco_hydro_18 |
| contig00005.g3933 | FVEG_08511 | 1315 | C | 1.1.5.3 | ko:K00111 | ko00000,ko00001,ko01000 | - | DAO,DAO_C,EF-hand_7 |
| contig00005.g3939 | FVEG_08518 | 1120 | G | - | - | - | - | COesterase |
| contig00005.g3950 | FVEG_16349 | 1779 | - | - | - | - | - | - |
| contig00005.g3967 | FVEG_08546 | 506 | S | - | - | - | - | Fungal_trans_2,Zn_clus |
| contig00005.g3977 | FVEG_08555 | 1373 | O | 1.1.3.9 | ko:K04618 | ko00000,ko00001,ko01000 | - | DUF1929,F5_F8_type_C,Kelch_1,Kelch_4,Kelch_6 |
| contig00005.g3980 | FVEG_08558 | 443 | - | - | - | - | - | - |
| contig00005.g4007 | FVEG_08586 | 823 | - | - | - | - | - | - |
| contig00005.g4020 | FVEG_08596 | 495 | S | - | - | - | - | TNT |
| contig00005.g4030 | FVEG_08605 | 1206 | G | - | - | - | - | Glyco_hydro_43 |
| contig00005.g4033 | FVEG_16373 | 1485 | S | - | - | - | - | WSC |
| contig00005.g4050 | FVEG_16379 | 488 | - | - | - | - | - | - |
| contig00005.g4066 | FVEG_08638 | 291 | - | - | - | - | - | - |
| contig00005.g4079 | FVEG_08650 | 1259 | G | - | - | - | - | Fn3-like,Glyco_hydro_3,Glyco_hydro_3_C |
| contig00005.g4087 | FVEG_08658 | 753 | S | - | ko:K21830 | ko00000 | - | - |
| contig00005.g4088 | FVEG_08659 | 2079 | G | 3.2.1.23 | ko:K01190 | ko00000,ko00001,ko01000 | - | BetaGal_dom2,BetaGal_dom3,BetaGal_dom4_5,Glyco_hydro_35 |
| contig00005.g4091 | FVEG_16393 | 276 | - | - | - | - | - | - |
| contig00005.g4098 | FVEG_16396 | 1322 | G | - | - | - | - | Glyco_hydro_2,Glyco_hydro_2_C,Glyco_hydro_2_N |
| contig00005.g4110 | FVEG_08679 | 783 | O | 3.4.21.63 | ko:K18549 | ko00000,ko01000,ko01002 | - | Inhibitor_I9,Peptidase_S8 |
| contig00005.g4111 | FVEG_16398 | 282 | S | - | - | - | - | - |
| contig00005.g4117 | FVEG_08686 | 397 | S | - | ko:K13449 | ko00000,ko00001 | - | CAP |
| contig00005.g4119 | FVEG_08688 | 182 | - | - | - | - | - | - |
| contig00005.g4121 | FVEG_08690 | 1025 | C | - | - | - | - | BBE,FAD_binding_4 |
| contig00005.g4124 | FVEG_08694 | 1328 | C | - | - | - | - | BBE,FAD_binding_4 |
| contig00005.g4132 | FVEG_08702 | 1527 | E | 1.1.99.18 | ko:K19069 | ko00000,ko01000 | - | CBM_1,CDH-cyt,GMC_oxred_C,GMC_oxred_N |
| contig00005.g4137 | FVEG_08707 | 1171 | E | - | - | - | - | GMC_oxred_C,GMC_oxred_N |
| contig00005.g4152 | FVEG_08723 | 693 | S | - | - | - | - | Glyco_hydro_16 |
| contig00005.g4162 | FVEG_16409 | 1118 | C | - | - | - | - | BBE,FAD_binding_4,NTP_transf_9 |
| contig00005.g4167 | FVEG_08734 | 506 | E | - | - | - | - | Pectate_lyase |
| contig00005.g4178 | FVEG_08746 | 255 | - | - | - | - | - | - |
| contig00005.g4180 | FVEG_08748 | 1030 | - | - | - | - | - | - |
| contig00005.g4191 | FVEG_08757 | 919 | S | - | - | - | - | Peroxidase_2 |
| contig00005.g4193 | FVEG_08758 | 353 | - | - | - | - | - | - |
| contig00005.g4217 | FVEG_08780 | 843 | O | - | - | - | - | Peptidase_M14 |
| contig00005.g4220 | FVEG_08782 | 460 | S | - | - | - | - | TNT |
| contig00005.g4221 | FVEG_08783 | 501 | G | 1.14.99.54 | ko:K19356 | ko00000,ko01000 | AA9,CBM1 | Glyco_hydro_61 |
| contig00005.g4223 | FVEG_08785 | 1150 | G | - | - | - | - | COesterase,TB2_DP1_HVA22 |
| contig00005.g4238 | FVEG_16443 | 679 | - | - | - | - | - | - |
| contig00005.g4240 | FVEG_08802 | 394 | - | - | - | - | - | - |
| contig00005.g4246 | FVEG_08808 | 390 | - | - | - | - | - | - |
| contig00006.g4313 | FVEG_02506 | 600 | - | - | - | - | - | - |
| contig00006.g4411 | FVEG_02407 | 474 | - | - | - | - | - | - |
| contig00006.g4413 | FVEG_02406 | 907 | S | - | - | - | - | Abhydrolase_6,LIP |
| contig00006.g4415 | FVEG_02404 | 523 | Q | - | - | - | - | DLH |
| contig00006.g4416 | FVEG_02403 | 1007 | C | - | - | - | - | BBE,FAD_binding_4 |
| contig00006.g4514 | FVEG_02309 | 362 | - | - | - | - | - | - |
| contig00006.g4633 | FVEG_14997 | 1497 | - | - | - | - | - | - |
| contig00006.g4642 | FVEG_02167 | 627 | S | - | - | - | - | S1-P1_nuclease |
| contig00006.g4643 | FVEG_02166 | 488 | O | - | - | - | - | - |
| contig00006.g4676 | FVEG_02133 | 808 | O | 3.4.21.63 | ko:K18549 | ko00000,ko01000,ko01002 | - | Inhibitor_I9,Peptidase_S8 |
| contig00006.g4712 | FVEG_02095 | 958 | E | 1.5.3.1,1.5.3.7 | ko:K00306 | ko00000,ko00001,ko01000 | - | DAO |
| contig00007.g4778 | FVEG_15302 | 654 | Q | 1.1.1.300 | ko:K11153 | ko00000,ko00001,ko01000 | - | adh_short |
| contig00007.g4780 | FVEG_15303 | 332 | - | - | - | - | - | - |
| contig00007.g4793 | FVEG_03639 | 514 | S | - | - | - | - | LicD |
| contig00007.g4796 | FVEG_03642 | 1211 | G | - | - | - | - | Glyco_hydro_3,Glyco_hydro_3_C |
| contig00007.g4802 | FVEG_03648 | 1056 | E | - | - | - | - | Peptidase_C69 |
| contig00007.g4806 | FVEG_03654 | 226 | S | - | - | - | - | - |
| contig00007.g4809 | FVEG_03657 | 1105 | E | 2.3.2.2,3.4.19.13 | ko:K00681 | ko00000,ko00001,ko01000,ko01002 | - | G_glu_transpept |
| contig00007.g4816 | FVEG_03664 | 700 | S | 4.4.1.28 | ko:K20285,ko:K22207 | ko00000,ko00001,ko01000,ko04131 | - | BTB,Kelch_1,Kelch_3,Kelch_4,Kelch_5 |
| contig00007.g4820 | FVEG_03669 | 352 | - | - | - | - | - | - |
| contig00007.g4826 | FVEG_03675 | 1142 | G | - | - | - | - | COesterase,TB2_DP1_HVA22 |
| contig00007.g4835 | FVEG_03684 | 520 | O | - | - | - | - | Peptidase_M43 |
| contig00007.g4847 | FVEG_03694 | 1234 | E | - | - | - | - | Amino_oxidase |
| contig00007.g4861 | FVEG_03708 | 277 | S | - | - | - | - | MU117 |
| contig00007.g4879 | FVEG_03727 | 590 | I | - | - | - | - | Lipase_GDSL,Lipase_GDSL_2 |
| contig00007.g4890 | FVEG_03738 | 559 | S | - | - | - | - | DUF3129 |
| contig00007.g4906 | FVEG_03751 | 540 | - | - | - | - | - | - |
| contig00007.g4977 | FVEG_15341 | 234 | - | - | - | - | - | - |
| contig00007.g5053 | FVEG_15359 | 117 | KL | - | - | - | - | Helicase_C,SNF2_N |
| contig00007.g5062 | FVEG_03905 | 810 | G | - | - | - | - | - |
| contig00007.g5063 | FVEG_03906 | 509 | G | 3.2.1.181 | ko:K20832 | ko00000,ko01000 | GH16 | CBM_1,Glyco_hydro_16 |
| contig00007.g5085 | FVEG_03930 | 823 | O | - | - | - | - | Inhibitor_I9,Peptidase_S8 |
| contig00007.g5113 | FVEG_03960 | 798 | O | 2.3.2.5 | ko:K00683 | ko00000,ko01000 | - | Peptidase_M28 |
| contig00007.g5124 | FVEG_03971 | 565 | - | - | - | - | - | - |
| contig00007.g5142 | FVEG_03990 | 4977 | S | - | - | - | - | - |
| contig00007.g5149 | FVEG_03997 | 1358 | O | 3.2.1.58 | ko:K01210 | ko00000,ko00001,ko01000 | - | Pectate_lyase_3 |
| contig00007.g5172 | FVEG_04019 | 1273 | O | - | ko:K09490 | ko00000,ko00001,ko03110,ko04131,ko04147 | - | HSP70 |
| contig00007.g5175 | FVEG_04022 | 1179 | O | - | - | - | - | Exo_endo_phos |
| contig00007.g5222 | FVEG_04071 | 1161 | S | 1.14.18.1 | ko:K00505 | ko00000,ko00001,ko00002,ko01000 | - | Tyrosinase |
| contig00007.g5249 | FVEG_04097 | 625 | S | - | - | - | - | CAP |
| contig00007.g5273 | FVEG_04124 | 348 | S | - | - | - | - | - |
| contig00007.g5289 | FVEG_04141 | 345 | S | - | - | - | - | E1_DerP2_DerF2 |
| contig00008.g5322 | FVEG_09321 | 410 | - | - | - | - | - | - |
| contig00008.g5356 | FVEG_09287 | 155 | S | - | ko:K18188 | ko00000,ko03029 | - | PET117 |
| contig00008.g5358 | FVEG_09285 | 672 | O | - | - | - | - | CBM_1,Glyco_hydro_45 |
| contig00008.g5359 | FVEG_09284 | 597 | O | - | - | - | - | Glyco_hydro_16 |
| contig00008.g5375 | FVEG_16549 | 1593 | - | - | - | - | - | - |
| contig00008.g5408 | FVEG_09233 | 586 | S | - | - | - | - | Abhydrolase_1 |
| contig00008.g5488 | FVEG_09149 | 927 | Q | 1.11.1.13 | ko:K20205 | ko00000,ko01000 | - | peroxidase |
| contig00008.g5496 | FVEG_09142 | 262 | - | - | - | - | - | - |
| contig00008.g5519 | FVEG_09116 | 527 | G | - | - | - | - | Glyco_hydro_61 |
| contig00008.g5556 | FVEG_09073 | 1182 | O | 3.4.14.9 | ko:K01279 | ko00000,ko00001,ko01000,ko01002,ko03110,ko04147 | - | Peptidase_S8,Pro-kuma_activ |
| contig00008.g5577 | FVEG_16512 | 813 | - | - | - | - | - | - |
| contig00008.g5596 | FVEG_09036 | 300 | - | - | - | - | - | - |
| contig00008.g5597 | FVEG_09035 | 588 | A | 3.1.27.1 | ko:K01166 | ko00000,ko01000,ko03016 | - | Ribonuclease_T2 |
| contig00008.g5598 | FVEG_09034 | 1082 | G | - | - | - | - | Glyco_hydro_43 |
| contig00008.g5624 | FVEG_09010 | 444 | S | - | - | - | - | Cupin_1 |
| contig00008.g5625 | FVEG_09009 | 966 | S | - | - | - | - | Abhydrolase_6,Hydrolase_4,LIP |
| contig00008.g5656 | FVEG_08978 | 779 | G | - | - | - | - | Lactonase |
| contig00008.g5736 | FVEG_08899 | 1018 | S | - | - | - | - | Ish1 |
| contig00008.g5739 | FVEG_08896 | 240 | - | - | - | - | - | - |
| contig00008.g5740 | FVEG_08895 | 380 | - | - | - | - | - | - |
| contig00008.g5743 | FVEG_08891 | 1398 | O | 1.1.3.9 | ko:K04618 | ko00000,ko00001,ko01000 | - | DUF1929,F5_F8_type_C,Kelch_1,Kelch_4,Kelch_6 |
| contig00008.g5763 | FVEG_08872 | 449 | - | - | - | - | - | - |
| contig00008.g5764 | FVEG_08871 | 149 | S | - | - | - | - | CFEM |
| contig00008.g5766 | FVEG_08870 | 1056 | G | 3.1.1.8 | ko:K01050 | ko00000,ko01000 | - | COesterase |
| contig00008.g5770 | FVEG_08867 | 1730 | G | - | - | - | - | Glyco_hydro_71 |
| contig00008.g5780 | FVEG_08858 | 890 | S | - | - | - | - | Alginate_lyase |
| contig00008.g5785 | FVEG_08854 | 793 | O | - | - | - | - | - |
| contig00008.g5791 | FVEG_16455 | 814 | S | - | - | - | - | MU117 |
| contig00008.g5799 | FVEG_08840 | 68.6 | P | - | - | - | - | Sugar_tr |
| contig00008.g5804 | FVEG_08836 | 1108 | G | - | - | - | - | Glyco_hydro_43 |
| contig00008.g5807 | FVEG_08833 | 555 | O | - | - | - | - | CBM_1,Lipase_GDSL,Lipase_GDSL_2 |
| contig00008.g5812 | FVEG_08828 | 468 | G | 3.2.1.8 | ko:K01181 | ko00000,ko01000 | - | CBM_1,Glyco_hydro_11 |
| contig00008.g5816 | FVEG_08825 | 890 | G | 3.2.1.91 | ko:K19668 | ko00000,ko00001,ko01000 | GH6 | CBM_1,Glyco_hydro_6 |
| contig00008.g5817 | FVEG_08824 | 1718 | G | 3.2.1.139 | ko:K01235 | ko00000,ko01000 | - | Glyco_hydro_67C,Glyco_hydro_67M,Glyco_hydro_67N |
| contig00008.g5820 | FVEG_08821 | 547 | O | - | - | - | - | CBM_1,Glyco_hydro_61 |
| contig00009.g5850 | FVEG_09988 | 709 | - | - | - | - | - | - |
| contig00009.g5860 | FVEG_09998 | 73.2 | - | - | - | - | - | - |
| contig00009.g5862 | FVEG_10000 | 102 | - | - | - | - | - | - |
| contig00009.g5901 | FVEG_10031 | 292 | - | - | - | - | - | - |
| contig00009.g5902 | FVEG_10032 | 1115 | G | 3.2.1.113 | ko:K01230 | ko00000,ko00001,ko00002,ko01000,ko04131 | GH47 | Glyco_hydro_47 |
| contig00009.g5926 | FVEG_10058 | 305 | - | - | - | - | - | - |
| contig00009.g5928 | FVEG_10059 | 66.6 | - | - | - | - | - | - |
| contig00009.g5937 | FVEG_10071 | 355 | S | - | - | - | - | Phospholip_A2_3 |
| contig00009.g5938 | FVEG_10072 | 444 | S | - | - | - | - | PBP |
| contig00009.g5942 | FVEG_10077 | 937 | S | - | - | - | - | Ferritin_2 |
| contig00009.g5948 | FVEG_10083 | 1424 | G | 3.2.1.7,3.2.1.80 | ko:K03332,ko:K22245 | ko00000,ko00001,ko01000 | - | Glyco_hydro_32C,Glyco_hydro_32N |
| contig00009.g5958 | FVEG_10092 | 660 | H | - | - | - | - | Abhydrolase_1 |
| contig00009.g5962 | FVEG_16722 | 1974 | G | - | - | - | - | BetaGal_dom2,BetaGal_dom3,BetaGal_dom4_5,Glyco_hydro_35 |
| contig00009.g5963 | FVEG_10098 | 837 | O | - | - | - | - | Glyco_hydro_32N,Glyco_hydro_43,Laminin_G_3 |
| contig00009.g5965 | FVEG_10100 | 416 | - | - | - | - | - | - |
| contig00009.g5966 | FVEG_10101 | 626 | G | 4.2.2.2 | ko:K01728 | ko00000,ko00001,ko01000 | - | Pec_lyase_C |
| contig00009.g5967 | FVEG_10102 | 550 | E | - | - | - | - | Pectate_lyase |
| contig00009.g5968 | FVEG_10103 | 489 | Q | - | - | - | - | Isochorismatase |
| contig00009.g5976 | FVEG_10111 | 1177 | C | - | - | - | - | BBE,FAD_binding_4 |
| contig00009.g5980 | FVEG_10115 | 1160 | E | 3.4.17.4 | ko:K01293 | ko00000,ko01000,ko01002 | - | M20_dimer,Peptidase_M20,Peptidase_M28 |
| contig00009.g5981 | FVEG_10116 | 651 | O | - | - | - | - | CBM_1,Glyco_hydro_61 |
| contig00009.g5982 | FVEG_10117 | 259 | T | - | - | - | - | - |
| contig00009.g5985 | FVEG_10119 | 577 | S | - | - | - | - | HRXXH |
| contig00009.g6013 | FVEG_10146 | 1025 | G | - | - | - | - | Exo_endo_phos |
| contig00009.g6017 | FVEG_10149 | 914 | C | - | - | - | - | BBE,FAD_binding_4 |
| contig00009.g6064 | FVEG_10189 | 1168 | J | 3.5.1.4 | ko:K01426 | ko00000,ko00001,ko01000 | - | Amidase |
| contig00009.g6072 | FVEG_16752 | 613 | - | - | - | - | - | - |
| contig00009.g6111 | FVEG_10241 | 452 | G | - | ko:K20628 | ko00000 | - | DPBB_1,Pollen_allerg_1 |
| contig00009.g6154 | FVEG_10284 | 981 | O | 3.4.16.6 | ko:K01288 | ko00000,ko01000,ko01002 | - | Peptidase_S10 |
| contig00009.g6203 | FVEG_10333 | 889 | O | - | - | - | - | Chitin_bind_1,Polysacc_deac_1 |
| contig00009.g6234 | FVEG_10364 | 565 | S | - | - | - | - | - |
| contig00009.g6278 | FVEG_10404 | 376 | S | - | - | - | - | AltA1 |
| contig00009.g6306 | FVEG_10428 | 491 | - | - | - | - | - | - |
| contig00009.g6323 | FVEG_10446 | 1108 | S | - | ko:K09704 | ko00000 | - | Glyco_hydro_125 |
| contig00010.g6439 | FVEG_07082 | 781 | G | 3.5.3.11 | ko:K01480 | ko00000,ko00001,ko00002,ko01000 | - | Arginase |
| contig00010.g6480 | FVEG_07124 | 471 | - | - | - | - | - | - |
| contig00010.g6487 | FVEG_16023 | 507 | S | - | - | - | - | Glyco_hydro_64 |
| contig00010.g6542 | FVEG_02601 | 518 | - | - | - | - | - | - |
| contig00010.g6566 | FVEG_02627 | 961 | S | - | - | - | - | CDT1_C |
| contig00010.g6627 | FVEG_02691 | 1140 | G | - | - | - | - | Amidohydro_3 |
| contig00010.g6631 | FVEG_02695 | 448 | S | - | ko:K17787 | ko00000,ko03029 | - | AIM5 |
| contig00011.g6783 | FVEG_06522 | 1431 | S | 1.1.3.9 | ko:K04618 | ko00000,ko00001,ko01000 | - | DUF1929,Kelch_1,Kelch_6,PAN_1 |
| contig00011.g6787 | FVEG_06525 | 713 | O | - | - | - | - | Dioxygenase_C |
| contig00011.g6800 | FVEG_06538 | 178 | S | - | - | - | - | - |
| contig00011.g6802 | FVEG_15883 | 196 | I | - | - | - | - | Glyco_hydro_12,Glycos_transf_2 |
| contig00011.g6809 | FVEG_15886 | 630 | - | - | - | - | - | - |
| contig00011.g6842 | FVEG_06580 | 1298 | J | - | - | - | - | Amidase |
| contig00011.g6846 | FVEG_06584 | 997 | C | - | - | - | - | FAD_binding_4 |
| contig00011.g6888 | FVEG_15904 | 908 | G | - | - | - | - | COesterase |
| contig00011.g6891 | FVEG_06631 | 310 | S | - | - | - | - | - |
| contig00011.g6913 | FVEG_15912 | 252 | O | - | - | - | - | - |
| contig00011.g6966 | FVEG_06704 | 946 | OP | - | ko:K01302 | ko00000,ko01000,ko01002 | - | PA,Peptidase_M28 |
| contig00011.g6977 | FVEG_06715 | 219 | - | - | - | - | - | - |
| contig00011.g6989 | FVEG_06728 | 1649 | G | 3.2.1.21 | ko:K05349 | ko00000,ko00001,ko01000 | GH3 | Fn3-like,Glyco_hydro_3,Glyco_hydro_3_C |
| contig00011.g7002 | FVEG_06739 | 573 | S | - | - | - | - | Ferritin_2 |
| contig00011.g7012 | FVEG_06750 | 676 | IO | 3.1.2.22 | ko:K01074 | ko00000,ko00001,ko01000,ko01004 | - | Palm_thioest |
| contig00011.g7037 | FVEG_06777 | 856 | G | - | - | - | - | LysM |
| contig00011.g7039 | FVEG_06779 | 3020 | G | - | - | - | - | Glyco_hydro_18,Hce2,LysM |
| contig00011.g7065 | FVEG_06805 | 386 | - | - | - | - | - | - |
| contig00011.g7067 | FVEG_06807 | 497 | S | - | - | - | - | Lipase_GDSL_2,MU117,Spherulin4,VCBS |
| contig00011.g7085 | FVEG_06822 | 552 | O | 3.5.1.104 | ko:K22278 | ko00000,ko01000 | - | Chitin_bind_1,Polysacc_deac_1 |
| contig00011.g7096 | FVEG_06833 | 1018 | E | - | - | - | - | GMC_oxred_C,GMC_oxred_N |
| contig00011.g7120 | FVEG_06859 | 1180 | Q | 1.14.18.1 | ko:K00505 | ko00000,ko00001,ko00002,ko01000 | - | Tyrosinase |
| contig00011.g7123 | FVEG_06863 | 787 | G | 2.4.1.144 | ko:K00737 | ko00000,ko00001,ko00002,ko01000,ko01003 | GT17 | Glyco_transf_17 |
| contig00011.g7125 | FVEG_06865 | 1398 | G | 3.2.1.28 | ko:K01194 | ko00000,ko00001,ko00537,ko01000 | GH37 | Trehalase |
| contig00011.g7140 | FVEG_15951 | 84 | - | - | - | - | - | - |
| contig00011.g7160 | FVEG_06900 | 800 | G | 3.2.1.75 | ko:K22277 | ko00000,ko01000 | GH5 | Cellulase |
| contig00012.g7266 | FVEG_06212 | 75.1 | - | - | - | - | - | - |
| contig00012.g7282 | FVEG_06194 | 273 | - | - | - | - | - | - |
| contig00012.g7302 | FVEG_06174 | 1001 | O | - | - | - | - | Glyco_hydro_61 |
| contig00012.g7400 | FVEG_06068 | 471 | S | - | - | - | - | Cupin_1 |
| contig00012.g7425 | FVEG_06039 | 1466 | T | 3.1.3.8 | ko:K01083 | ko00000,ko00001,ko01000 | - | EGF_2,Phytase |
| contig00012.g7426 | FVEG_06038 | 491 | S | - | - | - | - | Hyphal_reg_CWP |
| contig00012.g7434 | FVEG_06027 | 837 | E | 3.5.1.16 | ko:K01438 | ko00000,ko00001,ko00002,ko01000 | - | M20_dimer,Peptidase_M20 |
| contig00012.g7514 | FVEG_05948 | 437 | - | 3.2.1.4 | ko:K01179,ko:K10352 | ko00000,ko00001,ko01000,ko04147,ko04812 | GH5,GH9 | Cellulase,GLEYA,Ricin_B_lectin |
| contig00012.g7522 | FVEG_05942 | 726 | G | - | - | - | - | LysM |
| contig00012.g7564 | FVEG_05900 | 507 | - | - | - | - | - | - |
| contig00012.g7565 | FVEG_05899 | 837 | G | 4.1.1.2 | ko:K01569 | ko00000,ko00001,ko01000 | - | Cupin_1 |
| contig00012.g7566 | FVEG_05898 | 947 | S | - | ko:K17285 | ko00000,ko04147 | - | SBP56 |
| contig00012.g7590 | FVEG_05871 | 565 | O | 3.2.1.73 | ko:K01216 | ko00000,ko01000 | - | Glyco_hydro_16 |
| contig00012.g7595 | FVEG_05867 | 650 | - | - | - | - | - | - |
| contig00012.g7612 | FVEG_05849 | 535 | S | - | - | - | - | Candida_ALS |
| contig00012.g7620 | FVEG_05840 | 370 | - | - | - | - | - | - |
| contig00012.g7638 | FVEG_05821 | 350 | - | - | - | - | - | - |
| contig00012.g7644 | FVEG_05815 | 453 | - | - | - | - | - | - |
| contig00012.g7654 | FVEG_05803 | 1641 | GO | - | - | - | - | DUF1996,WSC |
| contig00012.g7656 | FVEG_05801 | 901 | G | 3.2.1.67 | ko:K01213 | ko00000,ko00001,ko00002,ko01000 | - | Glyco_hydro_28 |
| contig00013.g7718 | FVEG_11317 | 595 | O | - | - | - | - | Asp |
| contig00013.g7753 | FVEG_11284 | 720 | S | - | - | - | - | Ferritin_2 |
| contig00013.g7763 | FVEG_11273 | 572 | S | - | - | - | - | - |
| contig00013.g7801 | FVEG_11233 | 634 | G | 4.2.2.2 | ko:K01728 | ko00000,ko00001,ko01000 | - | CBM_1,Pec_lyase_C |
| contig00013.g7802 | FVEG_11232 | 1555 | S | - | - | - | - | PAN_4 |
| contig00013.g7806 | FVEG_11228 | 3992 | G | 3.1.1.11 | ko:K01051 | ko00000,ko00001,ko00002,ko01000 | - | Pectinesterase |
| contig00013.g7811 | FVEG_11221 | 1063 | E | - | - | - | - | peroxidase |
| contig00013.g7835 | FVEG_11198 | 1003 | C | - | - | - | - | BBE,FAD_binding_4 |
| contig00013.g7836 | FVEG_11197 | 234 | S | - | - | - | - | CAP,DPBB_1 |
| contig00013.g7854 | FVEG_11173 | 451 | - | - | - | - | - | Cerato-platanin |
| contig00013.g7874 | FVEG_11151 | 280 | - | - | - | - | - | - |
| contig00013.g7890 | FVEG_11137 | 685 | Q | 1.14.18.1 | ko:K00505 | ko00000,ko00001,ko00002,ko01000 | - | Tyrosinase |
| contig00013.g7900 | FVEG_11127 | 1089 | O | 3.4.16.6 | ko:K01288 | ko00000,ko01000,ko01002 | - | Peptidase_S10 |
| contig00013.g7902 | FVEG_11125 | 705 | I | - | - | - | - | PAF-AH_p_II |
| contig00013.g7914 | FVEG_11115 | 332 | - | - | - | - | - | - |
| contig00013.g7924 | FVEG_11103 | 266 | - | - | - | - | - | - |
| contig00013.g7934 | FVEG_11094 | 736 | G | - | - | - | - | His_Phos_1 |
| contig00013.g7939 | FVEG_11087 | 598 | O | - | - | - | - | Glyco_hydro_cc |
| contig00013.g7942 | FVEG_11083 | 909 | O | - | - | - | - | Asp |
| contig00013.g7965 | FVEG_11065 | 300 | - | - | - | - | - | - |
| contig00013.g7969 | FVEG_11061 | 598 | P | 4.2.1.1 | ko:K01674 | ko00000,ko00001,ko01000 | - | Carb_anhydrase |
| contig00013.g7976 | FVEG_11053 | 259 | - | - | - | - | - | - |
| contig00013.g8003 | FVEG_11031 | 861 | O | - | - | - | - | Abhydrolase_6,DUF1100 |
| contig00013.g8010 | FVEG_16950 | 294 | - | - | - | - | - | - |
| contig00013.g8017 | FVEG_11018^a^ | 265 | - | - | - | - | - | - |
| contig00013.g8024 | FVEG_11011 | 573 | S | - | - | - | - | GLEYA,PAN_1 |
| contig00013.g8054 | FVEG_10979 | 947 | S | 1.21.99.1 | ko:K15393 | ko00000,ko01000,ko01008 | - | Amino_oxidase,FAD_oxidored,NAD_binding_8 |
| contig00013.g8068 | FVEG_10964 | 901 | S | - | - | - | - | ARID,NACHT,SesA |
| contig00014.g8085 | FVEG_07357 | 1107 | O | - | ko:K08783 | ko00000,ko01000,ko01002 | - | Peptidase_M14 |
| contig00014.g8108 | FVEG_07382 | 522 | S | - | - | - | - | DUF2990,DUF3455 |
| contig00014.g8155 | FVEG_07432 | 712 | O | - | - | - | - | Dioxygenase_C |
| contig00014.g8164 | FVEG_07441 | 828 | S | 1.14.18.1 | ko:K00505 | ko00000,ko00001,ko00002,ko01000 | - | Tyrosinase |
| contig00014.g8170 | FVEG_07448 | 1298 | G | - | - | - | - | Amidohydro_3 |
| contig00014.g8199 | FVEG_07478 | 1425 | S | - | - | - | - | Peptidase_S41 |
| contig00014.g8210 | FVEG_07490 | 683 | G | - | - | - | - | Glyco_hydro_43 |
| contig00014.g8216 | FVEG_07496 | 1137 | - | - | - | - | - | - |
| contig00014.g8231 | FVEG_07510 | 493 | P | 1.15.1.1 | ko:K04564 | ko00000,ko00001,ko01000 | - | Sod_Fe_C,Sod_Fe_N |
| contig00014.g8232 | FVEG_07511 | 812 | - | - | - | - | - | - |
| contig00014.g8240 | FVEG_07518 | 862 | G | 3.2.1.22 | ko:K07407 | ko00000,ko00001,ko01000 | - | Melibiase_2 |
| contig00014.g8246 | FVEG_16133 | 461 | - | - | - | - | - | - |
| contig00014.g8293 | FVEG_07571 | 1019 | S | - | - | - | - | Abhydrolase_1,Abhydrolase_4 |
| contig00014.g8294 | FVEG_07572 | 299 | - | - | - | - | - | - |
| contig00014.g8312 | FVEG_07588 | 417 | G | - | - | - | - | LysM |
| contig00014.g8313 | FVEG_16144 | 57.8 | G | - | - | - | - | DUF1080,LysM |
| contig00014.g8328 | FVEG_07607 | 903 | O | - | - | - | - | Peptidase_M14 |
| contig00014.g8377 | FVEG_07653 | 711 | O | 3.4.11.10 | ko:K05994 | ko00000,ko01000,ko01002 | - | Peptidase_M28 |
| contig00014.g8381 | FVEG_07656 | 565 | S | - | - | - | - | Flocculin,GLEYA |
| contig00014.g8382 | FVEG_07657 | 681 | S | 1.14.18.1 | ko:K00505 | ko00000,ko00001,ko00002,ko01000 | - | Tyrosinase |
| contig00014.g8389 | FVEG_07663 | 204 | S | - | - | - | - | NPP1 |
| contig00014.g8390 | FVEG_07664 | 365 | - | - | - | - | - | - |
| contig00014.g8392 | FVEG_07665 | 234 | - | - | - | - | - | PAN_1,PAN_4 |
| contig00014.g8405 | FVEG_07677 | 1202 | O | 3.4.14.9 | ko:K01279 | ko00000,ko00001,ko01000,ko01002,ko03110,ko04147 | - | Peptidase_S8,Pro-kuma_activ |
| contig00014.g8406 | FVEG_07678 | 644 | S | - | - | - | - | Lipase_GDSL |
| contig00014.g8412 | FVEG_16167 | 1720 | - | - | - | - | - | - |
| contig00014.g8415 | FVEG_07688 | 836 | G | 5.1.3.3 | ko:K01785 | ko00000,ko00001,ko00002,ko01000 | - | Aldose_epim |
| contig00014.g8417 | FVEG_07690 | 462 | S | - | - | - | - | Candida_ALS,GLEYA |
| contig00014.g8423 | FVEG_07695 | 200 | S | - | - | - | - | Hydrophobin_2 |
| contig00014.g8431 | FVEG_07702 | 901 | O | - | - | - | - | Glyco_hydro_39 |
| contig00014.g8443 | FVEG_07712 | 973 | O | 3.4.16.6 | ko:K01288 | ko00000,ko01000,ko01002 | - | Peptidase_S10 |
| contig00014.g8447 | FVEG_07716 | 1476 | G | 3.2.1.22 | ko:K07407 | ko00000,ko00001,ko01000 | - | Glyco_hydro_36C,Glyco_hydro_36N,Melibiase |
| contig00014.g8449 | FVEG_07718 | 1130 | O | - | - | - | - | BBE,FAD_binding_4 |
| contig00014.g8460 | FVEG_07728 | 494 | - | - | - | - | - | CBM_4_9 |
| contig00014.g8462 | FVEG_07730 | 699 | O | 3.1.1.72 | ko:K05972 | ko00000,ko01000 | - | CBM_1,Esterase_phd |
| contig00014.g8481 | FVEG_07747 | 430 | V | - | - | - | - | - |
| contig00014.g8483 | FVEG_07750 | 323 | - | - | - | - | - | - |
| contig00014.g8485 | FVEG_07752 | 250 | - | - | - | - | - | - |
| contig00014.g8487 | FVEG_07753 | 450 | G | - | - | - | - | Cellulase,RicinB_lectin_2,Ricin_B_lectin |
| contig00014.g8496 | FVEG_07763 | 1816 | S | - | - | - | - | Clr5,MU117,TPR_12 |
| contig00015.g8551 | FVEG_11681 | 352 | - | - | - | - | - | - |
| contig00015.g8562 | FVEG_07833 | 485 | - | - | - | - | - | - |
| contig00015.g8619 | FVEG_07889 | 1315 | P | - | - | - | - | Alk_phosphatase |
| contig00015.g8628 | FVEG_16208 | 192 | - | - | - | - | - | - |
| contig00015.g8709 | FVEG_07981 | 730 | O | 5.3.4.1 | ko:K09584 | ko00000,ko00001,ko01000,ko03110,ko04131 | - | ERp29,Thioredoxin |
| contig00016.g8870 | FVEG_09484 | 316 | O | 5.2.1.8 | ko:K09569,ko:K09577 | ko00000,ko01000,ko03110 | - | FKBP_C |
| contig00016.g8929 | FVEG_09545 | 411 | S | - | - | - | - | EF-hand_5,EF-hand_6,EF-hand_7 |
| contig00016.g8931 | FVEG_09547 | 613 | - | - | - | - | - | - |
| contig00016.g8991 | FVEG_09611 | 762 | C | 1.1.2.3 | ko:K00101 | ko00000,ko00001,ko01000 | - | FMN_dh |
| contig00016.g9028 | FVEG_09653 | 479 | G | 3.1.1.74 | ko:K08095 | ko00000,ko01000 | - | Cutinase |
| contig00016.g9069 | FVEG_09694 | 1162 | G | - | - | - | - | COesterase |
| contig00016.g9076 | FVEG_09702 | 743 | G | 4.2.2.10 | ko:K01732 | ko00000,ko01000 | - | Pec_lyase_C |
| contig00016.g9087 | FVEG_09713 | 647 | O | - | - | - | - | LicD |
| contig00016.g9093 | FVEG_09719 | 682 | G | 3.2.1.4 | ko:K01179 | ko00000,ko00001,ko01000 | GH5,GH9 | CBM_1,Cellulase |
| contig00016.g9130 | FVEG_09758 | 253 | - | - | - | - | - | - |
| contig00016.g9143 | FVEG_09770 | 534 | - | - | - | - | - | - |
| contig00016.g9144 | FVEG_09772 | 1274 | G | - | - | - | - | Glyco_hydro_1 |
| contig00016.g9151 | FVEG_09778 | 409 | - | - | - | - | - | - |
| contig00016.g9160 | FVEG_05913 | 209 | - | - | - | - | - | CVNH |
| contig00017.g9171 | FVEG_17099 | 966 | T | - | - | - | - | COesterase |
| contig00017.g9186 | FVEG_11796 | 996 | GO | - | - | - | - | DUF1996,WSC |
| contig00017.g9195 | FVEG_11787 | 695 | G | 3.2.1.15,3.2.1.67 | ko:K01184,ko:K01213 | ko00000,ko00001,ko00002,ko01000 | - | Glyco_hydro_28 |
| contig00017.g9210 | FVEG_11773 | 1180 | - | - | - | - | - | - |
| contig00017.g9211 | FVEG_11772 | 1289 | E | 1.1.99.1 | ko:K00108 | ko00000,ko00001,ko00002,ko01000 | - | GMC_oxred_C,GMC_oxred_N |
| contig00017.g9214 | FVEG_11768 | 830 | I | - | - | - | - | Abhydrolase_6,Hydrolase_4 |
| contig00017.g9219 | FVEG_11764 | 1626 | Q | 1.4.3.21 | ko:K00276 | ko00000,ko00001,ko01000 | - | Cu_amine_oxid,Cu_amine_oxidN2,DUF1965 |
| contig00017.g9234 | FVEG_11749 | 1573 | G | 3.2.1.21 | ko:K05349 | ko00000,ko00001,ko01000 | GH3 | Fn3-like,Glyco_hydro_3,Glyco_hydro_3_C |
| contig00017.g9246 | FVEG_11738 | 493 | - | - | - | - | - | - |
| contig00017.g9247 | FVEG_11737 | 254 | - | - | - | - | - | - |
| contig00017.g9253 | FVEG_11731 | 992 | C | - | - | - | - | BBE,FAD_binding_4 |
| contig00017.g9262 | FVEG_17080 | 135 | - | - | - | - | - | - |
| contig00017.g9263 | FVEG_11723 | 235 | - | - | - | - | - | - |
| contig00017.g9264 | FVEG_11722 | 436 | - | - | - | - | - | - |
| contig00017.g9266 | FVEG_11720 | 315 | - | - | - | - | - | - |
| contig00017.g9269 | FVEG_11717 | 291 | G | - | - | - | - | Amidohydro_1 |
| contig00017.g9272 | FVEG_11714 | 1028 | Q | 1.5.3.14,1.5.3.16 | ko:K13366 | ko00000,ko00001,ko01000 | - | Amino_oxidase |
| contig00017.g9286 | FVEG_11700 | 621 | O | 3.1.1.72 | ko:K05972 | ko00000,ko01000 | - | CBM_1,Esterase_phd |
| contig00017.g9291 | FVEG_11695 | 1571 | O | 3.4.14.5 | ko:K01278,ko:K01282 | ko00000,ko00001,ko01000,ko01002,ko04090,ko04147 | - | DPPIV_N,Peptidase_S9 |
| contig00017.g9300 | FVEG_11689 | 759 | S | - | - | - | - | GLEYA |
| contig00017.g9324 | FVEG_11667 | 441 | - | - | - | - | - | - |
| contig00017.g9325 | FVEG_11666 | 640 | - | - | - | - | - | - |
| contig00017.g9326 | FVEG_11665 | 402 | - | - | - | - | - | - |
| contig00017.g9370 | FVEG_11609 | 203 | - | - | - | - | - | - |
| contig00017.g9380 | FVEG_11600 | 500 | G | - | ko:K20628 | ko00000 | - | DPBB_1,Pollen_allerg_1 |
| contig00017.g9486 | FVEG_11492 | 811 | O | - | - | - | - | Asp |
| contig00017.g9521 | FVEG_11455 | 225 | - | - | - | - | - | - |
| contig00017.g9523 | FVEG_11453 | 789 | O | 3.4.23.25 | ko:K01381 | ko00000,ko00001,ko01000,ko01002 | - | Asp |
| contig00018.g9577 | FVEG_12142 | 644 | G | 3.2.1.58 | ko:K01210 | ko00000,ko00001,ko01000 | - | Glyco_hydro_17 |
| contig00018.g9582 | FVEG_12137 | 2893 | G | - | ko:K11718 | ko00000,ko00001,ko01000,ko01003 | GT24 | Glyco_transf_8,UDP-g_GGTase |
| contig00018.g9590 | FVEG_12127 | 2150 | - | - | ko:K19720 | ko00000,ko00001,ko00536 | - | - |
| contig00018.g9593 | FVEG_12124 | 640 | G | - | - | - | - | Exo_endo_phos |
| contig00018.g9607 | FVEG_12110 | 781 | S | 3.1.1.2,3.1.8.1 | ko:K01045 | ko00000,ko01000,ko04147 | - | Arylesterase |
| contig00018.g9611 | FVEG_12107 | 459 | - | - | - | - | - | CBM9_2 |
| contig00018.g9624 | FVEG_12093 | 479 | O | 4.4.1.5 | ko:K01759 | ko00000,ko00001,ko01000 | - | Glyoxalase |
| contig00018.g9639 | FVEG_12078 | 972 | - | - | - | - | - | - |
| contig00018.g9694 | FVEG_12024 | 809 | G | 3.5.3.11 | ko:K01480 | ko00000,ko00001,ko00002,ko01000 | - | Arginase |
| contig00018.g9698 | FVER14953_20274^b^ | 262 | - | - | - | - | - | - |
| contig00018.g9705 | FVEG_12015 | 763 | S | - | - | - | - | Abhydrolase_6 |
| contig00018.g9711 | FVEG_12008 | 699 | - | - | - | - | - | - |
| contig00018.g9717 | FVEG_12001 | 358 | - | - | - | - | - | - |
| contig00018.g9731 | FVEG_11988 | 769 | V | - | - | - | - | - |
| contig00018.g9767 | FVEG_11952 | 2873 | S | - | - | - | - | Lipase_GDSL_2,MU117,Spherulin4,VCBS |
| contig00018.g9772 | FVEG_11948 | 1233 | Q | 1.10.3.3 | ko:K00423 | ko00000,ko00001,ko01000 | - | Cu-oxidase,Cu-oxidase_2,Cu-oxidase_3 |
| contig00018.g9777 | FVEG_11944 | 621 | O | - | - | - | - | Glyco_hydro_16 |
| contig00018.g9788 | FVEG_11934 | 786 | O | - | - | - | - | Pyr_redox_2 |
| contig00018.g9790 | FVEG_11933 | 1102 | G | 3.2.1.26 | ko:K01193 | ko00000,ko00001,ko01000 | GH32 | Glyco_hydro_32C,Glyco_hydro_32N |
| contig00018.g9806 | FVEG_11917 | 760 | - | - | - | - | - | - |
| contig00018.g9812 | FVEG_11911 | 404 | - | - | - | - | - | - |
| contig00018.g9819 | FVEG_11904 | 491 | - | - | - | - | - | - |
| contig00018.g9822 | FVEG_11902 | 1604 | - | - | - | - | - | - |
| contig00018.g9823 | FVEG_11901 | 1135 | E | - | - | - | - | GMC_oxred_C,GMC_oxred_N |
| contig00018.g9832 | FVEG_11891 | 889 | S | - | - | - | - | Abhydrolase_6 |
| contig00018.g9834 | FVEG_11889 | 840 | G | - | - | - | - | Tannase |
| contig00018.g9842 | FVEG_11882 | 798 | O | - | - | - | - | Asp |
| contig00018.g9849 | FVEG_11874 | 973 | S | - | - | - | - | His_Phos_2 |
| contig00018.g9854 | FVEG_11869 | 555 | - | - | - | - | - | CBM_1,Lipase_GDSL,Lipase_GDSL_2 |
| contig00018.g9856 | FVEG_11867 | 691 | G | 3.2.1.4 | ko:K01179 | ko00000,ko00001,ko01000 | GH5,GH9 | CBM_1,Cellulase |
| contig00018.g9871 | FVEG_17120 | 326 | - | - | - | - | - | - |
| contig00018.g9874 | FVEG_11851 | 539 | - | - | - | - | - | - |
| contig00018.g9878 | FVEG_11849 | 773 | G | 3.2.1.8 | ko:K01181 | ko00000,ko01000 | - | CBM_1,Glyco_hydro_10 |
| contig00018.g9903 | FVEG_11827 | 252 | - | - | - | - | - | - |
| contig00018.g9906 | FVEG_11824 | 759 | S | 4.1.1.52 | ko:K22213 | ko00000,ko01000 | - | Amidohydro_2 |
| contig00018.g9920 | FVEG_11810 | 153 | S | - | - | - | - | RALF |
| contig00019.g10004 | FVEG_12435 | 946 | - | - | - | - | - | - |
| contig00019.g10005 | FVEG_12434 | 485 | G | - | - | - | - | Pectate_lyase |
| contig00019.g10014 | FVEG_12423 | 902 | G | 3.2.1.78 | ko:K19355 | ko00000,ko00001,ko01000 | - | CBM_1,Cellulase |
| contig00019.g10015 | FVEG_12422 | 316 | - | - | - | - | - | - |
| contig00019.g10034 | FVEG_12404 | 407 | E | 1.14.18.1 | ko:K00505 | ko00000,ko00001,ko00002,ko01000 | - | Tyrosinase |
| contig00019.g10037 | FVEG_12400 | 984 | S | - | ko:K19851,ko:K22493 | ko00000,ko00001,ko00537 | - | Candida_ALS,Candida_ALS_N,Flo11 |
| contig00019.g10038 | FVEG_12399 | 780 | - | - | - | - | - | - |
| contig00019.g10040 | FVEG_12397 | 234 | - | - | - | - | - | - |
| contig00019.g10041 | FVEG_12396 | 223 | - | - | - | - | - | - |
| contig00019.g10051 | FVEG_12387 | 512 | - | - | - | - | - | - |
| contig00019.g10058 | FVEG_12381 | 920 | M | - | - | - | - | Asp |
| contig00019.g10066 | FVEG_12372 | 1144 | E | - | - | - | - | GMC_oxred_C,GMC_oxred_N |
| contig00019.g10072 | FVEG_12368 | 1365 | E | 4.2.2.23 | ko:K18195 | ko00000,ko01000 | PL4 | CBM-like,CarboxypepD_reg,fn3_3 |
| contig00019.g10088 | FVEG_12354 | 288 | - | - | - | - | - | - |
| contig00019.g10089 | FVEG_12353 | 1062 | O | - | - | - | - | Peptidase_M14 |
| contig00019.g10097 | FVEG_12346 | 428 | O | 3.1.1.74 | ko:K08095 | ko00000,ko01000 | - | Cutinase |
| contig00019.g10098 | FVEG_12345 | 1467 | G | 3.2.1.155 | ko:K18578 | ko00000,ko01000 | CBM1,GH74 | CBM_1 |
| contig00019.g10111 | FVEG_12331 | 678 | - | - | - | - | - | - |
| contig00019.g10112 | FVEG_12330 | 281 | S | - | - | - | - | Cerato-platanin |
| contig00019.g10118 | FVEG_12324 | 458 | - | - | - | - | - | LysM |
| contig00019.g10133 | FVEG_12315 | 514 | S | - | - | - | - | - |
| contig00019.g10150 | FVEG_12299 | 765 | G | - | - | - | - | Cellulase |
| contig00019.g10154 | FVEG_12295 | 253 | S | 3.1.27.3 | ko:K01167 | ko00000,ko01000,ko03016,ko03019 | - | Ribonuclease |
| contig00019.g10160 | FVEG_12289 | 1113 | G | 3.2.1.22 | ko:K07407 | ko00000,ko00001,ko01000 | - | Melibiase_2 |
| contig00019.g10164 | FVEG_12285 | 937 | C | - | - | - | - | FAD_binding_4 |
| contig00019.g10165 | FVEG_12284 | 182 | - | - | - | - | - | - |
| contig00019.g10167 | FVEG_12281 | 821 | - | - | - | - | - | - |
| contig00019.g10168 | FVEG_12280 | 466 | O | 3.4.21.4 | ko:K01312 | ko00000,ko00001,ko01000,ko01002,ko04147 | - | Trypsin |
| contig00019.g10169 | FVEG_17206 | 70.9 | - | - | - | - | - | - |
| contig00019.g10170 | FVEG_12279 | 619 | H | 4.2.2.2 | ko:K01728 | ko00000,ko00001,ko01000 | - | Pec_lyase_C |
| contig00019.g10171 | FVEG_12278 | 946 | S | - | - | - | - | - |
| contig00019.g10206 | FVEG_12245 | 547 | S | - | - | - | - | Hce2 |
| contig00019.g10207 | FVEG_12244 | 769 | O | - | - | - | - | Peptidase_S8 |
| contig00019.g10213 | FVEG_12238 | 1274 | E | 1.1.99.1 | ko:K00108 | ko00000,ko00001,ko00002,ko01000 | - | GMC_oxred_C,GMC_oxred_N |
| contig00019.g10216 | FVEG_12236 | 909 | S | - | - | - | - | - |
| contig00019.g10217 | FVEG_12235 | 835 | S | 1.11.2.1 | ko:K21820 | ko00000,ko01000 | - | Peroxidase_2 |
| contig00019.g10241 | FVEG_12213 | 659 | G | - | ko:K20465 | ko00000,ko04131 | - | UDPGT |
| contig00019.g10242 | FVEG_12212 | 450 | S | - | - | - | - | Peroxidase_2 |
| contig00019.g10245 | FVEG_12209 | 921 | G | 4.2.2.10 | ko:K01732 | ko00000,ko01000 | - | CBM_1,Pec_lyase_C |
| contig00019.g10250 | FVEG_12203 | 746 | S | - | ko:K07140 | ko00000 | - | MOSC,MOSC_N |
| contig00019.g10251 | FVEG_12202 | 927 | S | - | - | - | - | Amino_oxidase,NAD_binding_8 |
| contig00019.g10261 | FVEG_12194 | 818 | O | - | - | - | - | Peptidase_M14,Propep_M14 |
| contig00019.g10266 | FVEG_12189 | 1232 | G | 3.2.1.51 | ko:K01206 | ko00000,ko00001,ko01000,ko04147 | GH29 | Alpha_L_fucos,Fucosidase_C |
| contig00019.g10268 | FVEG_17178 | 847 | S | - | - | - | - | BNR_4 |
| contig00019.g10274 | FVEG_12185 | 509 | P | 3.1.3.1 | ko:K01113 | ko00000,ko00001,ko00002,ko01000 | - | PhoD,PhoD_N |
| contig00019.g10278 | FVEG_12180 | 496 | O | - | - | - | - | CBM_1,Lipase_GDSL_2 |
| contig00019.g10282 | FVEG_12176 | 1761 | - | - | - | - | - | - |
| contig00019.g9935 | FVEG_12502 | 734 | G | 3.2.1.8 | ko:K01181 | ko00000,ko01000 | - | Glyco_hydro_10 |
| contig00019.g9954 | FVEG_12486 | 486 | G | 3.2.1.151 | ko:K18576 | ko00000,ko01000 | GH12 | Glyco_hydro_12 |
| contig00019.g9955 | FVEG_12485 | 565 | O | - | - | - | - | CBM_1,Esterase,Peptidase_S9 |
| contig00019.g9956 | FVEG_12484 | 1474 | - | - | - | - | - | PAN_1,PAN_4 |
| contig00019.g9957 | FVEG_12483 | 634 | - | - | - | - | - | - |
| contig00019.g9958 | FVEG_12482 | 843 | G | - | - | - | - | - |
| contig00019.g9981 | FVEG_17275 | 120 | S | - | - | - | - | Cerato-platanin |
| contig00019.g9982 | FVEG_12459 | 278 | - | - | - | - | - | - |
| contig00019.g9992 | FVEG_12448 | 289 | - | - | - | - | - | - |
| contig00019.g9995 | FVEG_12445 | 645 | P | 4.2.1.1 | ko:K01674 | ko00000,ko00001,ko01000 | - | Carb_anhydrase |
| contig00019.g9999 | FVEG_12442 | 1873 | GO | 1.2.3.15 | ko:K20929 | ko00000,ko01000 | - | DUF1929,Glyoxal_oxid_N,WSC |
| contig00020.g10296 | FVEG_12805 | 859 | S | 3.1.4.46 | ko:K01126 | ko00000,ko00001,ko01000 | - | GDPD |
| contig00020.g10297 | FVEG_12804 | 740 | G | 3.2.1.14 | ko:K01183 | ko00000,ko00001,ko01000 | GH18 | Glyco_hydro_18 |
| contig00020.g10323 | FVEG_12780 | 791 | F | - | - | - | - | NUP |
| contig00020.g10333 | FVEG_12770 | 1197 | S | - | ko:K19851,ko:K22493 | ko00000,ko00001,ko00537 | - | CFEM,Candida_ALS,Candida_ALS_N,Flo11 |
| contig00020.g10338 | FVEG_12766 | 821 | G | - | - | - | - | Pectinesterase |
| contig00020.g10339 | FVEG_12765 | 408 | S | - | - | - | - | - |
| contig00020.g10360 | FVEG_12744 | 949 | G | 3.2.1.67 | ko:K01213 | ko00000,ko00001,ko00002,ko01000 | - | Glyco_hydro_28 |
| contig00020.g10370 | FVEG_12733 | 734 | O | - | - | - | - | Pyr_redox_2 |
| contig00020.g10375 | FVEG_12728 | 452 | - | - | - | - | - | - |
| contig00020.g10381 | FVEG_12722 | 764 | G | 3.2.1.78 | ko:K19355 | ko00000,ko00001,ko01000 | - | Cellulase |
| contig00020.g10384 | FVEG_12719 | 862 | - | - | - | - | - | - |
| contig00020.g10387 | FVEG_12716 | 281 | - | - | - | - | - | - |
| contig00020.g10401 | FVEG_12704 | 1785 | G | - | - | - | - | Chitin_bind_1,Glyco_hydro_18,LysM |
| contig00020.g10406 | FVEG_17330 | 2816 | S | - | - | - | - | Lipase_GDSL_2,VCBS |
| contig00020.g10421 | FVEG_12682 | 253 | - | - | - | - | - | - |
| contig00020.g10435 | FVEG_12666 | 666 | O | - | - | - | - | Glyco_hydro_62 |
| contig00020.g10456 | FVEG_12643 | 853 | G | 3.2.1.14 | ko:K01183 | ko00000,ko00001,ko01000 | GH18 | Chitin_bind_1,Glyco_hydro_18 |
| contig00020.g10482 | FVEG_12617 | 1769 | S | - | - | - | - | Ank_2,Ank_4 |
| contig00020.g10484 | FVEG_12615 | 1407 | O | - | - | - | - | Amino_oxidase |
| contig00020.g10500 | FVEG_12602 | 45.1 | - | - | - | - | - | - |
| contig00020.g10521 | FVEG_12585 | 1123 | M | - | - | - | - | Ank,Ank_2,Ank_3,Ank_4,HeLo,NACHT |
| contig00020.g10554 | FVEG_12553 | 1176 | G | 3.2.1.51 | ko:K01206 | ko00000,ko00001,ko01000,ko04147 | GH29 | Alpha_L_fucos |
| contig00020.g10561 | FVEG_12546 | 1043 | O | - | - | - | - | Peptidase_M14 |
| contig00020.g10568 | FVEG_12539 | 943 | I | - | - | - | - | Abhydrolase_6,Hydrolase_4 |
| contig00020.g10581 | FVEG_12526 | 676 | S | - | - | - | - | Lactamase_B |
| contig00020.g10583 | FVEG_12524 | 976 | G | - | - | - | - | COesterase |
| contig00021.g10727 | FVEG_01870 | 1706 | G | 3.2.1.6 | ko:K01180 | ko00000,ko01000 | - | Glyco_hydro_81 |
| contig00021.g10778 | FVEG_01819 | 969 | G | - | - | - | - | COesterase |
| contig00021.g10794 | FVEG_01804 | 671 | O | 3.4.24.39 | ko:K19305 | ko00000,ko01000,ko01002 | - | Aspzincin_M35,Peptidase_M35 |
| contig00021.g10801 | FVEG_01795 | 936 | S | - | - | - | - | Beta-lactamase |
| contig00021.g10820 | FVEG_01779 | 883 | G | 3.2.1.75 | ko:K22277 | ko00000,ko01000 | GH5 | Cellulase |
| contig00022.g10823 | FVEG_16810 | 1070 | G | - | - | - | - | COesterase,TB2_DP1_HVA22 |
| contig00022.g10864 | FVEG_16817 | 164 | S | - | - | - | - | Antifungal_prot |
| contig00022.g10866 | FVEG_16818 | 102 | - | - | - | - | - | - |
| contig00022.g10881 | FVEG_10538 | 744 | S | 4.2.1.95 | ko:K20613 | ko00000,ko01000 | - | CrtC,Lipocalin_9 |
| contig00022.g10885 | FVEG_10542 | 503 | S | - | - | - | - | Cupin_1 |
| contig00022.g10887 | FVEG_10544 | 637 | - | - | - | - | - | - |
| contig00022.g10907 | FVEG_10558 | 955 | O | - | - | - | - | PA,PQ-loop,Peptidase_M28 |
| contig00022.g10921 | FVEG_10576 | 155 | - | - | - | - | - | - |
| contig00022.g10924 | FVEG_10579 | 226 | - | - | - | - | - | - |
| contig00022.g10928 | FVEG_10582 | 815 | S | - | - | - | - | Peroxidase_2 |
| contig00022.g10930 | FVEG_10584 | 1302 | E | 1.1.99.1 | ko:K00108 | ko00000,ko00001,ko00002,ko01000 | - | GMC_oxred_C,GMC_oxred_N,NAD_binding_8 |
| contig00022.g10950 | FVEG_10625 | 694 | G | - | - | - | - | Glyco_hydro_43,Laminin_G_3 |
| contig00022.g10951 | FVEG_10624 | 646 | G | 3.2.1.99 | ko:K06113 | ko00000,ko01000 | GH43 | Glyco_hydro_43 |
| contig00022.g10971 | FVEG_10605 | 1639 | G | - | - | - | - | Glyco_hydro_79C |
| contig00022.g10974 | FVEG_10602 | 1057 | O | - | - | - | - | Peptidase_M14 |
| contig00022.g10976 | FVEG_10600 | 501 | - | - | - | - | - | - |
| contig00022.g10992 | FVEG_10647 | 662 | C | 1.14.13.1 | ko:K00480 | ko00000,ko00001,ko01000 | - | FAD_binding_3,NAD_binding_8 |
| contig00022.g11020 | FVEG_10673 | 1102 | G | - | - | - | - | Tannase |
| contig00022.g11086 | FVEG_10735 | 791 | O | - | - | - | - | - |
| contig00022.g11088 | FVEG_10738 | 1860 | - | - | - | - | - | - |
| contig00022.g11090 | FVEG_10740 | 1056 | S | - | - | - | - | Beta-lactamase |
| contig00022.g11095 | FVEG_10744 | 678 | G | - | - | - | - | Glyco_hydro_43 |
| contig00022.g11098 | FVEG_10747 | 385 | - | - | - | - | - | But2 |
| contig00023.g11119 | FVEG_14140 | 1224 | E | 1.1.99.1,4.2.1.142,4.2.1.143 | ko:K00108,ko:K17646 | ko00000,ko00001,ko00002,ko01000 | - | GMC_oxred_C,GMC_oxred_N |
| contig00023.g11123 | FVEG_14136 | 1843 | G | 3.2.1.20 | ko:K01187 | ko00000,ko00001,ko01000 | GH31 | Gal_mutarotas_2,Glyco_hydro_31,NtCtMGAM_N |
| contig00023.g11142 | FVEG_14117 | 284 | - | - | - | - | - | - |
| contig00023.g11147 | FVEG_01522 | 472 | C | - | - | - | - | ADH_N,ADH_zinc_N |
| contig00023.g11169 | FVEG_01544 | 353 | - | - | - | - | - | - |
| contig00023.g11172 | FVEG_01546 | 757 | P | - | - | - | - | SBP_bac_1,SBP_bac_6 |
| contig00023.g11173 | FVEG_01547 | 506 | S | - | - | - | - | DUF3129 |
| contig00023.g11178 | FVEG_01553 | 632 | O | - | - | - | - | - |
| contig00023.g11179 | FVEG_01554 | 294 | - | - | - | - | - | - |
| contig00023.g11182 | FVEG_01557 | 746 | O | 3.4.11.10 | ko:K05994 | ko00000,ko01000,ko01002 | - | Peptidase_M28 |
| contig00023.g11183 | FVEG_14861 | 2622 | M | - | - | - | - | Ank_2,Ank_4,NACHT |
| contig00023.g11194 | FVEG_01567 | 946 | S | - | - | - | - | LVIVD |
| contig00023.g11203 | FVEG_01575 | 202 | S | - | - | - | - | Hydrophobin_2 |
| contig00023.g11212 | FVEG_01583 | 3634 | G | 3.2.1.14 | ko:K01183 | ko00000,ko00001,ko01000 | GH18 | Chitin_bind_1,Glyco_hydro_18 |
| contig00023.g11213 | FVEG_01584 | 1504 | O | - | - | - | - | Chitin_bind_1,LysM |
| contig00023.g11225 | FVEG_01594 | 1403 | - | - | - | - | - | - |
| contig00023.g11228 | FVEG_01596 | 479 | - | - | - | - | - | - |
| contig00023.g11231 | FVEG_01598 | 215 | - | - | - | - | - | - |
| contig00023.g11247 | FVEG_01614 | 773 | S | 1.14.18.1 | ko:K00505 | ko00000,ko00001,ko00002,ko01000 | - | Tyrosinase |
| contig00023.g11248 | FVEG_01615 | 348 | - | - | - | - | - | - |
| contig00023.g11257 | FVEG_01622 | 536 | - | - | - | - | - | - |
| contig00023.g11264 | FVEG_01630 | 419 | S | - | - | - | - | - |
| contig00023.g11280 | FVEG_01647 | 1103 | T | - | ko:K08288 | ko00000,ko00001,ko04091 | - | PRKCSH-like,PRKCSH_1 |
| contig00023.g11292 | FVEG_01659 | 144 | - | - | - | - | - | - |
| contig00023.g11293 | FVEG_01660 | 252 | - | - | - | - | - | - |
| contig00023.g11302 | FVEG_01670 | 812 | C | - | - | - | - | FAD_binding_3 |
| contig00023.g11358 | FVEG_01726 | 306 | S | - | - | - | - | - |
| contig00023.g11361 | FVEG_01729 | 374 | S | - | - | - | - | EthD |
| contig00023.g11363 | FVEG_01731 | 1006 | S | - | ko:K09704 | ko00000 | - | Glyco_hydro_125 |
| contig00023.g11374 | FVEG_01746 | 555 | - | - | - | - | - | PAN_4 |
| contig00023.g11385 | FVEG_01758 | 292 | - | - | - | - | - | - |
| contig00023.g11392 | FVEG_14919 | 902 | Q | - | - | - | - | p450 |
| contig00023.g11396 | FVEG_01770 | 895 | G | 3.2.1.4 | ko:K19357 | ko00000,ko00001,ko01000 | GH7 | Glyco_hydro_7 |
| contig00023.g11406 | FVEG_01778 | 469 | G | 3.2.1.132 | ko:K01233 | ko00000,ko00001,ko01000 | - | Glyco_hydro_75 |
| contig00024.g11452 | FVEG_06297 | 685 | S | - | - | - | - | Fasciclin |
| contig00024.g11586 | FVEG_06439 | 683 | O | - | - | - | - | Glyco_hydro_43 |
| contig00024.g11590 | FVEG_06442 | 1103 | G | - | - | - | - | COesterase,TB2_DP1_HVA22 |
| contig00024.g11608 | FVEG_06459 | 619 | G | 1.14.99.54 | ko:K19356 | ko00000,ko01000 | AA9,CBM1 | CBM_1,Glyco_hydro_61 |
| contig00025.g11645 | FVEG_13584 | 1113 | - | - | - | - | - | - |
| contig00025.g11649 | FVEG_13581 | 1051 | - | - | - | - | - | - |
| contig00025.g11652 | FVEG_13578 | 979 | G | - | - | - | - | Glyco_hydr_30_2,Glyco_hydro_30,Glyco_hydro_30C |
| contig00025.g11663 | FVEG_13565 | 272 | - | - | - | - | - | - |
| contig00025.g11674 | FVEG_13557 | 270 | - | - | - | - | - | - |
| contig00025.g11675 | FVEG_13555 | 1472 | G | 3.1.3.2 | ko:K01078,ko:K22390 | ko00000,ko00001,ko01000 | - | Metallophos,Metallophos_C,Pur_ac_phosph_N |
| contig00025.g11676 | FVEG_13554 | 938 | G | - | - | - | - | Glyco_hydro_30C |
| contig00025.g11677 | FVEG_13553 | 742 | G | - | - | - | - | Glyco_hydro_43 |
| contig00025.g11683 | FVEG_17547 | 3091 | GO | - | - | - | - | LysM,Pectate_lyase_3,WSC |
| contig00025.g11684 | FVEG_17546 | 1969 | G | 3.2.1.14 | ko:K01183 | ko00000,ko00001,ko01000 | GH18 | Chitin_bind_1,Glyco_hydro_18 |
| contig00025.g11694 | FVEG_13534 | 437 | S | 3.1.1.72 | ko:K05972 | ko00000,ko01000 | - | Cutinase |
| contig00025.g11697 | FVEG_13531 | 1040 | V | - | - | - | - | ALO,FAD_binding_4 |
| contig00025.g11707 | FVEG_13522 | 602 | O | 3.1.1.72 | ko:K05972 | ko00000,ko01000 | - | Esterase_phd |
| contig00025.g11713 | FVEG_13516 | 733 | G | 3.2.1.15,3.2.1.67 | ko:K01184,ko:K01213 | ko00000,ko00001,ko00002,ko01000 | - | CBM_1,Glyco_hydro_28 |
| contig00025.g11727 | FVEG_13504 | 515 | O | 1.14.99.54 | ko:K19356 | ko00000,ko01000 | AA9,CBM1 | CBM_1,Glyco_hydro_61 |
| contig00025.g11730 | FVEG_02317 | 95.5 | - | - | - | - | - | - |
| contig00025.g11731 | FVEG_17533 | 1987 | G | 3.2.1.28 | ko:K01194 | ko00000,ko00001,ko00537,ko01000 | GH37 | Glyco_hydro_65C,Glyco_hydro_65N,Glyco_hydro_65m |
| contig00025.g11732 | FVEG_13498 | 300 | - | - | - | - | - | - |
| contig00025.g11736 | FVEG_13495 | 245 | - | - | - | - | - | - |
| contig00025.g11747 | FVEG_13483 | 131 | - | - | - | - | - | - |
| contig00025.g11760 | FVEG_13471 | 695 | - | - | - | - | - | - |
| contig00025.g11768 | FVEG_13463 | 165 | - | - | - | - | - | - |
| contig00025.g11774 | FVEG_13457 | 699 | G | - | - | - | - | CBM_1,Glyco_hydro_16 |
| contig00025.g11775 | FVEG_13456 | 244 | - | - | - | - | - | - |
| contig00025.g11777 | FVEG_13454 | 450 | - | - | - | - | - | - |
| contig00025.g11781 | FVEG_13450 | 970 | C | - | - | - | - | FAD_binding_4 |
| contig00025.g11792 | FVEG_13439 | 313 | - | - | - | - | - | - |
| contig00025.g11793 | FVEG_17524 | 1021 | S | - | - | - | - | Lipase_GDSL_2,VCBS |
| contig00025.g11797 | FVEG_13433 | 560 | O | - | - | - | - | Peptidase_M43 |
| contig00025.g11804 | FVEG_13426 | 745 | G | - | - | - | - | Glyco_hydro_43 |
| contig00025.g11826 | FVEG_13405 | 1359 | Q | - | - | - | - | Cu-oxidase,Cu-oxidase_2,Cu-oxidase_3 |
| contig00025.g11827 | FVEG_13404 | 1718 | O | - | - | - | - | FlgD_ig,PA,Peptidase_S8,fn3_5 |
| contig00025.g11841 | FVEG_13391 | 1550 | G | 3.2.1.21 | ko:K05349 | ko00000,ko00001,ko01000 | GH3 | Fn3-like,Glyco_hydro_3,Glyco_hydro_3_C |
| contig00025.g11869 | FVEG_13364 | 674 | S | - | ko:K06889 | ko00000 | - | Abhydrolase_6 |
| contig00025.g11874 | FVEG_13359 | 1062 | G | - | - | - | - | COesterase |
| contig00025.g11877 | FVEG_13356 | 216 | IQ | - | - | - | - | p450 |
| contig00025.g11882 | FVEG_13350 | 586 | S | - | - | - | - | NPP1 |
| contig00025.g11886 | FVEG_17506 | 204 | - | - | - | - | - | - |
| contig00025.g11887 | FVEG_13346 | 391 | - | - | - | - | - | - |
| contig00025.g11889 | FVEG_13344 | 674 | O | - | - | - | - | Glyco_hydro_61 |
| contig00025.g11890 | FVEG_13343 | 663 | G | 3.2.1.8 | ko:K01181 | ko00000,ko01000 | - | Glyco_hydro_10 |
| contig00025.g11895 | FVEG_13339 | 1168 | G | - | - | - | - | COesterase |
| contig00025.g11908 | FVEG_13328 | 910 | G | - | - | - | - | Glyco_hydro_43 |
| contig00025.g11910 | FVEG_13325 | 888 | O | - | - | - | - | Peptidase_S9 |
| contig00026.g11914 | FVEG_17495 | 85.5 | S | - | ko:K13449 | ko00000,ko00001 | - | CAP |
| contig00026.g11930 | FVEG_13310 | 1634 | E | 1.1.99.18 | ko:K19069 | ko00000,ko01000 | - | CBM_1,CDH-cyt,GMC_oxred_C,GMC_oxred_N |
| contig00026.g11934 | FVEG_13307 | 1081 | E | 1.1.99.18 | ko:K19069 | ko00000,ko01000 | - | GMC_oxred_C,GMC_oxred_N |
| contig00026.g11935 | FVEG_13306 | 783 | O | 3.4.23.25 | ko:K01381 | ko00000,ko00001,ko01000,ko01002 | - | Asp |
| contig00026.g11936 | FVEG_13305 | 946 | S | - | - | - | - | Glyco_hydro_39 |
| contig00026.g11937 | FVEG_13304 | 1074 | G | 3.2.1.59 | ko:K08254 | ko00000,ko01000 | - | Glyco_hydro_71 |
| contig00026.g11941 | FVEG_13300 | 800 | - | - | - | - | - | - |
| contig00026.g11951 | FVEG_13289 | 1132 | H | - | - | - | - | Amino_oxidase |
| contig00026.g11953 | FVEG_17483 | 421 | G | - | - | - | - | Lactonase |
| contig00026.g11981 | FVEG_13262 | 775 | O | - | - | - | - | BNR |
| contig00026.g11986 | FVEG_13256 | 1037 | S | - | - | - | - | PepX_C,Peptidase_S15 |
| contig00026.g11988 | FVEG_13254 | 1143 | - | - | - | - | - | - |
| contig00026.g11994 | FVEG_13248 | 750 | - | - | - | - | - | - |
| contig00026.g12035 | FVEG_13207 | 479 | G | 3.2.1.8 | ko:K01181 | ko00000,ko01000 | - | Glyco_hydro_11 |
| contig00026.g12057 | FVEG_13183 | 798 | O | 3.2.1.172 | ko:K15532 | ko00000,ko01000 | GH105 | Glyco_hydro_88 |
| contig00026.g12059 | FVEG_13181 | 611 | - | - | - | - | - | - |
| contig00026.g12064 | FVEG_13176 | 421 | - | - | - | - | - | - |
| contig00026.g12079 | FVEG_13160 | 867 | M | - | ko:K05747 | ko00000,ko00001,ko04131,ko04812 | - | Fasciclin |
| contig00027.g12245 | FVEG_08190 | 378 | S | - | - | - | - | DUF1524 |
| contig00027.g12280 | FVEG_16262 | 107 | - | - | - | - | - | - |
| contig00028.g12312 | FVEG_10761 | 138 | - | - | - | - | - | - |
| contig00028.g12315 | FVEG_10764 | 367 | - | - | - | - | - | - |
| contig00028.g12322 | FVEG_10770 | 379 | - | - | - | - | - | - |
| contig00028.g12347 | FVEG_10795 | 633 | G | 3.1.1.11 | ko:K01051 | ko00000,ko00001,ko00002,ko01000 | - | Pectinesterase |
| contig00028.g12348 | FVEG_10796 | 274 | - | - | - | - | - | - |
| contig00028.g12351 | FVEG_10798 | 895 | O | - | - | - | - | Abhydrolase_6,DUF1100 |
| contig00028.g12363 | FVEG_10812 | 443 | - | - | - | - | - | - |
| contig00028.g12365 | FVEG_10814 | 774 | O | - | - | - | - | Beta_helix,CBM_1,Chondroitinas_B |
| contig00028.g12387 | FVEG_10833 | 512 | S | - | - | - | - | CAP |
| contig00028.g12400 | FVEG_10845 | 827 | S | - | - | - | - | Beta_helix |
| contig00028.g12402 | FVEG_10848 | 308 | S | - | - | - | - | HsbA |
| contig00028.g12413 | FVEG_10860 | 634 | - | - | - | - | - | - |
| contig00028.g12415 | FVEG_10862 | 485 | E | - | - | - | - | Pectate_lyase |
| contig00028.g12416 | FVEG_10863 | 764 | O | - | - | - | - | Inhibitor_I9,Peptidase_S8 |
| contig00028.g12419 | FVEG_10867 | 1071 | C | - | - | - | - | BBE,FAD_binding_4 |
| contig00028.g12421 | FVEG_10869 | 333 | - | - | - | - | - | - |
| contig00028.g12446 | FVEG_10896 | 986 | S | - | - | - | - | DUF1593 |
| contig00028.g12447 | FVEG_10897 | 1030 | G | 3.2.1.91 | ko:K01225 | ko00000,ko00001,ko01000 | GH7 | Glyco_hydro_7 |
| contig00028.g12452 | FVEG_10901 | 1169 | G | 3.1.1.73 | ko:K09252 | ko00000,ko01000 | - | Tannase |
| contig00028.g12458 | FVEG_10907 | 643 | Q | 1.5.3.14,1.5.3.16 | ko:K13366 | ko00000,ko00001,ko01000 | - | Amino_oxidase |
| contig00028.g12476 | FVEG_10918 | 1238 | S | - | - | - | - | Glyco_hydro_127 |
| contig00028.g12485 | FVEG_10926 | 926 | S | 3.1.3.2 | ko:K01078 | ko00000,ko00001,ko01000 | - | His_Phos_2 |
| contig00028.g12487 | FVEG_10928 | 1005 | - | - | - | - | - | - |
| contig00028.g12495 | FVEG_10936 | 560 | A | 3.1.27.1 | ko:K01166 | ko00000,ko01000,ko03016 | - | Ribonuclease_T2 |
| contig00028.g12501 | FVEG_10941 | 527 | S | - | - | - | - | NPP1 |
| contig00028.g12518 | FVEG_10956 | 325 | - | - | - | - | - | - |
| contig00028.g12524 | FVEG_10963 | 580 | O | - | - | - | - | - |
| contig00029.g12594 | FVEG_07261 | 503 | G | 3.2.1.8 | ko:K01181 | ko00000,ko01000 | - | CBM_1,Glyco_hydro_11 |
| contig00029.g12624 | FVEG_07233 | 1104 | O | - | - | - | - | Peptidase_S28 |
| contig00029.g12625 | FVEG_07232 | 654 | O | 1.14.99.54 | ko:K19356 | ko00000,ko01000 | AA9,CBM1 | CBM_1,Glyco_hydro_61 |
| contig00029.g12630 | FVEG_07227 | 678 | E | 3.5.1.26 | ko:K01444 | ko00000,ko00001,ko01000 | - | Asparaginase_2 |
| contig00029.g12658 | FVEG_07203 | 332 | - | - | - | - | - | - |
| contig00029.g12663 | FVEG_07198 | 895 | O | - | - | - | - | Peptidase_M14 |
| contig00029.g12686 | FVEG_07174 | 412 | - | - | - | - | - | - |
| contig00029.g12687 | FVEG_07173 | 672 | S | - | - | - | - | Lipase_GDSL_2 |
| contig00029.g12688 | FVEG_07172 | 299 | S | - | - | - | - | Cerato-platanin |
| contig00029.g12689 | FVEG_07171 | 362 | - | - | - | - | - | - |
| contig00029.g12691 | FVEG_07168 | 375 | S | - | - | - | - | - |
| contig00029.g12692 | FVEG_07167 | 1125 | C | - | - | - | - | BBE,FAD_binding_4 |
| contig00030.g12704 | FVER53263_20291 | 80.1 | - | - | - | - | - | - |
| contig00030.g12716 | FVER53263_20411 | 324 | F | - | - | - | - | - |
| contig00030.g12739 | FVEG_12002 | 1764 | G | 3.2.1.14 | ko:K01183 | ko00000,ko00001,ko01000 | GH18 | Chitin_bind_1,Glyco_hydro_18,LysM |
| contig00030.g12741 | FVER53263_20903 | 507 | G | - | - | - | - | LysM |
| contig00030.g12755 | FVEG_01809 | 169 | - | - | - | - | - | - |
| contig00030.g12773 | FVEG_11126 | 253 | - | - | - | - | - | - |
| contig00030.g12800 | FVEG_13989 | 609 | S | - | - | - | - | SGL |
| contig00030.g12815 | FVEG_17692 | 204 | - | - | - | - | - | - |
| contig00030.g12826 | FVEG_17699 | 278 | - | - | - | - | - | - |
| contig00030.g12833 | FVEG_14021 | 319 | - | - | - | - | - | - |
| contig00031.g12848 | FVEG_03433 | 317 | S | - | - | - | - | - |
| contig00031.g12850 | FVEG_15264 | 266 | S | - | - | - | - | WSC |
| contig00031.g12857 | FVEG_03442 | 358 | - | - | - | - | - | - |
| contig00031.g12859 | FVEG_03444 | 594 | H | - | - | - | - | PBP_like_2 |
| contig00031.g12874 | FVEG_03457 | 1120 | S | - | - | - | - | Beta-lactamase |
| contig00031.g12880 | FVEG_03463 | 766 | G | 3.2.1.78 | ko:K19355 | ko00000,ko00001,ko01000 | - | Cellulase |
| contig00031.g12884 | FVEG_15271 | 446 | - | - | - | - | - | - |
| contig00031.g12887 | FVEG_12594 | 869 | G | - | - | - | - | Cellulase |
| contig00031.g12908 | FVEG_03492 | 532 | S | - | - | - | - | Kp4 |
| contig00031.g12910 | FVEG_03494 | 1143 | O | 3.4.14.9 | ko:K01279 | ko00000,ko00001,ko01000,ko01002,ko03110,ko04147 | - | Peptidase_S8,Pro-kuma_activ |
| contig00031.g12919 | FVEG_03505 | 124 | - | - | - | - | - | - |
| contig00031.g12920 | FVEG_03506 | 598 | O | - | - | - | - | Glyco_hydro_61 |
| contig00031.g12933 | FVEG_03519 | 467 | G | - | - | - | - | Pectate_lyase |
| contig00031.g12936 | FVEG_03522 | 510 | O | 3.1.1.86 | ko:K15530 | ko00000,ko01000 | - | CBM_1,Lipase_GDSL_2 |
| contig00031.g12937 | FVEG_03523 | 215 | S | - | - | - | - | Kp4 |
| contig00031.g12938 | FVEG_03524 | 259 | S | - | - | - | - | Kp4 |
| contig00031.g12939 | FVEG_03525 | 219 | S | - | - | - | - | Kp4 |
| contig00031.g12940 | FVEG_03526 | 1554 | E | 1.1.99.18 | ko:K19069 | ko00000,ko01000 | - | CDH-cyt,GMC_oxred_C,GMC_oxred_N |
| contig00031.g12942 | FVEG_03528 | 860 | - | - | - | - | - | - |
| contig00031.g12943 | FVEG_03529 | 209 | - | - | - | - | - | - |
| contig00031.g12983 | FVEG_03576 | 347 | - | - | - | - | - | - |
| contig00031.g12984 | FVEG_03577 | 1239 | O | - | - | - | - | ERO1 |
| contig00031.g12999 | FVEG_03591 | 361 | S | - | - | - | - | DUF3328 |
| contig00031.g13022 | FVEG_03617 | 616 | G | - | - | - | - | LysM |
| contig00031.g13025 | FVEG_03620 | 578 | - | - | - | - | - | - |
| contig00031.g13028 | FVEG_03623 | 521 | G | - | - | - | - | LysM |
| contig00032.g13061 | FVEG_01486 | 815 | S | - | ko:K01238 | ko00000,ko01000 | - | SUN |
| contig00032.g13126 | FVEG_01414 | 1257 | G | 3.2.1.52 | ko:K12373 | ko00000,ko00001,ko00002,ko01000,ko03110 | GH20 | Glyco_hydro_20,Glycohydro_20b2 |
| contig00032.g13164 | FVEG_01377 | 981 | O | 5.3.4.1 | ko:K09580 | ko00000,ko00001,ko01000,ko03110,ko04131,ko04147 | - | Thioredoxin,Thioredoxin_6 |
| contig00033.g13172 | FVEG_13757 | 476 | O | - | - | - | - | CBM_1,Lipase_GDSL_2 |
| contig00033.g13193 | FVEG_13736 | 687 | S | - | - | - | - | - |
| contig00033.g13199 | FVEG_13730 | 689 | S | 1.1.1.2 | ko:K00002 | ko00000,ko00001,ko00002,ko01000,ko04147 | - | Aldo_ket_red |
| contig00033.g13201 | FVEG_13728 | 479 | - | - | - | - | - | - |
| contig00033.g13217 | FVEG_13711 | 633 | G | 4.2.2.10 | ko:K01732 | ko00000,ko01000 | - | Pec_lyase_C |
| contig00033.g13221 | FVEG_13708 | 427 | - | - | - | - | - | - |
| contig00033.g13237 | FVEG_13692 | 379 | S | - | ko:K09705 | ko00000 | - | Cupin_5 |
| contig00033.g13244 | FVEG_13685 | 736 | E | 3.5.1.16 | ko:K01438 | ko00000,ko00001,ko00002,ko01000 | - | M20_dimer,Peptidase_M20,Peptidase_M28 |
| contig00033.g13255 | FVEG_13674 | 1317 | T | - | - | - | - | - |
| contig00033.g13260 | FVEG_13669 | 278 | - | - | - | - | - | - |
| contig00033.g13262 | FVEG_13668 | 353 | - | - | - | - | - | - |
| contig00033.g13274 | FVEG_13657 | 1547 | G | 3.2.1.6 | ko:K01180 | ko00000,ko01000 | - | Glyco_hydro_81 |
| contig00033.g13276 | FVEG_13655 | 350 | S | - | - | - | - | EthD |
| contig00033.g13289 | FVEG_13643 | 257 | - | - | - | - | - | - |
| contig00033.g13290 | FVEG_13642 | 293 | - | - | - | - | - | - |
| contig00033.g13295 | FVEG_13638 | 442 | O | 3.1.1.74 | ko:K08095 | ko00000,ko01000 | - | Cutinase |
| contig00033.g13299 | FVEG_11762 | 153 | IQ | - | - | - | - | AMP-binding,AMP-binding_C,Acyl_transf_1,Condensation,KAsynt_C_assoc,NAD_binding_4,PP-binding,ketoacyl-synt |
| contig00033.g13302 | FVEG_13630 | 1210 | O | - | ko:K01417 | ko00000,ko01000,ko01002 | - | FTP,Peptidase_M36 |
| contig00033.g13316 | FVEG_13614 | 979 | EO | - | - | - | - | Peptidase_S10 |
| contig00033.g13326 | FVEG_13605 | 818 | O | - | - | - | - | Asp |
| contig00034.g13348 | FVEG_12922 | 212 | - | - | - | - | - | - |
| contig00034.g13355 | FVEG_12928 | 895 | S | 3.1.3.2 | ko:K01078 | ko00000,ko00001,ko01000 | - | Phosphoesterase |
| contig00034.g13357 | FVEG_12930 | 801 | G | 3.2.1.4 | ko:K01179 | ko00000,ko00001,ko01000 | GH5,GH9 | CBM_1,Cellulase |
| contig00034.g13361 | FVEG_12936 | 741 | S | 1.14.18.1 | ko:K00505 | ko00000,ko00001,ko00002,ko01000 | - | Tyrosinase |
| contig00034.g13378 | alpha-N-arabinofuranosidase | 981 | G | 3.2.1.55 | ko:K20844 | ko00000,ko00001,ko01000 | CBM42,GH54 | AbfB,ArabFuran-catal |
| contig00034.g13383 | FVEG_12957 | 954 | G | 3.2.1.1 | ko:K01176 | ko00000,ko00001,ko01000 | GH13 | Alpha-amylase,CBM_20,DUF1966 |
| contig00034.g13390 | FVEG_12964 | 574 | O | - | - | - | - | Peptidase_M43 |
| contig00034.g13392 | FVEG_12965 | 554 | O | - | - | - | - | Glyco_hydro_16 |
| contig00034.g13413 | FVEG_17415 | 52.8 | S | - | - | - | - | - |
| contig00034.g13414 | FVEG_12983 | 806 | S | - | - | - | - | - |
| contig00034.g13439 | FVEG_13004 | 131 | - | - | - | - | - | - |
| contig00034.g13443 | FVEG_13008 | 2179 | O | - | - | - | - | Glyco_hydro_115 |
| contig00034.g13462 | FVER53590_30399 | 484 | S | - | - | - | - | Fungal_lectin |
| contig00034.g13492 | FVEG_13055 | 1474 | G | 3.2.1.21 | ko:K05349 | ko00000,ko00001,ko01000 | GH3 | Fn3-like,Glyco_hydro_3,Glyco_hydro_3_C |
| contig00034.g13515 | FVEG_13080 | 593 | S | - | - | - | - | DUF3129 |
| contig00035.g13536 | FVEG_04196 | 1157 | Q | - | - | - | - | Cu-oxidase,Cu-oxidase_2,Cu-oxidase_3 |
| contig00035.g13603 | FVEG_04262 | 367 | S | - | - | - | - | Hce2 |
| contig00035.g13607 | FVEG_04266 | 841 | G | - | ko:K03854,ko:K10967 | ko00000,ko00001,ko01000,ko01003 | GT15 | Glyco_transf_15 |
| contig00035.g13618 | FVEG_04278 | 756 | - | - | - | - | - | - |
| contig00036.g13641 | FVEG_13840 | 561 | - | - | - | - | - | - |
| contig00036.g13642 | FVEG_13841 | 753 | G | - | - | - | - | Beta_helix,Chondroitinas_B |
| contig00036.g13648 | FVEG_13846 | 144 | - | - | - | - | - | - |
| contig00036.g13649 | FVEG_13848 | 1454 | - | - | - | - | - | MU117 |
| contig00036.g13650 | FVEG_13849 | 1168 | G | 3.2.1.3 | ko:K01178 | ko00000,ko00001,ko01000 | GH15 | CBM_20,Glyco_hydro_15 |
| contig00036.g13654 | FVEG_13853 | 261 | - | - | - | - | - | - |
| contig00036.g13658 | FVEG_13856 | 66.6 | - | - | - | - | - | - |
| contig00036.g13666 | FVEG_13865 | 283 | S | - | - | - | - | Kp4 |
| contig00036.g13672 | FVEG_13872 | 332 | - | - | - | - | - | - |
| contig00036.g13678 | FVEG_13877 | 371 | V | - | - | - | - | CBM_4_9 |
| contig00036.g13681 | FVEG_17643 | 114 | - | - | - | - | - | - |
| contig00036.g13689 | FVEG_13886 | 909 | - | - | - | - | - | - |
| contig00036.g13690 | FVEG_13887 | 165 | S | - | - | - | - | - |
| contig00036.g13699 | FVEG_13897 | 855 | S | - | - | - | - | MmgE_PrpD |
| contig00036.g13705 | FVEG_13902 | 134 | - | - | - | - | - | - |
| contig00036.g13713 | FVEG_13909 | 797 | G | - | - | - | - | Lactonase |
| contig00036.g13718 | FVEG_13912 | 1093 | - | - | - | - | - | - |
| contig00036.g13728 | FVEG_13920 | 246 | - | - | - | - | - | - |
| contig00036.g13733 | FVEG_13926 | 1127 | T | - | - | - | - | COesterase |
| contig00036.g13745 | FVEG_13934 | 813 | O | 3.4.21.63 | ko:K18549 | ko00000,ko01000,ko01002 | - | Inhibitor_I9,Peptidase_S8 |
| contig00036.g13758 | FVEG_13947 | 200 | - | - | - | - | - | - |
| contig00036.g13759 | FVEG_13948 | 495 | S | - | - | - | - | DJ-1_PfpI |
| contig00036.g13760 | FVEG_13949 | 896 | S | - | - | - | - | EHN |
| contig00037.g13766 | FVEG_12913 | 978 | G | 3.2.1.123 | ko:K05991 | ko00000,ko01000 | GH5 | Cellulase,Ricin_B_lectin |
| contig00037.g13769 | FVEG_12910 | 804 | G | 3.1.1.17 | ko:K01053 | ko00000,ko00001,ko00002,ko01000,ko04147 | - | SGL |
| contig00037.g13776 | FVEG_12905 | 868 | S | - | - | - | - | LIP |
| contig00037.g13792 | FVEG_12888 | 1574 | Q | 1.11.1.21 | ko:K03782 | ko00000,ko00001,ko01000 | - | peroxidase |
| contig00037.g13801 | FVEG_12879 | 85.9 | S | - | - | - | - | - |
| contig00037.g13802 | FVEG_12878 | 1040 | O | 3.4.16.5 | ko:K13289 | ko00000,ko00001,ko01000,ko01002,ko03110,ko04131,ko04147 | - | Peptidase_S10 |
| contig00037.g13824 | FVEG_12856 | 679 | - | - | - | - | - | LPMO_10 |
| contig00037.g13842 | FVEG_12840 | 650 | O | - | - | - | - | Glyco_hydro_16 |
| contig00037.g13861 | FVEG_12823 | 1055 | Q | 1.5.3.14,1.5.3.16 | ko:K13366 | ko00000,ko00001,ko01000 | - | Amino_oxidase |
| contig00038.g13872 | FVEG_13759 | 627 | G | 3.2.1.39,3.2.1.58 | ko:K01199,ko:K01210 | ko00000,ko00001,ko00537,ko01000 | GH17 | Glyco_hydro_17 |
| contig00038.g13878 | FVEG_17600 | 1923 | G | 3.2.1.14 | ko:K01183 | ko00000,ko00001,ko01000 | GH18 | Chitin_bind_1,Glyco_hydro_18 |
| contig00038.g13879 | FVEG_13765 | 785 | S | - | - | - | - | LysM |
| contig00038.g13880 | FVEG_13766 | 1270 | G | - | - | - | - | LysM |
| contig00038.g13889 | FVEG_13776 | 712 | S | 1.14.18.1 | ko:K00505 | ko00000,ko00001,ko00002,ko01000 | - | Tyrosinase |
| contig00038.g13901 | FVEG_13788 | 260 | - | - | - | - | - | - |
| contig00038.g13923 | FVEG_13811 | 754 | O | 3.4.21.63 | ko:K18549 | ko00000,ko01000,ko01002 | - | Inhibitor_I9,Peptidase_S8 |
| contig00038.g13925 | FVEG_13813 | 527 | S | - | - | - | - | Glyco_hydro_114 |
| contig00038.g13929 | FVEG_17613 | 1650 | G | 3.2.1.165 | ko:K15855 | ko00000,ko00001,ko01000 | - | Glyco_hydro_2,Glyco_hydro_2_C,Glyco_hydro_2_N |
| contig00038.g13931 | FVEG_13819 | 507 | O | 3.5.1.104 | ko:K22278 | ko00000,ko01000 | - | Chitin_bind_1,Polysacc_deac_1 |
| contig00038.g13935 | FVEG_13823 | 1175 | G | 3.1.1.20 | ko:K10759 | ko00000,ko01000 | - | Tannase |
| contig00038.g13937 | FVEG_17615 | 52.8 | E | - | - | - | - | PAN_4 |
| contig00038.g13940 | FVEG_13826 | 662 | - | - | - | - | - | SGL |
| contig00038.g13946 | FVEG_13832 | 714 | - | - | - | - | - | - |
| contig00038.g13947 | FVEG_13833 | 452 | - | - | - | - | - | - |
| contig00038.g13948 | FVEG_13834 | 501 | - | - | - | - | - | - |
| contig00039.g13978 | FVEG_05076 | 400 | S | - | - | - | - | CHRD |
| contig00039.g13985 | FVEG_05084 | 983 | O | - | ko:K09523 | ko00000,ko00001,ko03110 | - | DnaJ,TPR_16,TPR_19,TPR_8 |
| contig00040.g14015 | FVEG_11410 | 1080 | S | - | - | - | - | Arylsulfotran_2 |
| contig00040.g14028 | FVEG_11396 | 188 | - | - | - | - | - | - |
| contig00041.g14044 | FVEG_13986 | 2040 | - | - | - | - | - | - |
| contig00041.g14065 | FVEG_13966 | 289 | S | - | - | - | - | - |
| contig00041.g14068 | FVEG_13964 | 272 | S | - | - | - | - | Kp4 |
| contig00041.g14076 | FVEG_13957 | 691 | G | 4.2.2.2 | ko:K01728 | ko00000,ko00001,ko01000 | - | Pec_lyase_C |
| contig00043.g14114 | FVEG_04535 | 321 | S | - | - | - | - | CM_2 |
| contig00043.g14137 | FVEG_14092 | 354 | S | - | - | - | - | - |
| contig00043.g14138 | FVEG_14091 | 1083 | G | - | - | - | - | COesterase |
| contig00001.g1263 | FVEG_01295 | - | - | - | - | - | - | - |
| contig00003.g2176 | FVEG_05714 | - | - | - | - | - | - | - |
| contig00004.g3188 | FVEG_03382 | - | - | - | - | - | - | - |
| contig00004.g3223 | FVEG_03292 | - | - | - | - | - | - | - |
| contig00004.g3227 | FVEG_15235 | - | - | - | - | - | - | - |
| contig00004.g3219 | FVEG_03290 | - | - | - | - | - | - | - |
| contig00004.g3463 | FVEG_16576 | - | - | - | - | - | - | - |
| contig00004.g3499 | FVEG_09444 | - | - | - | - | - | - | - |
| contig00005.g3620 | FVEG_16681 | - | - | - | - | - | - | - |
| contig00005.g3721 | FVEG_07105 | - | - | - | - | - | - | - |
| contig00007.g4833 | FVEG_03682 | - | - | - | - | - | - | - |
| contig00007.g4834 | FVEG_03683 | - | - | - | - | - | - | - |
| contig00007.g4844 | FVEG_03690 | - | - | - | - | - | - | - |
| contig00012.g7511 | FVEG_05945 | - | - | - | - | - | - | - |
| contig00012.g7513 | FVEG_15768 | - | - | - | - | - | - | - |
| contig00014.g8484 | FVEG_07751 | - | - | - | - | - | - | - |
| contig00018.g9760 | FVEG_11959 | - | - | - | - | - | - | - |
| contig00019.g10121 | FVEG_17224 | - | - | - | - | - | - | - |
| contig00020.g10432 | FVEG_12670 | - | - | - | - | - | - | - |
| contig00020.g10490 | FVEG_17310 | - | - | - | - | - | - | - |
| contig00020.g10510 | FVEG_17305 | - | - | - | - | - | - | - |
| contig00021.g10718 | FVEG_01879 | - | - | - | - | - | - | - |
| contig00022.g10899 | FVEG_16828 | - | - | - | - | - | - | - |
| contig00022.g11027 | FVEG_16861 | - | - | - | - | - | - | - |
| contig00028.g12316 | FVEG_10765 | - | - | - | - | - | - | - |
| contig00030.g12789 | FVEG_09724 | - | - | - | - | - | - | - |
| contig00030.g12825 | FVEG_14015 | - | - | - | - | - | - | - |
| contig00030.g12837 | FVEG_14025 | - | - | - | - | - | - | - |
| contig00031.g12882 | FVEG_03465 | - | - | - | - | - | - | - |
| contig00031.g13027 | FVEG_15301 | - | - | - | - | - | - | - |
| contig00033.g13263 | FVEG_13667 | - | - | - | - | - | - | - |
| contig00033.g13343 | FVEG_13589 | - | - | - | - | - | - | - |
| contig00034.g13415 | FVEG_17416 | - | - | - | - | - | - | - |
| contig00034.g13416 | FVEG_12984 | - | - | - | - | - | - | - |
| contig00034.g13425 | FVEG_17418 | - | - | - | - | - | - | - |

**Supplementary Table 3.** Main proteins in STRING interactions with a subset of selected secreted proteins from *F. verticillioides* (DA42).

| **gene name** | **protein** | **stringId** | **annotation** | **Gene name** | **stringId** | **score** | **Protein names** |
| --- | --- | --- | --- | --- | --- | --- | --- |
| FVEG_13935 | XP_018762357 | 334819.A0A139YBL9 | FAD dependent oxidoreductase domain-containing protein | FVEG_03983 | 334819.W7LTY4 | 0.713 | DASH complex subunit SPC19 (Outer kinetochore protein SPC19) |
|  |  |  |  | FVEG_02338 | 334819.W7LJJ9 | 0.705 | Glycine cleavage system H protein |
|  |  |  |  | FVEG_06633 | 334819.W7MMZ9 | 0.705 | Glycine cleavage system H protein |
|  |  |  |  | FVEG_02337 | 334819.W7M3V6 | 0.701 | Glycine cleavage system P protein (EC 1.4.4.2) |
|  |  |  |  | FVEG_06405 | 334819.W7MDL7 | 0.642 | Spindle pole body component |
|  |  |  |  | FVEG_00878 | 334819.W7LX51 | 0.62 | Methylenetetrahydrofolate dehydrogenase [NAD(+)] (EC 1.5.1.15) |
|  |  |  |  | FVEG_09630 | 334819.W7MHT7 | 0.608 | DASH complex subunit DAD2 (Outer kinetochore protein DAD2) |
|  |  |  |  | FVEG_12150 | 334819.W7N0W4 | 0.571 | DASH complex subunit DAM1 (Outer kinetochore protein DAM1) |
|  |  |  |  | FVEG_02585 | 334819.W7LKR4 | 0.554 | Serine hydroxymethyltransferase (EC 2.1.2.1) |
|  |  |  |  | FVEG_02339 | 334819.W7LVX0 | 0.554 | Serine hydroxymethyltransferase (EC 2.1.2.1) |
| FVEG_03246 | XP_018747256 | 334819.W7LR99 | G-patch domain-containing protein | FVEG_08262 | 334819.W7MBZ1 | 0.987 | Cell cycle control protein cwf14 |
|  |  |  |  | FVEG_01430 | 334819.W7LF49 | 0.986 | Pre-mRNA-splicing factor clf-1 |
|  |  |  |  | FVEG_03791 | 334819.W7M2L0 | 0.971 | Elongation factor 2 |
|  |  |  |  | FVEG_09212 | 334819.W7MPY9 | 0.955 | Pre-mRNA-splicing factor spp42 |
|  |  |  |  | FVEG_09474 | 334819.W7MQZ0 | 0.954 | Anaphase-promoting complex subunit 4-like WD40 domain |
|  |  |  |  | FVEG_04224 | 334819.W7M4H1 | 0.942 | U2 small nuclear ribonucleoprotein A' |
|  |  |  |  | FVEG_10476 | 334819.W7MJI0 | 0.924 | Pre-mRNA-splicing factor SYF1 (Pre-mRNA-splicing factor syf1) |
|  |  |  |  | FVEG_05253 | 334819.W7LXQ4 | 0.913 | Pre-mRNA-splicing factor SYF2 |
|  |  |  |  | FVEG_07868 | 334819.W7M8J0 | 0.908 | Splicing factor YJU2 |
|  |  |  |  | FVEG_01212 | 334819.W7LQ92 | 0.897 | Sm protein B |
| FVEG_00028 | XP_018742012 | 334819.W7LTB5 | Major facilitator superfamily (MFS) profile domain-containing protein | FVEG_04927 | 334819.W7M7A8 | 0.342 | Glyceraldehyde-3-phosphate dehydrogenase (EC 1.2.1.12) |
|  |  |  |  | FVEG_11098 | 334819.W7MWZ5 | 0.289 | Phosphopantothenoylcysteine decarboxylase |
|  |  |  |  | FVEG_00804 | 334819.W7LNI3 | 0.286 | mRNA 3'-end-processing protein RNA14 |
|  |  |  |  | FVEG_04687 | 334819.W7M651 | 0.286 | 2,3-bisphosphoglycerate-independent phosphoglycerate mutase (EC 5.4.2.12) |
|  |  |  |  | FVEG_01159 | 334819.W7LQ36 | 0.283 | 2,3-bisphosphoglycerate-independent phosphoglycerate mutase (EC 5.4.2.12) |
|  |  |  |  | FVEG_04599 | 334819.W7LWH5 | 0.277 | Nucleoside diphosphate kinase (EC 2.7.4.6) |
| FVEG_08735 | XP_018755318 | 334819.W7MCA2 | Major facilitator superfamily (MFS) profile domain-containing protein | FVEG_07352 | 334819.W7MHX8 | 0.335 | Uncharacterized protein |
|  |  |  |  | FVEG_01463 | 334819.W7LI48 | 0.257 | SnoaL-like domain-containing protein |
|  |  |  |  | FVEG_10084 | 334819.W7MGZ3 | 0.249 | Gpi-anchored protein |
|  |  |  |  | FVEG_14849 | 334819.W7M007 | 0.247 | L-dopachrome isomerase (EC 5.3.2.1) (EC 5.3.3.12) |
|  |  |  |  | FVEG_16012 | 334819.W7MGI2 | 0.246 | DUF2415 domain-containing protein |
|  |  |  |  | FVEG_06112 | 334819.W7MKQ2 | 0.223 | DUF2415 domain-containing protein |
|  |  |  |  | FVEG_06550 | 334819.W7ME81 | 0.213 | Zn(2)-C6 fungal-type domain-containing protein |
|  |  |  |  | FVEG_17448 | 334819.W7MU61 | 0.21 | F-box domain-containing protein |
|  |  |  |  | FVEG_02771 | 334819.W7LXV8 | 0.205 | F-box domain-containing protein |
|  |  |  |  | FVEG_02376 | 334819.W7LW18 | 0.181 | Acetyl-CoA transporter |
| FVEG_09361 | XP_018756214 | 334819.W7MEN7 | feruloyl esterase (EC 3.1.1.73) | FVEG_16566 | 334819.W7MQL8 | 0.372 | AB hydrolase-1 domain-containing protein |
|  |  |  |  | FVEG_05363 | 334819.W7M9X5 | 0.277 | Oxidoreductase |
|  |  |  |  | FVEG_02675 | 334819.W7LNV1 | 0.272 | Aldehyde dehydrogenase |
|  |  |  |  | FVEG_06733 | 334819.W7MND6 | 0.272 | Aldehyde dehydrogenase domain-containing protein |
|  |  |  |  | FVEG_10644 | 334819.W7MVN0 | 0.272 | Aldehyde dehydrogenase domain-containing protein |
|  |  |  |  | FVEG_01702 | 334819.W7LIZ4 | 0.272 | Aldehyde dehydrogenase |
|  |  |  |  | FVEG_10643 | 334819.W7MVC5 | 0.271 | Extradiol ring-cleavage dioxygenase class III enzyme subunit B domain |
|  |  |  |  | FVEG_09411 | 334819.W7MR24 | 0.269 | aldehyde dehydrogenase (NAD(+)) (EC 1.2.1.3) |
|  |  |  |  | FVEG_02451 | 334819.W7M4A2 | 0.269 | Zn(2)-C6 fungal-type domain-containing protein |
|  |  |  |  | FVEG_06508 | 334819.W7MDY9 | 0.269 | aldehyde dehydrogenase (NAD(+)) (EC 1.2.1.3) |
| FVEG_05849 | XP_018751073 | 334819.W7MJP3 | Agglutinin-like protein 2 | FVEG_04117 | 334819.W7LSL8 | 0.812 | B30.2/SPRY domain-containing protein |
|  |  |  |  | FVEG_00682 | 334819.W7LMZ5 | 0.802 | Uncharacterized protein |
|  |  |  |  | FVEG_08130 | 334819.W7MBB6 | 0.788 | COMPASS component SDC1 |
|  |  |  |  | FVEG_07117 | 334819.W7M708 | 0.774 | Uncharacterized protein |
|  |  |  |  | FVEG_06109 | 334819.W7MKP6 | 0.716 | PH domain-containing protein |
|  |  |  |  | FVEG_07811 | 334819.W7MJH0 | 0.716 | Histone-lysine N-methyltransferase, H3 lysine-4 specific (EC 2.1.1.354) |
|  |  |  |  | FVEG_07326 | 334819.W7M7X6 | 0.571 | Transcriptional adapter 2-alpha |
|  |  |  |  | FVEG_00630 | 334819.W7LMU0 | 0.567 | Actin-related protein 4 |
|  |  |  |  | FVEG_11384 | 334819.W7MMM5 | 0.541 | DM2 domain-containing protein |
|  |  |  |  | FVEG_07033 | 334819.W7MML3 | 0.469 | Histone H4 |
| FVEG_10795 | XP_018758142 | 334819.W7MLJ0 | Pectinesterase (EC 3.1.1.11) | FVEG_08451 | 334819.W7MCP5 | 0.933 | endo-polygalacturonase (EC 3.2.1.15) (Pectinase) |
|  |  |  |  | FVEG_11787 | 334819.W7NA23 | 0.933 | endo-polygalacturonase (EC 3.2.1.15) (Pectinase) |
|  |  |  |  | FVEG_08734 | 334819.W7MXM7 | 0.915 | Pectate lyase (EC 4.2.2.2) |
|  |  |  |  | FVEG_13516 | 334819.W7N632 | 0.913 | endo-polygalacturonase (EC 3.2.1.15) (Pectinase) |
|  |  |  |  | FVEG_11233 | 334819.W7MN59 | 0.909 | Pectate lyase domain-containing protein |
|  |  |  |  | FVEG_03818 | 334819.W7LRH8 | 0.909 | pectate lyase (EC 4.2.2.2) |
|  |  |  |  | FVEG_12279 | 334819.W7N1B7 | 0.909 | pectate lyase (EC 4.2.2.2) |
|  |  |  |  | FVEG_09387 | 334819.W7MGQ3 | 0.905 | Pectate lyase (EC 4.2.2.2) |
|  |  |  |  | FVEG_10101 | 334819.W7MTD1 | 0.903 | pectate lyase (EC 4.2.2.2) |
|  |  |  |  | FVEG_10862 | 334819.W7MLS5 | 0.901 | Pectate lyase (EC 4.2.2.2) |
| FVEG_09702 | XP_018756690 | 334819.W7MRY6 | Pectin lyase (EC 4.2.2.10) | FVEG_10535 | 334819.W7N4R1 | 0.387 | Uncharacterized protein |
|  |  |  |  | FVEG_01690 | 334819.W7LGE0 | 0.358 | Iron transport multicopper oxidase FET3 |
|  |  |  |  | FVEG_01770 | 334819.W7LSX5 | 0.341 | Glucanase (EC 3.2.1.-) |
|  |  |  |  | FVEG_11600 | 334819.W7MPV6 | 0.336 | Expansin-like EG45 domain-containing protein |
|  |  |  |  | FVEG_10241 | 334819.W7MTU1 | 0.336 | Expansin-like EG45 domain-containing protein |
|  |  |  |  | FVEG_12563 | 334819.W7N2G8 | 0.336 | Pisatin demethylase |
|  |  |  |  | FVEG_04458 | 334819.W7M558 | 0.309 | MFS transporter, SIT family, siderophore-iron:H+ symporter |
|  |  |  |  | FVEG_06206 | 334819.W7M350 | 0.287 | Calmodulin |
|  |  |  |  | FVEG_07574 | 334819.W7M7F0 | 0.287 | Uncharacterized protein |
|  |  |  |  | FVEG_07362 | 334819.W7MRC4 | 0.287 | Calmodulin |
| FVEG_13183 | XP_018761329 | 334819.W7MUF4 | Cell wall glycosyl hydrolase YteR | FVEG_12180 | 334819.W7N0X5 | 0.366 | SGNH hydrolase-type esterase domain-containing protein |
|  |  |  |  | FVEG_00136 | 334819.W7LTR0 | 0.361 | SGNH hydrolase-type esterase domain-containing protein |
|  |  |  |  | FVEG_03522 | 334819.W7LQF7 | 0.36 | Uncharacterized protein |
|  |  |  |  | FVEG_13757 | 334819.W7N6S9 | 0.341 | SGNH hydrolase-type esterase domain-containing protein |
|  |  |  |  | FVEG_05696 | 334819.W7MAU3 | 0.296 | Sulfatase N-terminal domain-containing protein |
|  |  |  |  | FVEG_08588 | 334819.W7MMG8 | 0.296 | Zn(2)-C6 fungal-type domain-containing protein |
|  |  |  |  | FVEG_16305 | 334819.W7MW47 | 0.296 | Sulfatase N-terminal domain-containing protein |
|  |  |  |  | FVEG_10001 | 334819.W7MGS8 | 0.296 | Sulfatase N-terminal domain-containing protein |
|  |  |  |  | FVEG_00102 | 334819.W7LKF3 | 0.296 | Sulfatase N-terminal domain-containing protein |
|  |  |  |  | FVEG_09578 | 334819.W7MHH9 | 0.296 | Choline-sulfatase |
| FVEG_09388 | XP_018756247 | 334819.W7N040 | D/NAD(P)-binding domain-containing protein. | FVEG_04071 | 334819.W7M3P7 | 0.336 | Tyrosinase copper-binding domain-containing protein |
|  |  |  |  | FVEG_08860 | 334819.W7ME88 | 0.336 | FAD-binding PCMH-type domain-containing protein |
|  |  |  |  | FVEG_02919 | 334819.W7M6P3 | 0.336 | Methyltransferase |
|  |  |  |  | FVEG_06165 | 334819.W7M2U8 | 0.336 | Methyltransferase domain-containing protein |
|  |  |  |  | LAE1 | 334819.W7LAD1 | 0.331 | Secondary metabolism regulator LAE1 (EC 2.1.1.-) |
|  |  |  |  | FUS7 | 334819.W7MWX4 | 0.33 | Putative aldehyde dehydrogenase FUS7 (EC 1.2.1.3) |
|  |  |  |  | FUS5 | 334819.W7MWX7 | 0.311 | Esterase FUS5 (EC 3.1.2.-) (Fusarin biosynthesis protein 5) |
|  |  |  |  | FVEG_15504 | 334819.W7M5Y4 | 0.276 | TauD/TfdA-like domain-containing protein |
|  |  |  |  | FVEG_04736 | 334819.W7MED9 | 0.276 | trimethyllysine dioxygenase (EC 1.14.11.8) |
|  |  |  |  | FVEG_07548 | 334819.W7MIL5 | 0.276 | TauD/TfdA-like domain-containing protein |
| FVEG_03005 | XP_018746906.1 | 334819.W7LZI8 | Protein SnodProt1 | FVEG_00750 | 334819.W7LBK3 | 0.354 | Elongin-C |
|  |  |  |  | FVEG_05429 | 334819.W7LYE3 | 0.243 | Uncharacterized protein |
|  |  |  |  | FVEG_04213 | 334819.W7LTA5 | 0.243 | Major histocompatibility complex, class I |
|  |  |  |  | FVEG_00181 | 334819.W7LBP4 | 0.242 | Mitochondrial 40S ribosomal protein MRP2 |
|  |  |  |  | FVEG_09167 | 334819.W7MQ27 | 0.223 | RING-box protein 1 |
|  |  |  |  | FVEG_00621 | 334819.W7LVZ1 | 0.191 | Transcription initiation factor TFIID subunit 9 |
|  |  |  |  | FVEG_05820 | 334819.W7MBD8 | 0.186 | non-specific serine/threonine protein kinase (EC 2.7.11.1) |
|  |  |  |  | FVEG_06407 | 334819.W7M419 | 0.173 | Bms1-type G domain-containing protein |
|  |  |  |  | FVEG_01096 | 334819.W7LPT4 | 0.171 | Ubiquitin-conjugating enzyme E2C-binding protein |
|  |  |  |  | FVEG_07614 | 334819.W7M7J3 | 0.171 | BAR domain-containing protein |
| FVEG_05642 | XP_018750821 | 334819.W7MAL2 | Chitin-binding type-4 domain-containing protein | FVEG_12434 | 334819.W7MRR1 | 0.224 | Pectate lyase (EC 4.2.2.2) |
|  |  |  |  | FVEG_03519 | 334819.W7M1N7 | 0.221 | Pectate lyase (EC 4.2.2.2) |
|  |  |  |  | FVEG_08734 | 334819.W7MXM7 | 0.205 | Pectate lyase (EC 4.2.2.2) |
|  |  |  |  | FVEG_09387 | 334819.W7MGQ3 | 0.2 | Pectate lyase (EC 4.2.2.2) |
| FVEG_09149 | XP_018755879 | 334819.W7MDP1 | Peroxidase (EC 1.11.1.-) | FVEG_04068 | 334819.W7LU87 | 0.723 | Ribonuclease H2 subunit B (Ribonuclease HI subunit B) |
|  |  |  |  | FVEG_00724 | 334819.W7LWI4 | 0.605 | Ribonuclease (EC 3.1.26.4) |
|  |  |  |  | FVEG_01355 | 334819.W7LHJ0 | 0.448 | F-actin-capping protein subunit beta |
|  |  |  |  | FVEG_04864 | 334819.W7M791 | 0.433 | GTPase-activating protein GYP7 (GAP for YPT7) |
|  |  |  |  | FVEG_06157 | 334819.W7M172 | 0.334 | GTPase-activating protein beta-chimerin |
|  |  |  |  | FVEG_05294 | 334819.W7LXW4 | 0.333 | Alpha-actinin |
|  |  |  |  | FVEG_09191 | 334819.W7MFR1 | 0.333 | CRIB domain-containing protein |
|  |  |  |  | FVEG_05170 | 334819.W7MGL3 | 0.319 | Bicarbonate transporter-like transmembrane domain |
|  |  |  |  | FVEG_14576 | 334819.W7LK77 | 0.319 | Bicarbonate transporter-like transmembrane domain |
|  |  |  |  | FVEG_02190 | 334819.W7LV02 | 0.319 | Bicarbonate transporter-like transmembrane domain |
| FVEG_04647 | EWG42982 | 334819.W7ME03 | Necrosis inducing protein | FVEG_04937 | 334819.W7M7C5 | 0.49 | Cytochrome c oxidase subunit |
|  |  |  |  | FVEG_02595 | 334819.W7M563 | 0.446 | Uncharacterized protein |
|  |  |  |  | cox3 | 334819.H6D5G9 | 0.437 | Cytochrome c oxidase subunit 3 |
|  |  |  |  | cox2 | 334819.H6D5F6 | 0.436 | Cytochrome c oxidase subunit 2 |
|  |  |  |  | FVEG_09751 | 334819.W7MFY5 | 0.435 | Cytochrome c oxidase subunit 8, mitochondrial |
|  |  |  |  | FVEG_02487 | 334819.W7M4D9 | 0.431 | Cytochrome c oxidase subunit 6a |
|  |  |  |  | FVEG_07914 | 334819.W7M8Q0 | 0.415 | Cytochrome c oxidase subunit 6, mitochondrial |
|  |  |  |  | FVEG_08991 | 334819.W7MEW5 | 0.406 | Uncharacterized protein |
|  |  |  |  | FVEG_08989 | 334819.W7MNZ2 | 0.406 | Cytochrome c oxidase subunit 4, mitochondrial |
|  |  |  |  | FVEG_11349 | 334819.W7MY15 | 0.384 | Cytochrome c oxidase polypeptide V |
